# Matrotrophy and placentation in invertebrates: a new paradigm

**DOI:** 10.1111/brv.12189

**Published:** 2015-04-29

**Authors:** Andrew N. Ostrovsky, Scott Lidgard, Dennis P. Gordon, Thomas Schwaha, Grigory Genikhovich, Alexander V. Ereskovsky

**Affiliations:** ^1^ Department of Invertebrate Zoology, Faculty of BiologySaint Petersburg State UniversityUniversitetskaja nab. 7/9199034Saint PetersburgRussia; ^2^ Department of Palaeontology, Faculty of Earth Sciences, Geography and Astronomy, GeozentrumUniversity of ViennaAlthanstrasse 14A‐1090ViennaAustria; ^3^ Integrative Research CenterField Museum of Natural History1400 S. Lake Shore Dr.ChicagoIL60605U.S.A.; ^4^ National Institute of Water and Atmospheric ResearchPrivate Bag 14901, KilbirnieWellingtonNew Zealand; ^5^ Department of Integrative Zoology, Faculty of Life SciencesUniversity of ViennaAlthanstrasse 14A‐1090ViennaAustria; ^6^ Department for Molecular Evolution and Development, Faculty of Life SciencesUniversity of ViennaAlthanstrasse 14A‐1090ViennaAustria; ^7^ Department of Embryology, Faculty of BiologySaint Petersburg State UniversityUniversitetskaja nab. 7/9199034Saint PetersburgRussia; ^8^ Institut Méditerranéen de Biodiversité et d'Ecologie marine et continentale, Aix Marseille Université, CNRS, IRD, Avignon UniversitéStation marine d'Endoume, Chemin de la Batterie des Lions13007MarseilleFrance

**Keywords:** matrotrophy, viviparity, brooding, placenta, invertebrates, convergent evolution

## Abstract

Matrotrophy, the continuous extra‐vitelline supply of nutrients from the parent to the progeny during gestation, is one of the masterpieces of nature, contributing to offspring fitness and often correlated with evolutionary diversification. The most elaborate form of matrotrophy—placentotrophy—is well known for its broad occurrence among vertebrates, but the comparative distribution and structural diversity of matrotrophic expression among invertebrates is wanting. In the first comprehensive analysis of matrotrophy across the animal kingdom, we report that regardless of the degree of expression, it is established or inferred in at least 21 of 34 animal phyla, significantly exceeding previous accounts and changing the old paradigm that these phenomena are infrequent among invertebrates. In 10 phyla, matrotrophy is represented by only one or a few species, whereas in 11 it is either not uncommon or widespread and even pervasive. Among invertebrate phyla, Platyhelminthes, Arthropoda and Bryozoa dominate, with 162, 83 and 53 partly or wholly matrotrophic families, respectively. In comparison, Chordata has more than 220 families that include or consist entirely of matrotrophic species. We analysed the distribution of reproductive patterns among and within invertebrate phyla using recently published molecular phylogenies: matrotrophy has seemingly evolved at least 140 times in all major superclades: Parazoa and Eumetazoa, Radiata and Bilateria, Protostomia and Deuterostomia, Lophotrochozoa and Ecdysozoa. In Cycliophora and some Digenea, it may have evolved twice in the same life cycle. The provisioning of developing young is associated with almost all known types of incubation chambers, with matrotrophic viviparity more widespread (20 phyla) than brooding (10 phyla). In nine phyla, both matrotrophic incubation types are present. Matrotrophy is expressed in five nutritive modes, of which histotrophy and placentotrophy are most prevalent. Oophagy, embryophagy and histophagy are rarer, plausibly evolving through heterochronous development of the embryonic mouthparts and digestive system. During gestation, matrotrophic modes can shift, intergrade, and be performed simultaneously. Invertebrate matrotrophic adaptations are less complex structurally than in chordates, but they are more diverse, being formed either by a parent, embryo, or both. In a broad and still preliminary sense, there are indications of trends or grades of evolutionarily increasing complexity of nutritive structures: formation of (i) local zones of enhanced nutritional transport (placental analogues), including specialized parent–offspring cell complexes and various appendages increasing the entire secreting and absorbing surfaces as well as the contact surface between embryo and parent, (ii) compartmentalization of the common incubatory space into more compact and ‘isolated’ chambers with presumably more effective nutritional relationships, and (iii) internal secretory (‘milk’) glands. Some placental analogues in onychophorans and arthropods mimic the simplest placental variants in vertebrates, comprising striking examples of convergent evolution acting at all levels—positional, structural and physiological.

## I. INTRODUCTION

Modes of reproduction and the timing and manner of nutrient provisioning to developing embryos are life‐history traits that profoundly affect survival and evolutionary fitness (Marshall, Allen & Crean, 2008; Pollux *et al*., 2009; Lodé, 2012). For most sexual animals, fertilized eggs develop and hatch in the external environment. But this pattern is far from universal; developing progeny may also be retained inside or on the parent. In a number of clades, conventional theories of evolutionary transitions to the retention of progeny implicate enhanced survival of incubated young (Avise, 2013). Retention, and thus close contact between the tissues of the parent and developing embryo, may have become associated with exchange of gases and water. While many incubating species make use of egg yolk alone as the source of nourishment for embryo development (termed lecithotrophy), in some others incubation of the progeny led to the evolution of matrotrophy. Matrotrophy is the more or less continuous parental extra‐vitelline provision of nutrients during gestation. In fact, physiological relationships between the parent and developing offspring—embryo, larva or juvenile—imply a bidirectional transfer of nutrients and metabolic wastes, although waste removal is much less studied, and has seldom been mentioned in animals (Moosbrugger *et al*., 2012). Matrotrophy is also sometimes referred to as extraembryonic nutrition (EEN), although the latter term is narrower (see Section II). Under either term, this phenomenon is very familiar to us in a particular and most complex form, placentotrophy, in which nutrition is provided *via* a placenta. Other expressions of matrotrophy include embryonic absorption or ingestion of nutrient secretions in uterine or other incubatory spaces, and consumption of maternal tissues, eggs or sibling embryos (Wourms, 1981; Wourms, Grove & Lombardi, 1988; 1999c, 2014; Avise, 2013; see Sections II, IV.4 and IV.5 for definitions and details).

Matrotrophy is typically associated with viviparity—development of the embryo within the reproductive system, body cavity, or parental tissues, resulting in live birth. The multiple origins of matrotrophy and viviparity surely rank among the grandest examples of evolutionary convergence and are often correlated with taxonomic diversification (Angelini & Ghiara, 1984; Blackburn, 1992, 2005, 2014; Reynolds, Goodwin & Freckleton, 2002; Crespi & Semeniuk, 2004; Von Rintelen & Glaubrecht, 2005; Elliot & Crespi, 2009). Yet despite the affirmed ecological and evolutionary importance of gestational mode, the terminology of embryonic incubation varies among authors and disciplinary specialties, and definitions run the gamut from restrictive to broadly permissive (Blackburn, 1992; Wake, 1992; Lodé, 2012; Avise, 2013). Here, for heuristic purposes we separate viviparity (as defined above) from brooding, which we distinguish as embryonic incubation on the body surface, inside its infoldings, invaginations, or gastric system (Trumbo, 2012; see Section II). Our focus is on the broad range of matrotrophy, with placentotrophy as an essential part.

Most theories of the adaptive significance of and impediments to matrotrophy stem from work on vertebrates, which constitutes the overwhelming majority of studies (reviewed in Blackburn, 2005, 2014). However, too narrow a range of nature's diversity may be insufficient to realize the phenomena to be explained—the genetics, physiology, ecology, and evolution of matrotrophy among animals. Here we report the results of the first extensive literature analyses, augmented by our own anatomical and ultrastructural studies, which reveal an astonishingly wide distribution of matrotrophy and placentation throughout Animalia, in contrast to a more traditional view that these phenomena are infrequent among invertebrates (see, for instance, Hogarth, 1976; Clutton‐Brock, 1991; Avise, 2013). Actually, prominent increases in embryo size during incubation were recorded in a number of invertebrates and invertebrate chordates in the late 19th and early 20th centuries. Nutritional roles were ascribed to some temporary structures (termed placentas, placental or trophic/nutritive membranes or pseudoplacentas) developing around and/or by embryos, and modes and sources of nutrition for embryos were suggested in sponges (Dendy, 1888; Gatenby, 1920), turbellarians (Bresslau, 1904), digeneans (Lynch, 1933), molluscs (Leydig, 1855; Stepanoff, 1865; Ziegler, 1885; Poyarkoff, 1910; Gilmore, 1917), polychaetes (Goodrich, 1900), bryozoans (Braem, 1890, 1897; Harmer, 1902, 1926), kamptozoans (Nickerson, 1901), crustaceans (Weismann, 1877), onychophorans (Sedgwick, 1885; Sclater, 1888), insects (Heymons, 1912; Hardenberg, 1929), nematodes (Maupas, 1900), echinoderms (Mortensen, 1894, 1920; Clark, 1898, 1901; Vaney, 1925) and salps (Huxley, 1851; Brooks, 1893) (see online Appendix S1 for additional references). Most of these reports were overlooked or forgotten; two rare exceptions published later are the monographs of Hagan (1951) and Manton (1949) on insects and Onychophora. Their work, and some early information on salps, is commonly mentioned in textbooks.

**Figure 1:**
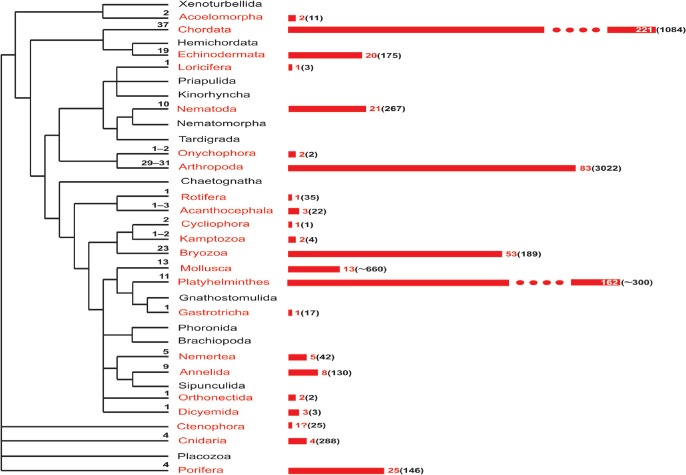
Distribution and inferred origins of matrotrophy across the animal kingdom. In each phylum, numbers on the dendogram (left) show the conservatively estimated number of independent origins of extraembryonic nutrition (EEN). Numbers on the bars (right) and bar lengths reflect the number of families that are either wholly matrotrophic or include species with EEN. Numbers in parentheses show the approximate number of families within phyla [based on the World Register of Marine Species, Animal Biodiversity (Zootaxa) database, and several other databases (such as World Porifera database, www.bryozoa.net, www.onychophora.com, etc.; some numbers were obtained from experts)] including/consisting of matrotrophic species. The scale is truncated for Chordata and Platyhelminthes. The cladogram is based on Dunn et al. (2008, 2014), Hejnol et al. (2009), Edgecombe et al. (2011) and Philippe et al. (2011).

Our analysis, based on an extensive literature compilation and our own research studies, reveals that matrotrophy is recorded or inferred (based on indirect evidence) in more than half of all animal phyla (at least 21 of 34), many with placenta‐like structures (Fig. 1, Table 1, see online Appendix S1). Ten phyla are represented only by a few or several matrotrophic species, whereas in 11 others EEN is either pervasive or widespread, or at least not uncommon. Here, we attempt to integrate patterns across Animalia, focusing on four aspects of invertebrate matrotrophy among and within phyla: (*i*) distribution of the morphological sites of EEN, (*ii*) distribution of matrotrophic modes and mechanisms, (*iii*) indications of broad trends in the evolution of structural complexity of nutritional organs, and (*iv*) independent evolutionary origins of matrotrophy. Our results deliver a significantly revised portrait of the occurrence of matrotrophy and placentation that should help to guide further studies of their phylogenetic, genetic, and developmental origins, constraints, and adaptive significance.

**Table 1:** Distribution of matrotrophy within higher taxa of Animalia. Phyla and classes in left column are arranged in order of decreasing numbers of families that include matrotrophic species. Wholly matrotrophic groups are shown in red. *, taxon with underestimated number of matrotrophic species, see discussion in text. Data on invertebrates and salpids summarized here are based on Appendix S1

Phylum Class/order	Number of matrotrophic species	Number of families (entirely matrotrophic or including matrotrophic species)	Site of matrotrophicincubation (viviparity *versus* brooding)
**Chordata**			
Mammalia	5750	157	Viviparous, oviparous
Chondrichthyes	351*	24*	Viviparous
Osteichthyes	110	15	Viviparous, 4 brooders, guarding
Reptilia	*	13*	Viviparous
Amphibia	38	9	Viviparous, 3 brooders
Ascidiacea	6	2	Brooding, 1 viviparous sp.
Thaliacea/Salpida	48	1	Viviparity followed by brooding
	>6000	∼**221**	Predominantly viviparous
**Platyhelminthes**			
Digenea	∼18000	∼150	Viviparous
Cestoda	17	8	Viviparous
Monogenea	∼450	2	Viviparous
‘Turbellaria’	8	2	Viviparous
	Total:∼18475	**∼162**	Viviparous
**Arthropoda**			
Arachnida/Pseudoscorpionida	3385	25	Brooding
Arachnida/Scorpionida	1753	14	Viviparous
Arachnida/Acari	2	2	Viviparous
Insecta/Diptera	∼809	9	Viviparous
Insecta/Strepsiptera	∼600	8	Viviparous
Insecta/Dermaptera	13	3	Viviparous
Insecta/Coleoptera	15	3	Viviparous
Insecta/Hemiptera	∼5033	2	Viviparous
Insecta/Psocoptera	4	1	Viviparous
Insecta/Blattoidea	1	1	Viviparous
Crustacea/Isopoda	13	9	Brooding, 2 viviparous spp.
Crustacea/Gymnomera	37	3	Brooding
Crustacea/Anomopoda	19	1	Brooding
Crustacea/Ctenopoda	1	1	Brooding
Crustacea/Decapoda	1	1	Brooding
	Total:∼11686	**83**	Viviparous *versus* brooding ∼1/1
**Bryozoa**			
Stenolaemata/Cyclostomata	626	23	Viviparous
Gymnolaemata/Cheilostomata	∼122	18	Brooding, 5 viviparous spp.
Gymnolaemata/Ctenostomata	9	7	Brooding
Phylactolaemata	87	6	Brooding
	Total:∼844	**53**	¾ are viviparous, ¼ are brooding
Porifera*			
Demospongiae	24	17	Viviparous
Calcarea	6	6	Viviparous (shift to brooding in 2 spp.)
Homoscleromorpha	3	1	Viviparous
Hexactinellida	1	1	Viviparous
	Total: 34	**25**	Predominantly viviparous
Nematoda*			
Chromadorea	33	18	Viviparous
Enoplea	3	3	Viviparous
	Total: 36	**21**	Viviparous
**Echinodermata**			
Holothurioidea	32	7	14 viviparous, 18 brooding spp.
Asteroidea	10	5	6 viviparous, 4 brooding spp.
Ophiuroidea	8	3	1 viviparous, 7 brooding spp.
Echinoidea	7	3	Brooding
Crinoidea	2	2	1 viviparous, 1 brooding spp.
	Total: 59	**20**	22 viviparous, 37 brooding spp.
**Mollusca**			
Gastropoda	23	8	18 viviparous, 5 brooding spp.
Bivalvia*	42	5	Brooding
	Total: 65	**13**	Predominantly brooding
**Annelida**			
Polychaeta	19	7	Viviparous
Clitellata	3	1	Brooding
	Total: 22	**8**	Predominantly viviparous
**Nemertea**	14	**5**	Viviparous
**Cnidaria**			
Scyphozoa	2	2	1 viviparous, 1 brooding spp.
Hydrozoa	1	1	Viviparous
Anthozoa	2	1	2 viviparous
	Total: 5	**4**	4 viviparous, 1 brooding spp.
**Dicyemida**	∼107	**3**	Viviparous
**Acanthocephala**			
Eoacanthocephala	3	1	Viviparous
Palaeacanthocephala	1	1	Viviparous
Archiacanthocephala	1	1	Viviparous
	Total: 5	**3**	Viviparous
**Onychophora**	86	**2**	Viviparous
**Orthonectida**	24	**2**	Viviparous
**Kamptozoa**	5	**2**	Brooding
**Acoelomorpha**	2	**2**	Viviparous
**Cycliophora**	2	**1**	Brooding & viviparous
**Rotifera**			
Monogononta	1	**1**	Viviparous
**Gastrotricha**	1	**1**	Viviparous
**Loricifera**	1	**1**	Viviparous

## II. MAKING SENSE OF TERMINOLOGY

The term ‘matrotrophy’ was coined by Wourms (1981, p. 473), who classified reproductive patterns in fishes as ‘either lecithotrophic, i.e. exclusively yolk dependent, or matrotrophic, i.e. in receipt of a *continuous supply of maternal nutrients during gestation*’ [our italics]. The term was thus restricted to provisioning of an embryo.

Etymologically, matrotrophy (feeding by a mother) suggests a wider operational application. Following Blackburn (1992, 2000, 2014), this term could be applied to any type of maternal or paternal nutrient provisioning lasting until the stage when the offspring (embryo and post‐embryo) attains nutritional independence (fends for itself), i.e. not only pre‐paritive (prenatal) parental feeding, but also post‐paritive and post‐gestational, including matrophagy (consumption of the mother's tissues) in some arthropods, lactation in mammals, feeding by transformed parental epithelia (dermaphagy) in some fishes and amphibians (also considered as matrophagy), crop milk in some birds, ‘royal jelly’ in honey bees, and nutrition by any type of food collected and prepared by a parent for consumption by its young (including post‐paritive feeding in some insects, many birds and most mammals during the post‐lactation period). Matrotrophy *sensu lato* can thus apply to all early developmental stages—embryos, larvae and juveniles. The term ‘extraembryonic nutrition’ should strictly refer to the earliest stage of development, in contradistinction to ‘postembryonic nutrition’, though we traditionally (conventionally) use EEN for both these cases in our paper. The term ‘fetal nutrition’ (Wourms, 1977), used predominantly for vertebrates, would be a compromise.

Pre‐ and post‐paritive nutrient provisioning can be direct and indirect. Direct provisioning refers to nourishment provided continuously from the parent to the young during part of or the entire duration of incubation and/or guarding. Indirect provisioning denotes that the entire amount of extra‐vitelline nutrient required for the development of any particular offspring is supplied only once by the parent—even in the case of incubation, the parent is not subsequently involved in providing nourishment. For example, some gastropod molluscs, polychaetes and free‐living flatworms supply the developing offspring with nutritive eggs or albumen in free‐laid or incubated egg‐capsules (sibling cannibalism can also occur in some cases). Some insects collect paralysed prey (or deposit egg[s] inside the host animal) or fresh or decomposed plant materials for this purpose.

Matrotrophy *sensu stricto* can be defined as continuous (i.e. direct), parental, extra‐vitelline nutrient supply during gestation (incubation of the young), whether viviparous or brooding (and, thus, pre‐ and post‐paritive). In most cases it is pre‐paritive and associated with viviparity. We delineate *viviparity* as an incubational mode, with embryonic development occuring within the reproductive system (ovary or sexual duct), body cavity (coelom, pseudocoel or haemocoel) or parental tissues or tissue‐like layers (parenchyma, mesohyl, mesoglea), resulting in live birth. During *brooding*, progeny are released as zygotes, embryos or post‐embryos but are incubated on the parental body surface, inside its infoldings (including mantle and atrial cavities) or invaginations (either non‐specialized or transformed as brood chambers) or in the gastric system (mouth, stomach and its outpocketings). Thus, in viviparity, the development of young is pre‐paritive, whereas in brooding it is post‐paritive. In both instances, not only embryonic, but also postembryonic stages can be incubated, and either extra‐ or postembryonic nourishment (or both) can occur.

Since the term ‘embryo’ strictly applies to the pre‐paritive/prehatched developmental phase, and not later stages that may be immature and dependent on matrotrophy for survival, we likewise use the inclusive terms ‘young’ and ‘offspring’ to refer to embryonic and later stages (for analysis of terminology applied to vertebrates, see Blackburn, 2014). Regardless of the incubation site, the incubation period can be considered as ‘gestation’. The term ‘pregnancy’ is more commonly used for viviparous vertebrates (but see Avise, 2013). In the case of brooding, the term ‘larval/juvenile release’ (instead of ‘birth’) is preferable for describing the moment when the offspring leaves the parent.

The situation in marsupial mammals is instructive in this respect. Whereas prenatal development occurs *in utero*, being supported by placentation, the post‐paritive period continues in the marsupium and is accompanied by lactation. Thus, during gestation viviparity is followed by brooding in this case, and both incubational modes are matrotrophic.

On these views, viviparity and brooding are not synonymous. Yet the terms are often confused in describing internal incubation prior to the expulsion of live young from the parent body. Examples of such descriptions are ‘the site of brooding in viviparous forms’ (Hendler, 1975, p. 692, in Ophiuroidea), or a ‘brooding nemertine’ with intra‐ovarian incubation (Norenburg, 1986, p. 275). In this paper we also intentionally avoid the confusing term ‘ovoviviparous’ (for discussion see Wourms, 1981; 1992, 1994a, 2000, 2014; Frick, 1998).

It also should be mentioned that matrotrophy is often characterized as a ‘post‐fertilization’ event in vertebrates (Blackburn, 2014; Pollux *et al*., 2014). However, because parthenogenesis is widespread in invertebrates, including matrotrophic taxa (for example, aphid insects, digenean parthenitae, and the only known viviparous gastrotrich), we exclude ‘post‐fertilization’ from our definition.

We distinguish five matrotrophic modes (‘patterns of matrotrophy’ in Blackburn, 2014): (*i*) oophagy, ingestion of sibling ova or products of their resorption; (*ii*) embryophagy (=adelphophagy), sibling cannibalism; (*iii*) histotrophy, absorption (and sometimes phagocytosis, see Section IV.5) of nutrients directly from the surrounding fluid of the parental body cavity, incubation chamber or tissues by the offspring external cell layer; (*iv*) histophagy, ingestion of secretions from parental tissues or glands, feeding on floating cells and cell debris, or eating maternal tissues or organs, most often epithelium (sometimes hypertrophied) of parental sexual ducts, skin or brood chamber, but also the entire uterus, fat body, intestine, etc. (this last variant is termed ‘matrophagy’, and considered as a separate mode by Blackburn, 2014); and (*v*) placentotrophy, EEN involving any form of placenta, defined as ‘any intimate apposition or fusion of the fetal organs to the maternal tissues for physiological exchange’ (Mossman, 1937, p. 156; see also Wourms, 1981; Blackburn, Evans & Vitt, 1985; 1992, 1999a, 2000, 2014).

Schindler & de Vries (1988) described ovarian matrotrophy in teleost fishes as aplacental, despite the apposition of embryonic and ovarian epithelia (but lacking specialized nutritional structures). ‘Aplacental’ may be better applied to all types of EEN lacking contact/apposition of parental and fetal tissues. Because many researchers have considered a ‘placenta’ applicable only to eutherian mammals, alternatives such as ‘pseudoplacenta’ (Roonwal, 1939, cited by Hagan, 1951) and ‘placental analogue’ (Wourms, 1977, p. 381; Blackburn, 1999*c*; 2013a, 2013b) have often been used for nutritive structures in other vertebrates and in invertebrates. The reasoning is that the allantoic mammalian placenta consists of specialized maternal and fetal interdigitating tissues forming a complex organ. In most instances, however, placentation is structurally much simpler, and specialized nutritive tissues/organs may or may not be present. Following Mossman (1937) we interpret the close apposition between epithelia/tissues of parent and offspring, with nutrient transport, as sufficient to describe such contact as placental. Thus, here we define a ‘placental analogue’ as any local zone of enhanced nutritional transport, whether simple apposition of non‐specialized epithelia or specialized parental–embryonic tissue/cell complexes, as well as nutritive structures formed exclusively by the parent or the embryo and increasing the entire secreting and absorbing surfaces as well as the contact surface area between them.

## III. MATERIALS AND METHODS

There is some uncertainty among authors regarding the number of the currently recognized metazoan phyla (Dunn *et al*., 2008, 2014; Hejnol *et al*., 2009; Edgecombe *et al*., 2011). Here, we accept 34 phyla, including Xenoturbellida, Acoelomorpha, Acanthocephala and Rotifera as separate entities.

Our analysis is based on a combination of original research (Bryozoa), personal communications from taxonomic experts (20 other phyla) and data from the literature (all phyla) that demonstrate or strongly infer instances of matrotrophy. Direct evidence of nutrient transport from a parent to developing offspring explicitly included oophagy, embryophagy, histophagy, experimental *in situ* transfer of metabolites with radiolabelled markers, increase in dry mass of the fully developed embryo/post‐embryo over that of the ovulated egg, and ultrastructural evidence of exo‐ and endocytosis. Indirect evidence was taken to include the appearance of temporary nutrient transfer structures (apparent or inferred) during the incubation period in the parent, embryo or both, histochemical data on the content of the parental tissues/cells during gestation, increase in embryo size (linear or volumetric, including experimentally induced), mass loss and destruction of the parent's tissues when ‘sacrificed’ for nourishment, the mating of progeny inside the parent, and certain other characters in a few difficult cases (e.g. changes in size, shape and distributional pattern of yolk granules in the early embryo in comparison with the ovulated egg; see Ostrovsky, 2013*b*).

Closer consideration exposes constraints in applying some of these evidential criteria to invertebrates. Chemical composition and dry mass as used in some vertebrate studies (reviewed in 1994b, 2014) are seldom used in invertebrate research, since eggs and embryos are often quite small. Examples include two onychophorans, two insects, two isopod crustaceans, and several echinoderms (Pandian, 1972; Stay & Coop, 1973; Denlinger & Ma, 1974; Lawlor, 1976; Turner & Rutherford, 1976; Turner & Dearborn, 1979; Lawrence, McClintock & Guille, 1984; Schatt, 1988; Havel, Wilson & Hebert, 1989; de Eguileor *et al*., 1994; Frick, 1998; Bosch & Slattery, 1999; Sunnucks *et al*., 2000). Also relatively rare are experiments with radiolabelling and diet manipulation (Burton, 1962; Nollen, 1968; King & Lumsden, 1969; Blackman, 1974; Gilbert, 1974; Gremigni & Domenici, 1976; Calloway, 1982; Tompa, 1984; Toolson, 1985; Silverman, Kays & Dietz, 1987; Hoese & Janssen, 1989; Frick, 1998; McIntyre *et al*., 2009) and ultrastructural studies (Rogers, Ellis & Denham, 1976; Domenici & Gremigni, 1977; Ellis *et al*., 1978; Walker & Campiglia, 1988; Cable & Tinsley, 1991; Campiglia & Walker, 1995; Schwartz & Dimock, 2001; Korneva, 2005; Sewell *et al*., 2006; Moosbrugger *et al*., 2012; Korneva *et al*., 2014), which is why, in addition to the prominent increase in embryo size (linear, volumetric, or both), many authors have used specialized temporary structures in both parent and offspring during incubation as evidence of EEN (e.g. Hagan, 1948, 1951; Mukai, Terakado & Reed, 1997; Farley, 2001; see also discussion for vertebrates in Blackburn, 2014). It also should be mentioned that developing embryos of aquatic invertebrates may increase in volume (and wet mass) owing to water uptake, regardless of whether or not matrotrophy is present (discussed in 2013a, 2013b). Taking these reservations into account, we selected those examples from the literature and our own data where cumulative evidence (dimensional, developmental, morphological and cytological) strongly pointed to the presence of EEN regardless of the degree of matrotrophic input.

We concur with Blackburn's (2014, p. 3) view, that ‘Matrotrophy and lecithotrophy represent extremes of a continuum’. Embryos in many species rely on both yolk and EEN (see also Blackburn, 1993; Dulvy, 1998; Lombardi, 1998). In the vertebrate literature, the term ‘substantial matrotrophy’ is used when extra‐vitelline sources account for most of the nutrients during development. The contrary balance, with predominantly lecithotrophic and restricted matrotrophic provisioning, is frequently termed ‘incipient matrotrophy’ (Blackburn, 1992, 2014). Most or all viviparous vertebrates have at least some degree of EEN, and many of them are predominantly lecithotrophic (D. G. Blackburn, personal communication 2014). This continuum is also characteristic of invertebrates. Yet only recently have researchers working on invertebrates attempted to differentiate species with varying degrees of matrotrophy *versus* lecithotrophy using dimensional (embryonic increase in volume), morphological (degree of hypertrophy of nutritive cells) and cytological (oocyte type) criteria (e.g. Ostrovsky, Gordon & Lidgard, 2009; 2013a, 2013b).

We extracted data on invertebrate matrotrophy from more than 580 published papers and monographs (see online Appendix S1). A large data matrix was compiled for matrotrophic species from more than 200 invertebrate families. It includes taxon names, egg *versus* larval/juvenile size and embryonic size increase (where known), site of incubation, parental as well as embryonic structures involved in nutrient transfer (when inferred/described), and corresponding references. We also compiled comparative data on the distribution of matrotrophy in vertebrates. For certain taxa, extrapolations were made based on the general uniformity of the incubation method. For example, we regarded as matrotrophic the entire group of parasitic digenean flatworms, based on consistency among studies that show embryos invariably grow while floating in the pseudocoel fluid of the parental parthenogenetic generations.

To estimate the number of independent origins of matrotrophy among and within invertebrate phyla we analysed (*i*) the taxonomic distribution of the major reproductive patterns (oviparity *versus* non‐matrotrophic and matrotrophic incubation) using recently published molecular phylogenies, and compared distribution patterns of (*ii*) incubation sites and (*iii*) matrotrophic modes. Since data are lacking for many invertebrate groups, our view is that these estimates should be considered exceptionally conservative. We also considered the possible reasons why matrotrophy is absent in some phyla.

## IV. RESULTS AND DISCUSSION

### (1) Distribution of matrotrophy across Animalia

Matrotrophy is established or inferred in 20 of 33 invertebrate phyla, but its occurrence within a phylum varies greatly (Fig. 1). To facilitate analysis, we cluster the invertebrate phyla with EEN into three groups using a somewhat arbitrary criterion: those with 1–5 matrotrophic species; those in which EEN is more widespread (more than 10 but not much more than 50 species); and those in which matrotrophy is extensive (from one hundred to thousands of species) or universal (regardless of phylum size) (Table 1; see online Appendix S1 for taxa and references).

The first group comprises seven phyla. Rotifera, Gastrotricha and Loricifera have only a single known matrotrophic species each. There may also be a matrotrophic species in Ctenophora. Acoelomorpha contains 2 species with EEN (from 2 families). Five matrotrophic species have been recorded in 3 phyla: Acanthocephala (from 3 families in 3 classes), Kamptozoa (2 families in 2 orders) and Cnidaria (2 scyphozoan families, a hydrozoan family and an anthozoan family).

The second group comprises 6 phyla. Nemertea includes 14 known matrotrophic species (from 5 families) and Annelida has 22 species (7 families of Polychaeta, 1 family of Clitellata). In Porifera, matrotrophy is suggested in at least 34 species (25 families) from all 4 classes: Calcarea (6 species, 6 families), Demospongiae (24 species, 17 families), Homoscleromorpha (3 species, 1 family) and Hexactinellida (1 species). In Nematoda, matrotrophy is indicated in 36 species, in classes Enoplea (3 species, 3 families) and Chromadorea (33 species, 18 families). In Echinodermata, there are 59 species (20 families) with EEN recorded/inferred across all 5 extant classes: Ophiuroidea (8 species, 3 families), Asteroidea (10 species, 5 families), Holothuroidea (32 species, 7 families), Echinoidea (7 species, 3 families) and Crinoidea (2 species, 2 families). Matrotrophy is recorded or inferred in 65 species (13 families) of Mollusca—at least 42 species (5 families) of bivalves and 23 species (8 families) of gastropods. It is likely that the numbers of matrotrophic sponges, nematodes and bivalve molluscs are underestimated. For instance, all species in such bivalve genera as *Musculium*, *Pisidium* and *Sphaerium* for which reproduction has been studied show signs of EEN, thus making it very probable that these taxa are entirely matrotrophic.

The third group also comprises 7 phyla. Platyhelminthes leads with approximately 18475 matrotrophic species: 8 turbellarians (2 neorhabdocoel families), 17 Cestoda (8 families), ∼450 Monogenea (2 families) and all ∼18000 species of Digenea (∼150 families). Arthropoda contains the second‐largest matrotrophic representation, with ∼11686 species (83 families). In class Arachnida, all Scorpionida and Pseudoscorpionida (∼5138 species, 39 families) have extraembryonic nutrition, and there are 2 matrotrophic species (2 families) of mites. Among insects EEN is present in all Strepsiptera (∼600 species, 8 families), more than 800 species of Diptera (9 families) and more than 5030 species of Hemiptera (2 families), plus 33 species from 8 families in 4 other orders (Dermaptera, Blattoidea, Psocoptera and Coleoptera). Crustacean taxa with matrotrophy include all Gymnomera (37 species, 3 families), 19 species (1 family) of Anomopoda and a ctenopod species (class Branchiopoda) as well as 13 species (9 families) of Isopoda and 1 species of Decapoda (class Malacostraca). Similar to the above example, the number of matrotrophs—parasitic flatworms and insects—is clearly underestimated.

In the phylum Onychophora, there is evidence for EEN in 86 species (in both families). This number may actually approach 100 species, but the lack of data from genera that include matrotrophs prevents more precise estimation. There are 3 small, wholly matrotrophic phyla—Dicyemida (107 species, 3 families), Orthonectida (24 species, 2 families) and Cycliophora (2 species, 1 family).

Finally, the wholly colonial lophotrochozoan phylum Bryozoa ranks third among invertebrates for matrotrophy. Workers have only recently discovered the wide extent of matrotrophy in this phylum (Reed, 1991; Levin & Bridges, 1995; Ostrovsky *et al*., 2009). Updating our previous estimate, at least 844 species in 53 families of bryozoans are matrotrophs, and more occurrences are likely as our anatomical and ultrastructural studies to date cover only 30% of the ∼180 gymnolaemate families (2013a, 2013b, suggested >1000 matrotrophic species). Moreover, compared with all aquatic invertebrates, bryozoans have the widest within‐phylum taxonomic distribution of placental analogues, unusually diverse incubational structures, and numerous instances of incipient matrotrophy (2009, 2013a, 2013b; Ostrovsky *et al*., 2009).

A comparison with Chordata provides a context for the total estimate of invertebrate species with EEN (Table 1). Among urochordates, matrotrophy occurs in Thaliacea (all 48 species of Salpida) and Ascidiacea (6 species, 2 families). Mammals, including monotremes (altogether 5750 species in 157 families; Wilson & Reeder, 2011), are all matrotrophs (Blackburn, 2005). Estimating numbers of matrotrophic species among fishes, amphibians and reptiles is difficult due to insufficient data on reproduction for many species. According to Lombardi (1998) there are 513 matrotrophic species of sharks and rays belonging to 40 families, whereas the estimate of Dulvy (1998) is more modest; we counted 351 species in 24 families in his list (see also Dulvy & Reynolds, 1997). Based on egg size, embryonic linear/volume/mass increase, development of trophic structures as well as egg/sibling consumption by developing juveniles, matrotrophy was recorded/inferred in 15 families of Osteichthyes. Thirteen families include viviparous species, whereas the Syngnathidae includes ‘patrotrophic’ brooders, and the discus fishes (*Symphysodon*) are guarders (Wourms, 1981; Trexler, 1985; Blüm, 1986; Wourms *et al*., 1988; Schindler & Hamlett, 1993; Lombardi, 1996, 1998; Carcupino *et al*., 2002; Reznick, Meredith & Collette, 2007; Pollux *et al*., 2009; Marsh‐Matthews, Deaton & Brooks, 2010; Pires, Arendt & Reznick, 2010; Marsh‐Matthews, 2011; Pires *et al*., 2011; Blackburn, 2014, and references therein). In total, EEN was recorded/inferred in at least 110 teleost species. Modes of matrotrophy recorded in fishes include oophagy, embryophagy, histotrophy, histophagy and placentotrophy.

The general picture of matrotrophic distribution in Amphibia is far less complete. EEN was recorded/inferred in 38 species of 9 families. *Rhinoderma* and two skin‐feeding caeciliids are brooders whereas the other matrotrophic forms are viviparous (Wake, 1977, 1980, 1982, 1993; Blüm, 1986; Goicoechea, Garrido & Jorquera, 1986; Greven, 1998; Lombardi, 1998; Wake & Dickie, 1998; Jared, Navas & Toledo, 1999; Dopazo & Korenblum, 2000; Kupfer *et al*., 2006; Buckley *et al*., 2007; Gower *et al*., 2008; Wilkinson *et al*., 2008, 2011; Blackburn, 2014, and references therein). All matrotrophic modes have been recorded in amphibians except for placentotrophy, although one species may utilize it.

Among reptiles, matrotrophy occurs only by placentation and has been documented only among squamates. The presence of a placenta, however, does not necessarily imply substantial matrotrophy, since this organ ancestrally functions in gas exchange and provision of calcium, sodium, and small amounts of organic nutrients (Blackburn, 1992; Thompson & Speake, 2006; Stewart, 2013). Most viviparous squamates for which information on placentas is available are chiefly lecithotrophic with incipient matrotrophy (Stewart, 1992; 1999b, 2014; Villagrán, Méndez de la Cruz & Stewart, 2005). Even in these, ultrastructural evidence of cellular specializations for nutrient transfer has been shown in a number of lizards and snakes (reviewed in Blackburn, 2014). Recently Blackburn (2014) suggested that incipient placentotrophy is universal among viviparous squamates (while stressing that only species with substantial nutrient provisioning are classified as matrotrophic in the vertebrate literature; D.G. Blackburn, personal communication 2014). Since matrotrophy is correlated with viviparity, which occurs in about 20% of squamates (1999c, 2014), all these species can be considered as having EEN. Based on Pincheira‐Donoso *et al*. (2013) there are 9193 squamate species; about 1800 species may thus be matrotrophic. Morphological and experimental evidence on placentation has been recorded for species in 13 squamate families (Weekes, 1935; Bauchot, 1965; Blackburn, Vitt & Beuchat, 1984; 1985, 1993, 1994b, 1998, 1999b, 2005, 2014; Blackburn *et al*., 1985; Blüm, 1986; Stewart & Blackburn, 1988; Stewart, 1992, 1993, 2013; Lombardi, 1998; Stewart & Thompson, 1998, 2000, 2009; Thompson, Stewart & Speake, 2000; Blackburn & Vitt, 2002; Jerez & Ramírez‐Pinilla, 2003; Villagrán *et al*., 2005; Ramírez‐Pinilla, 2006; Thompson & Speake, 2006; Vieira, de Perez & Ramírez‐Pinilla, 2007; Leal & Ramírez‐Pinilla, 2008; Blackburn & Flemming, 2009; Stewart & Ecay, 2010, and references therein). Among these, substantial placentotrophy evolved in all six subclades of a single lizard family, Scincidae (Blackburn, 2014).

Because of these uncertainties, we caution that for Chordata, our estimates of the number of matrotrophic species and families should be considered only as preliminary ones: above 6000 species (reptiles excepted) and 220 families.

### (2) Brief overview of matrotrophy in invertebrates

Patterns of invertebrate matrotrophic reproduction are extraordinarily diverse with respect to sites, modes, mechanisms and structures providing extraembryonic nutrition. Each of these aspects is analysed on a comparative basis in the sections that follow. Before presenting this analysis we give brief, phylum‐by‐phylum descriptions of EEN, focusing on typical examples and exceptions. The full range of taxonomic and structural diversity of matrotrophic adaptations (including in invertebrate chordates) is described and references are given in Appendix S1. Superscript numbers in Appendix S1 identify papers that provide histochemical and/or ultrastructural and experimental evidence for matrotrophy (e.g. autoradiographic labelling, calcium transfer, diet manipulation, dry mass and organic mass analysis, estimation of energetic content). Some potentially matrotrophic species are also included.

#### (a) Non‐Bilateria and Acoelomorpha

The vast majority of Porifera are larviparous, releasing young as larvae. Their embryos are incubated in mesohyl, surrounded by a specialized cellular capsule (sometimes termed a ‘follicle’ or ‘epilarval trophocyte epithelium’) of varied origin. Matrotrophy is suggested in more than 30 species from all 4 classes based on a variety of evidence: dimensional (prominent embryonic increase in size), developmental (macromere enlargement, migration of maternal cells to the embryonic cavity and their degeneration and phagocytosis) and ultrastructural (presence of the same type of inclusions in contacting larval and maternal cells).

In contrast to sponges, most Cnidaria are oviparous, releasing young as eggs. Embryonic incubation is known in some Anthozoa, Scyphozoa and Hydrozoa. A marked increase in embryo size occurs during intraovarian incubation in the scyphozoan *Chrysaora hysoscella*. In this species larvae develop inside the ovary. In *Stygiomedusa gigantea*, the asexually developed “larvae” transform into scyphistomas that grow inside special protrusions of the stomach wall, also surrounded by a special capsule (‘chorion’). In the hydrozoan medusa *Crossota millsae*, early embryonic development occurs in the ovary, whereas growing juvenile medusae burst out of it and are suspended beneath the maternal subumbrella for some time. EEN is also suggested in two *Acropora* corals in which larvae develop within an envelope of mesoglea and gastrodermis. As the embryos increase in size, they fill the coelenteron of the parent, with mesenteries firmly adhering to mesenterial envelopes surrounding the large planulae.

Substantial embryonic enlargement occurs in the platyctenean ctenophore *Lyrocteis imperatoris*. In this species the growing larvae develop inside an expansion of the ovarian diverticulum, but more evidence is required to confirm matrotrophy.

Finally, phylum Acoelomorpha contains two species in which embryo enlargement occurs in a so‐called “embryonic vesicle” inside the parenchyma.

#### (b) Lophotrochozoa

Among viviparous Platyhelminthes, embryonic development occurs predominantly in the uterus. Ultrastructural and experimental evidence has shown transfer of parentally derived substances to the embryos in a number of turbellarians, monogeneans, cestodes and digeneans. In gyrodactylid monogeneans, two daughter generations are enclosed inside one another, and both form inside the parent as in Russian dolls. Nutrient transfer occurs across each of the series of interfaces between the parent and older embryo in its uterus, and the older embryo with the younger embryo. In addition to having intrauterine matrotrophy in the sexual generation, parthenogenetic generations of Digenea nourish their progeny in the body cavity (pseudocoel). In turbellarians of the genus *Paravortex*, embryos develop inside the parenchyma. It is suggested that the transfer of soluble and particulate nutrients from the parental gut occurs *via* the wall of the embryonic capsule. Oophagy is also suggested in one turbellarian species.

In the gastrotrich *Urodasys viviparus*, one very large embryo (half the size of the adult) grows *in utero*; a similar situation is also recorded in the rotifer *Asplanchna sieboldi*, in which embryonic enlargement was induced experimentally. Embryonic enlargement occurs during incubation in some Acanthocephala and Kamptozoa, in which progeny develop in the pseudocoel and brood pouch, respectively. In several viviparous Nemertea, juveniles increase in size while developing inside either the ovary or gonoduct. Relatively little is known about the nutritive modes in these cases, but histotrophy (Acanthocephala, Nemertea), histophagy (Kamptozoa) and placentotrophy (Gastrotricha, Rotifera and Kamptozoa) can be inferred cautiously.

In some viviparous polychaete annelids, embryonic growth occurs in the main coelom or coelomic pouches, resulting in the formation of segmented setigerous larvae. While nutritive mechanisms are unclear, both histotrophy and histophagy are probably involved. In leeches, juveniles are incubated either inside the brood pouch (*Marsupiobdella africana*) or directly on the parental body surface (*Glossiphonia complanata, Helobdella stagnalis*). Nutrient transfer across epithelia of the parent's ventral side and juvenile posterior sucker has been shown experimentally in *Glossiphonia* and suggested by histochemical data in *Helobdella*.

Extraembryonic nutrition has been demonstrated or inferred in a variety of incubating bivalve and gastropod Mollusca. In brooding Bivalvia, embryos develop in the gills, sometimes surrounded by special brood sacs that are outgrowths of the gill filaments. Transfer of substances to the growing progeny has been demonstrated by both ultrastructural and experimental studies in a few species. Matrotrophic gastropods incubate their growing young either inside a subhaemocoelic brood pouch, in the oviduct, or *in utero*. Oophagy as well as histotrophy are suggested in different cases. In some species, a round sac (podocyst) develops around the embryo, presumably acting as a placenta. Massive transport of calcium from the parent to the podocyst has been shown experimentally in one species.

The vast majority of species in phylum Bryozoa incubate their young. Placentotrophy is suggested for the entire class Phylactolaemata and extant species of class Stenolaemata (order Cyclostomata), which exhibit brooding and intracoelomic viviparity, respectively. Among cyclostomes, EEN supports polyembryony—the production of multiple embryos from a single small egg inside an expansive incubatory gonozooid. Both matrotrophic brooding and viviparity are known in class Gymnolaemata, which is characterized by a wide structural diversity of incubatory chambers and varying degrees of embryonic enlargement and placental development.

In the unique, complex cycliophoran life cycle, EEN occurs in different generations in the course of asexual and sexual reproduction. In the former instance, embryos grow inside a feeding stage within the fluid‐filled cavity of the brood chamber. During sexual reproduction, a chordoid larva develops inside a female, surrounded and nourished by its degenerating tissues.

The phyla Dicyemida and Orthonectida provide two exceptional matrotrophic examples. In the former, embryogenesis occurs intracellularly inside the axial cell of nematogen and rhombogen stages of the complex life cycle. In orthonectids, development of the sexual phase is accompanied by prominent embryonic growth inside the plasmodium. The nutritive mechanism is unknown in both cases, but diffusion and active transmembrane transport are presumably involved.

#### (c) Ecdysozoa

In viviparous Nematoda, larval development occurs in the uterus, and larval growth is extensive in many matrotrophic species. In some instances, development proceeds so far that sexual maturation and copulation occur inside the mother. The internal organs of the pregnant female are used as a food source in at least three species whose larvae continue to grow in the maternal pseudocoel. Oophagy is also inferred for some nematodes.

Arthropods demonstrate the greatest range of any phyla in their incubatory and matrotrophic diversity. Matrotrophic viviparity is obligatory in Scorpionida (*in utero*) and the insect order Strepsiptera (inside the haemocoel), and is moderately frequent or widespread in six other insect orders (in the ovary, uterus or haemocoel). Haemocoelous viviparity accompanied by EEN is also known in two mites. By contrast, all Pseudoscorpionida are matrotrophic brooders that incubate their young inside a ‘silk’ brood sac formed around the sexual opening. Offspring consume nutritive fluid produced by the mother's ovary using an embryonic pumping organ. Among Crustacea, brooders include some branchiopods, most matrotrophic isopods and a decapod, which incubate their progeny inside a marsupium on either dorsal or ventral side of the body. Isopoda also includes two viviparous species that incubate their young in the uterus and show a marked increase in embryo size. Scorpions and some insects possess various placenta‐like structures, and intrauterine ‘milk glands’ evolved in these groups. These glands become fully functional when the mouthparts are formed in the embryo. In arthropods, apart from embryonic increase in linear size and development of specialized structures (of the parent and the embryo), EEN is also evidenced by histological data, dry‐mass increase (in two isopods and two insects) and transfer of radioactive tracers from parent to embryo during gestation (in a scorpion).

About a half of all known Onychophora are matrotrophic, employing incubation in the uterus. In matrotrophic Peripatidae, the major nutritive role is ascribed to the modified uterine wall forming a placental analogue. In this family (with one known exception) the embryo is attached to the uterine wall by a hollow ‘umbilical cord’ or ‘stalk.’ Placenta‐like structures and stalk are absent in the matrotrophic Peripatopsidae, some of which possess a so‐called ‘trophic vesicle’ that is a swollen sac of extraembryonic ectoderm presumably contributing to nutrient uptake.

In the matrotrophic loriciferan *Urnaloricus gadi*, embryonic development occurs in the pseudocoel. Embryos that develop into Higgins larvae reabsorb all the tissue of their maternal stage, the ghost‐larva.

#### (d) Deuterostomia

In Echinodermata, evidence of extraembryonic nutrition is present in all five extant classes. Sites of embryonic incubation vary within and among classes and include ovary/ovotestes, coelom, and a variety of external marsupia. The main evidence for EEN is a substantial increase in embryo size; additional evidence has also been derived from experimental data on dry and organic mass increase and from autoradiographic labelling. Nutritive modes include oo‐, embryo‐ and histophagy, and presumably, histotrophy. For example, transepidermal absorption is inferred to exist in the early stages of development of a holothurian based on autoradiographic experiments.

### (3) Sites of matrotrophy – distribution across phyla

Of the 34 metazoan phyla, only 6 appear to lack any discernable form of embryonic incubation. Sipuncula, Nematomorpha, Tardigrada, Gnathostomulida, Kinorhyncha and Xenoturbellida consist exclusively of egg‐laying/spawning species. In Placozoa, embryo(s) begins development inside the mother (i.e. lecithotrophic viviparity), which later degenerates and releases the embryo (Eitel *et al*., 2011). Five other phyla, while including both oviparous and incubating species (either lecithotrophic–viviparous like Priapulida, or lecithotrophic–brooding like Chaetognatha, Phoronida, Hemichordata, and Brachiopoda), show no evidence of matrotrophy.

Embryonic incubation has been recorded in three species of Ctenophora, the vast majority of which are oviparous. As mentioned above, matrotrophy potentially may occur in one viviparous ctenophore.

The remaining 21 phyla include matrotrophic species, either viviparous, brooding, or both (Table 1). Matrotrophic viviparity is encountered in 20 phyla (not Kamptozoa), whereas matrotrophic brooding occurs in 10, 9 of which (again excluding Kamptozoa) possess both types of incubation—Porifera, Cnidaria, Annelida, Bryozoa, Arthropoda, Echinodermata, Mollusca, Cycliophora and Chordata. While viviparity is recorded widely among families in most of these nine phyla, matrotrophic brooding dominates among Mollusca and Echinodermata. Cycliophora exhibits both types of matrotrophic incubation within a single life cycle. The total number of phyla with viviparity and brooding (matrotrophic or otherwise) are 23 and 15, respectively, with considerable overlap of the two. This pattern highlights the wide distribution of embryonic incubation among Metazoa, with the prevalence of viviparity.

Matrotrophy is associated with all known sites of viviparous incubation, although not in all higher taxa. It occurs in mesohyl (Porifera), mesoglea (two hexacorals), parenchyma (‘turbellarians’, Acoelomorpha and Cycliophora), three types of body cavities – pseudocoel (parthenogenetic generations of Digenea as well as Acanthocephala, a loriciferan and Nematoda with matrophagy), coelom (Polychaeta, some sea stars and holothurians, a few gymnolaemate and all cyclostome Bryozoa) and haemocoel (two Acari and many Insecta including all Strepsiptera), ovary (one scyphozoan and one hydrozoan medusa, majority of matrotrophic Nemertea, numerous insects, a number of echinoderms from four classes except Echinoidea), sexual ducts (matrotrophic Neodermata, Gastropoda, all Scorpionida, numerous Insecta, two isopod crustaceans, all matrotrophic onychophorans, Nematoda, the sole matrotrophic gastrotrich and rotiferan, one nemertean and one ascidian species), and even intracellularly or intraplasmodially (in Dicyemida and Orthonectida). The last instances are exceptional; the most common locations for matrotrophic incubation are the female genital system and body cavities (see online Appendix S1 for details and references here and below).

The same can be said about brooding, almost all known sites of which are associated with matrotrophy, although not in all taxa. Large internal spaces – like the mantle cavity of molluscs – are the usual locations for matrotrophic incubation, along with invaginations of the parental body wall – specialized brood‐chambers (a scyphozoan, Kamptozoa, all phylactolaemate and some gymnolaemate Bryozoa, a leech, Cycliophora and matrotrophic echinoderms from all five classes). In one sea star, progeny are brooded in the stomach. Marsupia made of pre‐existing structures (various appendages and folds), biogenic material (silk‐like protein), or body‐wall outgrowths, are known to form in Crustacea, Pseudoscorpionida, and cheilostome Bryozoa. In two matrotrophic leeches, young are brooded directly on the ventral surface of the mother.

Developmentally timed shifts in the site of matrotrophic incubation are known: (*i*) during viviparous development when the embryo moves from the ovary to the oviduct or uterus (recorded in a gastropod mollusc and a dermapteran insect) and in some teleost fishes (Blackburn, 2014), or from the ovary to haemocoel (in a mite), or uterus to pseudocoel (some nematodes); and (*ii*) transition from matrotrophic viviparity to matrotrophic brooding when juveniles burst out from the gonad, being suspended beneath a maternal subumbrella (hydrozoan medusa *Crossota millsae*), or embryos move from the ovary to the water tubes of the inner demibranchs (suggested in the bivalve *Corbicula fluminea*). In some calcareous sponges, the incubation site changes during incurvation of the larva that moves from the mesohyl to the choanocyte chamber (it is uncertain that EEN is present in both incubational stages). In salps, embryos initially developing in the ovarian follicle are further nourished in an atrial cavity. However, shifts from one matrotrophic brooding site to another are unknown.

Considered as an overall pattern, some taxa show a very restricted range of incubation sites whereas others have different variants. Structural and ecological constraints are reasonably inferred determinants for this aspect of reproduction. Sites of matrotrophic incubation are most diverse in Arthropoda and Bryozoa. Arthropods exhibit viviparity in the ovary (Insecta), genital ducts (Scorpionida, Insecta, Isopoda) or haemocoel (Acari, Insecta) and brooding within ‘silk’ brood chamber (Pseudoscorpionida) and marsupial sac/brood pouch (Branchiopoda, Isopoda, Decapoda). Among Bryozoa, nourishment of the developing young occurs in internal brood sacs (Phylactolaemata, Ctenostomata, Cheilostomata), the introvert (Ctenostomata) and various skeletal brood chambers (Cheilostomata). Viviparous bryozoans (Cyclostomata and Epistomiidae) incubate embryos in the perivisceral coelom, surrounded by either modified peritoneal cells or by ovarian cells, correspondingly. Although matrotrophy is more widespread among chordates and Platyhelminthes, these phyla are notable for the more limited range of incubational variation.

These observations are consistent with a view that matrotrophy evolved repeatedly, irrespective of the site of gestation. What of those groups that incubate young but show no evidence of matrotrophy? In Placozoa, the maternal individual dies soon after the beginning of embryo cleavage and only early developmental stages are incubated. In brooding Phoronida, Pterobranchia (Hemichordata) and Chaetognatha embryos are incubated externally. On the other hand, the absence of parental provisioning in the only known viviparous priapulid (*Meiopriapulus fijiensis*; Higgins & Storch, 1991) and brooding Brachiopoda (James, 1997; Seidel *et al*., 2012) is an enigma; seemingly, the preconditions for evolving matrotrophy are present. Matrotrophy may exist still undiscovered in these two taxa.

### (4) Modes of matrotrophy – distribution among and within phyla

Modes of matrotrophy occurring during embryonic incubation include oophagy, embryophagy, histotrophy, histophagy (including matrophagy) and placentotrophy (see online Appendix S1).

Chordata exhibits all of these modes, with placentotrophy represented most frequently, whether at class or species level – in all Mammalia except monotremes, many squamate reptiles, a relatively large number of bony and cartilaginous fishes, some ascidians, and all salps (Wourms, 1981; Mukai, Saito & Watanabe, 1987; Wourms *et al*., 1988; Godeaux, 1990; Blackburn, 1992, 1993, 2005, 2014; Wourms & Lombardi, 1992; Wooding & Burton, 2008) (Table 2).

**Table 2:** Distribution of the modes of matrotrophy within higher taxa of Animalia. Phyla are arranged in order of decreasing numbers of families that include matrotrophic species. Terminology: oophagy, ingestion of sibling ova or products of their resorption; embryophagy (sibling cannibalism), offspring feed upon developing siblings; histotrophy, the offspring epithelium absorbs nutrients directly from the surrounding fluid of the parental cavity, incubation chamber or tissues; histophagy (including matrophagy*), ingestion of secretions from parental tissues or glands, or feeding on detached cells or hypertrophied epithelium of parental sexual ducts, skin, brood chamber or internal organs; placentotrophy, extraembryonic nutrition involving any form of placenta. Inferred mode: mode of matrotrophy suggested in the literature or by the authors based on indirect evidence

**Taxon**	**Type of matrotrophic nutrition**					
	Oophagy	Embryophagy	Histotrophy	Histophagy	Placentotrophy	Inferred mode
**Chordata**						
Mammalia			+		+	
Reptilia					+	
Amphibia	+	+	+	+*	1 sp. (?)	
Osteichthyes	+	+	+	+	+	
Chondrichthyes	+	+	+	+	+	
Ascidiacea					+	
Thaliacea/Salpida					+	
**Platyhelminthes**						
Digenea			+		+	
Monogenoidea					+	
Cestoda					+	
‘Turbellaria’	+			+	+	
**Arthropoda**						
Arachnida/Pseudoscorpionida				+ (katoikogenic)	+	
Arachnida/Scorpionida			+ (early stages)	+		
Arachnida/Acari						Histotrophy
Insecta		+	+	+*	+	
Crustacea/Branchiopoda					+	Histotrophy
Crustacea/Isopoda		+	+	+	+	
Crustacea/Decapoda						Histotrophy
**Bryozoa**						
Stenolaemata/Cyclostomata					+	
Phylactolaemata					+ (later stages)	Histotrophy
Gymnolaemata			+ (early stages)		+	
**Porifera**					+	Histotrophy
**Nematoda**	+			+*	+	Histotrophy
**Echinodermata**						
Ophiuroidea	+	+				Histotrophy
						Histophagy
Asteroidea	+	+		+		Histotrophy
Holothurioidea	+	+		+		Histotrophy
Echinoidea						Histotrophy
Crinoidea						Oophagy
						Embryophagy
**Mollusca**						
Bivalvia				+	+	Histotrophy
Gastropoda		+		+	+	
**Annelida**						
Polychaeta	+					Histotrophy
						Histophagy
Clitellata					+	Histotrophy
**Nemertea**						Placentotrophy
**Cnidaria**						
Scyphozoa						Histotrophy
Hydrozoa						Placentotrophy
Anthozoa						Placentotrophy
**Dicyemida**						Histotrophy
**Acanthocephala**						Histotrophy
**Onychophora**			+	+ (later stages)	+	
**Orthonectida**						Histotrophy
**Kamptozoa**				+	+	
**Acoelomorpha**						Placentotrophy
**Cycliophora**					+	Histotrophy
**Rotifera**						Placentotrophy
**Gastrotricha**						Placentotrophy
**Loricifera**						Histotrophy

The same range of nutritive modes is found among invertebrates, but histotrophy and placentotrophy predominate (Table 2). Oophagy and embryophagy are less frequent. A ‘turbellarian’ flatworm, a polychaete, two nematodes, an ophiuroid, a holothurian, a sea star and two crinoids comprise known/inferred oophagous forms, whereas a gastropod, a dipteran genus, four isopods, two ophiuroids, several sea stars, two holothurians and a crinoid exhibit (or presumably exhibit) embryophagy (see online Appendix S1 for details and references).

Histophagy occurs in a turbellarian genus and some species of Gastropoda. It is inferred or present in most matrotrophic Polychaeta (18 species), all Pseudoscorpionida (∼3385 species) and katoikogenic scorpions (late in their development), more than 800 species of Diptera, a cockroach, several Isopoda, three Nematoda and seven Echinodermata from three classes. During late developmental stages, histophagy apparently also occurs in matrotrophic Kamptozoa, one teredinid and, possibly, one sphaeriid bivalve and in at least one onychophoran species. Oo‐, embryo‐ and histophagy imply that the young acquire functional mouthparts and pharyngeal muscles as well as certain digestive structures early in development.

In the case of histotrophy, the ‘embryo is suspended in the nutriment’ (Hagan, 1951, p. 231), surrounded by parental fluids without intimate contact with parental cells/tissue. This nutritional mode occurs or is inferred in parthenogenetic generations of Digenea, five known matrotrophic Acanthocephala, all insects with haemocoelous development (including all ∼600 species of Strepsiptera), onychophorans, cheilostome bryozoans, the only known matrotrophic loriciferan, and can be inferred in all Dicyemida, Orthonectida and Cycliophora (during development of dwarf males and chordoid larvae). Histotrophy is also inferred in two matrotrophic mites, a decapod crustacean, a beetle and some echinoderms (three ophiuroids, two sea stars, a holothurian and an echinoid). In addition, the same nutritive mode occurs or can be inferred during early development in poriferans, cnidarians, nematodes, molluscs, polychaetes, phylactolaemate bryozoans, and isopod and branchiopod Crustacea. While histotrophy dominates in terms of species numbers, the number of invertebrate phyla with this pattern (proven and inferred) is 15.

Placentotrophy also occurs or is inferred in 15 invertebrate phyla, including Porifera, Cnidaria, Platyhelminthes, Nemertea, Annelida, Mollusca, Arthropoda, Onychophora, Nematoda, Acoelomorpha, Gastrotricha, Rotifera, Kamptozoa, Cycliophora and Bryozoa. In most of these groups, there are only a few to several tens of placental species. Exceptions are Scorpionida (currently 1753 species), Bryozoa (∼844 matrotrophic species), gymnomeran Crustacea (all 37 species), and ∼5050 insects, including a beetle, all matrotrophic Dermaptera, Psocoptera, and Hemiptera (Tables 1 and 2).

In the ophiurids *Amphiura carchara* and *Amphipholis squamata* late‐stage embryos are positioned with their mouth and arms pressed against the wall of the bursa. Also, in the latter species the everted epithelium of the intestinal portion of the gut has elongated microvilli that are pressed against the bursal cuticle. If extraembryonic nourishment occurs in these instances, they both should be considered as special examples of placentation.

The distribution of matrotrophic modes within phyla is instructive. As with the distributional pattern of matrotrophic sites, some phyla show a very restricted range of nutritional modes whereas others employ different variants. The broader the range of incubation sites in a particular phylum, the more diverse are the nutritional modes employed. Most (16) of the phyla having matrotrophic species exhibit only one or, rarely, two modes of EEN. By contrast, several phyla exhibit all five (Chordata) or four (Platyhelminthes, Arthropoda, Echinodermata) known modes. There are no known examples of oophagy in Arthropoda or embryophagy in Platyhelminthes. Echinoderms show no proven examples of placentotrophy although the two ophiurid examples above may represent evidence for the opposite. Three nutritional modes are known in Mollusca and Nematoda. We are inclined to speculate that while undocumented, histotrophy is likely widely distributed in both phyla too, especially during early embryogenesis.

Similar to the change in the site of matrotrophic nutrition, a shift from one nutritive mode to another at different stages of embryonic development occurs in some groups (mentioned for vertebrates by Blackburn, 2014). The most common example is transition from histotrophy to histophagy (sometimes associated with oo‐ and embryophagy) after formation of the embryonic digestive system. It is known in isopod crustaceans and pseudoscorpions, flies of the genus *Miastor*, and is inferred for the holothurian *Synaptula hydriformis*. It probably also occurs in matrotrophic polychaetes, some nematodes, molluscs, and some other echinoderms. Matrotrophic modes also can intergrade: oophagy can grade into embryophagy, and histotrophy into placentotrophy. In the latter case, intergradation accompanies embryonic growth in the branchiopod crustaceans, onychophorans, and almost certainly in some bivalve molluscs and matrotrophic ascidians. In many matrotrophic bryozoans of class Gymnolaemata, which are mostly brooders, the growing embryo is initially resourced in the incubation chamber by histotrophy, being appressed to its wall for placentotrophy only in the final period of incubation when it occupies most of the brood cavity. In fact, two modes of EEN are present simultaneously during this period: while part of the embryo surface is in placental contact with a parent, another part can perform histotrophy (Moosbrugger *et al*., 2012). The same presumably occurs in phylactolaemate bryozoans: growth of the embryo during incubation in a brood sac is initially supplemented only by nutrient absorption (histotrophy), with the placental contact established later. Formation of this contact, however, presumably does not preclude histotrophy.

Examples that reverse this pattern evidently exist in katoikogenous scorpions: beginning with (inferred) placentation via a trophamnion, their EEN continues as histophagy. Placentation shifts to nourishment by the ‘queer feeding’ organ piercing the mesosoma in the apoikogenous scorpion *Lychas tricarinatus* (Mathew, 1962, p. 227; Farley, 2001). In the onychophoran *Peripatus acacioi*, nutrient provisioning begins with histotrophy, continues as placentation, shifts back to histotrophy, and then to histophagy. All the above variants (shifts in mode, intergradation and simultaneous occurrence) are also mentioned in discussions of vertebrate matrotrophy by Blackburn (2014).

### (5) Mechanisms of nutrient delivery and uptake

Matrotrophic modes are based on several physiological mechanisms providing nutrient delivery and uptake, and including secretion (apocrine and merocrine = exocytosis), active transport across membranes, diffusion, endocytosis and ingestion of parentally derived nutritive material. Endocytosis includes pino‐ and phagocytosis, and in different animal groups these two methods are involved in histotrophy and placentotrophy. For example, phagocytosis is performed by trophoblast cells in placental mammals (Wooding & Burton, 2008). Phagocytosis is known in some matrotrophic sponges, and theoretically might occur during development of the dwarf males and chordoid larva that grow at the expense of degenerating parental tissues in Cycliophora. Also, in the phylactolaemate bryozoan *Plumatella fungosa*, embryonic cells of the placental contact were described as ‘digesting’ the cells of the brood sac (Braem, 1897).

In an evolutionary context, the early heterochronous formation of functional mouthparts and a gut in developing embryos likely facilitated oophagy, embryophagy and histophagy (Blackburn, 2014). Histotrophy and placentotrophy can begin at much earlier developmental stages, even during cleavage, involving the same cytological mechanisms, ranging from diffusion to active transmembrane transport and endocytosis. This pattern is shown in the bryozoan *Bicellarialla ciliata* (Moosbrugger et al., 2012).

Nutrients are transported to the site of incubation from maternal tissues (for example, parenchyma, fat body) or a maternal gut. In the monogenean flatworms *Pseudodiplorchis americanus* and *Neodiplorchis scaphiopodis*, Cable & Tinsley (1991) described the close spatial arrangement of digestive and reproductive systems, suggesting a route for the continuous transfer of nutrients from parent to offspring. The intestine of these species comprises highly branched anastomosing caeca that interdigitate with loops of the uterus. Digestive epithelia lie in close proximity to the glycogen‐rich parenchyma surrounding the uterus. Similarly, Wilke (1954) ascribed to the gut an important role in the nourishment of the embryo of the gastrotrich *Urodasys viviparus*.

The wall of the incubation chamber often serves primarily for nutrient transfer, as Cable, Harris & Tinsley (1996) suggested based on the uterine ultrastructure of monogeneans. In the matrotrophic cockroach *Diploptera punctata*, it was shown that the uterus does not synthesize carbohydrates for developing embryos, merely transporting them from cells of the maternal abdominal integument (Youngsteadt *et al*., 2005). In the tapeworm *Oochoristica anolis*, parenchyma rich in glycogen and lipid droplets surrounds the syncytial uterine wall, which appears only to transport material for embryonic nutrition (Conn & Etges, 1984).

By contrast, synthesis and secretion of proteinaceous material by uterine cells has been recorded in the dermapteran insect *Arixenia esau* (Tworzydlo, Kisiel & Bilinski, 2013). Analogously, the ultrastructure of the epithelium‐derived embryophore in cheilostome bryozoans and of the cytoplasmic projections of the uterine wall in the cestode *Fimbriaria fasciolaris* indicate that these organs develop a powerful synthetic apparatus (Woollacott & Zimmer, 1975; Chomicz, 1996; Moosbrugger *et al*., 2012). In freshwater unionid and marine teredinid bivalves, the epithelial cells that nourish larvae contain large amounts of glycogen (Calloway, 1982; Schwartz & Dimock, 2001). Also, ‘voluminous mucous cells’/‘thick glandular cells’ develop during brooding in the epithelium of the interlamellar septa of the inner demibranchs or marsupial sacs in corbiculid and sphaerid Bivalvia, correspondingly (Gilmore, 1917; Okada, 1935; Morton, 1977; Byrne *et al*., 2000). Uterine cells produce a secretory product that is located apically in them as prominent bodies called ‘mucus buds’ in the onychophoran *Epiperipatus biolleyi* (Brockmann *et al*., 1999).

Transported and/or synthesized nutrients are released to the incubation cavity by exocytosis (most known instances) or apocrine secretion (ascidians, some nematodes). In this context, the potential role of diffusion should not be overlooked. In the dermapteran insect *Arixenia esau* uterine cells are equipped with long microvilli, the tips of which are covered with a ‘layer of electron dense fibro‐granular material’ during embryo incubation (Tworzydlo *et al*., 2013: p. 3). Extracellular matrix (also called ‘electron‐dense material’, ‘amorphous material/substance’, ‘flocculent material’, etc.) in the incubation cavity also has been recorded in several sponges (Vacelet, 1979; A.V. Ereskovsky, unpublished data), a turbellarian (Gremigni & Domenici, 1977), a monogenean (Cable & Tinsley, 1991), three cestodes (Conn & Etges, 1984; Chomicz, 1996; Korneva *et al*., 2014), two nematodes (Ellis *et al*., 1978), two cheilostome bryozoans (Woollacott & Zimmer, 1975; Moosbrugger *et al*., 2012), and an onychophoran (Campiglia & Walker, 1995).

The consumption of nutrients during histotrophy and placentotrophy takes place *via* diffusion, active transport across membranes or pinocytosis. The latter mechanism is well known in both chordates and invertebrates. Transmission electron microscopy (TEM) images of intrauterine embryonic nutrition in the turbellarian *Mesostoma ehrenbergii* (Domenici & Gremigni, 1977; Gremigni & Domenici, 1977) and the monogenean *Pseudodiplorchis americanus* (Cable & Tinsley, 1991) are strikingly similar to those in gymnolaemate bryozoans (Woollacott & Zimmer, 1975; Ostrovsky & Schwaha, 2011; Moosbrugger *et al*., 2012). In flatworms, both uterine and peripheral cells of the developing embryo produce cytoplasmic extensions/folds/microvilli toward the uterine lumen, in which amorphous material is accumulated. Similarly, a microvillous cell surface has been recorded in both the embryophore and embryo of bryozoans together with flocculent material in the incubation cavity. While Cable & Tinsley (1991, p. 264) stated, ‘whether the microvillous projections are involved in transport is unknown’ in a monogenean, it was proven experimentally that [radio‐labelled] nutrients cross the thin wall of the egg covering in *M. ehrenbergii* (Gremigni & Domenici, 1976, 1977). Endocytosis by embryo cells was also shown by TEM images in this species. Similar evidence confirms endocytosis in the bryozoan embryo and also in the embryophore, indicating removal of waste products (Moosbrugger *et al*., 2012).

In the onychophoran *Peripatus acacioi*, microvilli develop on both basal (ad‐uterine) and apical (ad‐embryo) surfaces of the syncytial placenta, indicating its probable active role in the uptake of nutrients from the maternal haemocoel to the fluid‐filled embryo sac. The surface of the young embryo and its stalk are covered by short microvilli with pinocytotic vesicles at their bases (Campiglia & Walker, 1995). Microvilli are also present on the embryo surface during the post‐placental period (Walker & Campiglia, 1988). Both the uterine wall and embryonic epithelial cells bear dense microvilli, presumably reflecting secretory and absorptive functions in another peripatid *Epiperipatus biolleyi* (Brockmann *et al*., 1999).

In a number of instances microvilli are developed either by the parent or by the offspring only. For example, in the unionid bivalves studied by Schwartz & Dimock (2001), the epithelium of the interlamellar septa, and the septa of secondary water tubes, was covered with numerous branched and unbranched microvilli, thus increasing the surface area delivering nutrients to the incubation cavity and the area of contact between larval and parental tissues. By contrast, the microvilli‐rich surface of velum of the veliger is characteristic of the bivalve *Corbicula australis* (Byrne *et al*., 2000).

Similarly, in the nematodes *Dipetalonema viteae, Dirofilaria immitis* and *Setaria cervi* it is only the uterine wall that develops an extensive system of microvilli (Ellis *et al*., 1978). In the ophiuran *Amphioplis japonicus* elongated microvilli were detected on the epithelium of the gut of near‐term juveniles. This part of the gut was slightly everted through the mouth and pressed against the bursal cuticle of the parent (Walker & Lesser, 1989).

It should be noted here that in viviparous and brooding matrotrophs, the cells that form the walls of incubation chambers have very different origins (cell sources), suggesting that their nutritive function was acquired convergently. Evolutionary convergence is also implied in cytological mechanisms for the delivery of nutrients to the incubation cavity as well nutrient uptake by the progeny.

In a number of cases, nutrient transfer occurs across a permeable barrier. In the turbellarian flatworm *M. ehrenbergii*, this barrier is the embryonic capsule; in the matrotrophic Neodermata and Acanthocephala it is the eggshell; in bugulid bryozoans it is the cuticle of the embryophore. In matrotrophic isopod crustaceans and the onychophoran *Peripatus acacioi*, nutrients cross the cuticle (underlain by microvilli) of the parental cotyledons and embryonic legs, correspondingly (Walker & Campiglia, 1988; Hoese & Janssen, 1989).

### (6) Structural complexity of invertebrate nutritional adaptations

Structurally, the simplest mode of provisioning is the use of unmodified parental and embryonic epithelia/tissues for nutrient transport. This occurs, for instance, when the embryonic epithelium takes up soluble nutrients *via* diffusion, or active transport across membranes or endocytosis, and no special temporary structures are formed *de novo* in either partner (parent and offspring). These physiological mechanisms represent EEN in cases of coelomic and pseudocoelous viviparity (polychaetes, digenean parthenits, acanthocephalans, a loriciferan, holothurians), embryonic incubation in parenchyma (in some rhabdocoel flatworms and during development of dwarf males and chordoid larvae in cycliophorans) and mesoglea (acroporid corals), or intracellular development in dicyemids and orthonectids (see online Appendix S1 for details and references).

By contrast, specialized temporary nutritive organs or tissues may develop *de novo* or form by modification of pre‐existing organs/tissues during incubation, and they can be acquired either by a parent, offspring, or both. Highly diffuse evolutionary trends or grades in matrotrophic complexity are suggested by comparative indications drawn across invertebrate taxa. They involve increasing structural complexity in morphologies, including (*i*) the formation of placental analogues – local zones of enhanced nutrient transport (whether simple contacts or specialized parental–embryonic cell complexes) from parental to embryonic cells directly or *via* a slit‐like space between them. Simple placental contacts comprise merely tight apposition of the parent's and progeny's tissues, often with no obvious morphological trace of specialization. Blackburn (1992), for example, discusses such contacts in squamate reptiles. In many cases, however, cells of the incubation chamber multiply, form syncytia, hypertrophy, develop synthetic apparatus, acquire microvilli, and so on. Such localized changes could also include the formation of various appendages that increase the total secretory surface and contact surface with the embryo (some Cestoda, Monogenea and Isopoda). Further indications of increasing complexity include (*ii*) compartmentalization of the common incubatory space into semi‐isolated chambers, inside which transport of parental nutrients may be more effective (some bivalve Mollusca, Cestoda, Monogenea, Onychophora, aphid insects, scorpions), and (*iii*) formation of specialized internal secretory (‘milk’) glands (some Diptera and all katoikogenous scorpions) (a shift in ovarian function to secrete nutritive fluid in Pseudoscorpionida should be also considered in this context).

Modified parental cells comprising the wall of the incubation chamber represent the simplest ‘exchange’ structure. In several calcareous sponges, early embryonic stages are accompanied by modification of the choanocytes of the closest choanocyte chamber, forming a ‘nutritive’ or ‘placental membrane’ (Gatenby & King, 1929; Duboscq & Tuzet, 1933; Gallissian, 1983) or ‘epilarval trophocyte epithelium’ (Lanna & Klautau, 2012). In other matrotrophic Porifera, the embryo is surrounded by a ‘follicle’ with a presumed nutritive function, in which cells can be either modified choanocytes, pinacocytes, or mesohylar (Ereskovsky, 2010). Likewise in cyclostome bryozoans, multiple cell layers of peritoneal origin (so‐called ‘secondary follicle’) wrap the early embryo. Later, multiplied cells of the transformed membranous sac (‘nutritive cylinder’) carry out nutrient transfer (Borg, 1926). Temporary hypertrophy of the cells of the brood‐chamber wall accompanies embryonic development in matrotrophic kamptozoans (Nielsen, 1990), gymnolaemate bryozoans (Woollacott & Zimmer, 1972, 1975; Moosbrugger *et al*., 2012; 2013a, 2013b), bivalves (Gilmore, 1917; Korniushin & Glaubrecht, 2003; Pettinelli & Bicchierai, 2009), synascidians (Mukai *et al*., 1997) and branchiopod crustaceans (Egloff, Fofonoff & Onbé, 1997). The epithelium of the uterine wall thickens and shows signs of active secretory activity in onychophorans (Anderson, Manton & Harding, 1972; Brockmann *et al*., 1999). Additionally, the second secretory layer develops over the vacuolated uterine epithelium in *Epiperipatus biolleyi*. Similarly, the integument of the skin‐brooding leech *Helobdella stagnalis* shows intense mitotic activity and differentiation of the secretory cells providing EEN (Cornec, 1978).

The uterine wall of some polystomatid monogeneans forms long cytoplasmic extensions that wrap early embryos and produce multilayered capsules through which nutrients are transported (Cable & Tinsley, 1991). Various cytoplasmic projections are formed by the uterine epithelium in matrotrophic cestodes. In some, these projections envelop early embryos (Conn & Etges, 1984; Chomicz, 1996), as in monogeneans, sometimes being closely connected with the highly folded outer embryonic envelope. Similarly, the marsupia of isopod crustaceans bear microvillar cotyledons that are absent in non‐matrotrophic species (Hoese & Janssen, 1989). Microvilli develop on the incubation chamber wall (uterine or of a brood chamber) in some matrotrophic flatworms (Domenici & Gremigni, 1977; Cable & Tinsley, 1991), nematodes (Ellis *et al*., 1978), bivalves (Schwartz & Dimock, 2001), bryozoans (Woollacott & Zimmer, 1975; Moosbrugger *et al*., 2012), an insect (Tworzydlo *et al*., 2013), and two onychophorans (Campiglia & Walker, 1995; Brockmann *et al*., 1999) (see also Section IV.5).

Close apposition of maternal and embryonic tissues invites use of the term ‘placenta’ or ‘placental analogue’. Simple apposition occurs in many of the instances noted above, but the most complex invertebrate placental analogues occur in Onychophora and Arthropoda. The placenta in the onychophoran *Peripatus acacioi* is a swollen syncytial area of uterine wall which increases in size during incubation (Walker & Campiglia, 1990; Campiglia & Walker, 1995). Once the embryo has become attached to the uterus *via* a stalk, cells of embryonic origin cover developing placental syncytium from the side of the incubation cavity and form an ‘embryo sac” (see also Section IV.5). So‐called ‘ring placentas’ of the thickened uterine wall encircling an incubation cavity and the embryo sac are formed in two other onychophoran species (Anderson *et al*., 1972). In apoikogenous scorpions, a ‘follicular placenta’, together with a ‘trophic lobe’, develops in the wall of the ovariuterine tube, finally abutting the embryo (Farley, 1996, 1998). Similar thickened parts of the follicle wall, or ‘maternal pseudoplacentae’ (Heymons, 1912; Hagan, 1951) are formed in the dermapteran insect *Hemimerus talpoides*. As in onychophorans, in arthropods, embryonic membranes are in intimate contact with the maternal part of the placenta and involved in transport of nutrients. Among invertebrate chordates, the most complex placenta develops in salps. It is formed from the thickened uterine wall and, when mature, comprises cortical and central syncytial layers that isolate the embryonic blood space from the central placental cavity, the walls of which are bathed by maternal blood (Bone, Pulsford & Amoroso, 1985).

Compartmentalization of the common incubatory space into isolated or semi‐isolated chambers, presumably enhancing the effectiveness of EEN, is known in both brooders and viviparous matrotrophs. Whereas the embryos of most matrotrophic bivalves are brooded inside the water tubes of the demibranchs, sphaeriids incubate their progeny inside brood sacs formed from the epithelium of the mollusc's gills (Gilmore, 1917; Groenewegen, 1926; Heard, 1965, 1977; Meier‐Brook, 1970; Pettinelli & Bicchierai, 2009). In fact, increase in offspring size occurs first in the marsupial sacs then extramarsupially between the gill filaments. Inasmuch as extramarsupial juveniles can theoretically begin normal feeding on suspended matter (Beekey, Karlson & Greenberg, 2000; Guralnik, 2004, see also Kraemer & Galloway, 1986, on corbiculid species), only the marsupial period of incubation is accompanied by EEN. We caution that many authors have not differentiated between these periods, measuring instead total larval increase (see Appendix S1).

In some matrotrophic Cestoda, the development of projections on the internal surface of the uterine wall results in subdivision of its cavity into numerous chambers containing one to several/many embryonated eggs (complex eggs containing embryos) (Conn, 1993; Chomicz, 1996; Korneva *et al*., 2014). In Onychophora, apoikogenous scorpions and aphidoidean insects, embryos respectively occupy discrete swollen parts of the uterus, ovarian tube or ovariole, being separated by much narrower ‘contracted’ zones (Manton, 1949; Mathew, 1960; Anderson *et al*., 1972; Francke, 1982; Miura *et al*., 2003). In katoikogenous scorpions, each embryo develops inside a separate diverticulum of the ovariuterine tubules (Mathew, 1968).

‘Milk glands’ of some arthropods are another structure whose secretions are swallowed by the developing offspring. These organs derive from follicle cells in katoikogenous scorpions (Mathew, 1968; Farley, 2011) and from spermathecae in at least four dipteran families (Meier, Kotrba & Ferrar, 1999), showing an interesting example of apparent change in the function of an organ in a reproductive system.

Certain major structural innovations enhance nutrient uptake in embryos. These include cytoplasmic appendages of embryonic envelopes that serve to enlarge the area of contact with the uterine wall (some matrotrophic Cestoda) (Chomicz, 1996; Korneva, 2005; Korneva, Kornienko & Guljaev, 2010; Korneva *et al*., 2014). The surface area for nutrient uptake is evidently enlarged in the greatly extended pleuropodia of the cockroach *Diploptera punctata* (Hagan, 1951) and by the barbed projections at the ends of the developing legs in the onychophoran *Peripatus acacioi* (Walker & Campiglia, 1988; Campiglia & Walker, 1995). Similar absorptive function is suggested for the ‘trophic vesicle’ known in some peripatopsid onychophorans with EEN, and described as a ‘swollen sac of dorsal extra‐embryonic ectoderm’ (Anderson, 1973, p. 4–16; see also Eriksson & Tait, 2012). In the gastropod *Fijidoma maculata*, the richly lobed larval velum presumably serves as a placental analogue (Hubendick, 1952). Embryos are surrounded by a stalked sac appressed to the thick glandular part of the uterine wall in the gastropod *Tekoulina pricei* (Solem, 1972), and massive calcium transfer from the uterine wall to embryos through the larval organ (‘placenta‐like podocyst’) occurs in *Stylodon studeriana* (Tompa, 1984).

A unique nutritional structure evolved in the apoikogenous scorpion *Lychas tricarinatus*; the dorsal side of its embryos possesses a hollow appendage with a terminal globular body through which nutrients are transported directly to the midgut (Mathew, 1960, 1962). The appendage derives from the extraembryonic cells and the nutrients are presumably transported by the globular body from the maternal haemocoel (while the embryo develops in the ovarian tube) (Farley, 2011). In pseudoscorpions, ovarian nutritive secretions are moved to the external brood chamber (Weygoldt, 1969).

Attachment structures (in some instances called ‘umbilical cords’ or ‘placental stalks’) are found in the embryos of some Nemertea, Echinodermata and Onychophora (Anderson *et al*., 1972; Oguro, Shosaku & Komatsu, 1982; Walker & Campiglia, 1988; Gibson, 1990; Campiglia & Walker, 1995). In phylactolaemate Bryozoa, the embryo is attached to the wall of the brood sac by the ‘spot‐’ or ‘ring‐like placenta’ (Braem, 1897, 1908). In nearly all cases, their precise role in nutrition is unclear. An exception is the onychophoran *Peripatus acacioi,* in which short microvilli on the stalk surface are evidence of absorption (Campiglia & Walker, 1995).

In scorpions and matrotrophic insects, embryonic membranes clearly participate in the transport of nutrients, representing the offspring's contribution to the placental analogues (Hagan, 1951; Polis & Sissom, 1990; Farley, 2011). A similar structure of embryonic origin (so‐called ‘embryo sac’) is formed in some peripatid onychophorans (Anderson *et al*., 1972; Anderson, 1973; Walker & Campiglia, 1988; Campiglia & Walker, 1995). Its cells are tightly appressed to the uterine wall including the placental region. Notably, the stalk and embryo sac are not found in the confamilial *Epiperipatus biolleyi*. In this species, the embryo is surrounded by a ‘non‐cellular coat’ of unknown nature with numerous transport vesicles, and the uterine wall shows pronounced secretory and transport activity (Brockmann *et al*., 1999). It should be added here that in the recent literature matrotrophic onychophorans are divided into placentotrophic (matrotrophic Peripatidae) and non‐placental (matrotrophic Peripatopsidae) (Manton, 1949; Anderson, 1973; Mayer *et al*., in press). Accepting Mossman's (1937) definition at least some peripatopsids possess placentotrophy because their embryos have large “trophic vesicle” that is appressed to the uterine wall (Anderson, 1973; Mayer, 2007; Mayer *et al*., in press), thus forming a simple placental contact. In the scyphozoan *Stygiomedusa gigantea*, the ‘cyst’ or ‘chorion’ surrounding the scyphistoma brooded in the stomach invagination might function in a similar way, although Russell & Rees (1960) suggested that the chorion draws nourishment directly from the stomach lumen of the parent medusa using its paired tube‐like projections.

Spot‐ and ring‐like placentas of phylactolaemate bryozoans present a rare case in which cells of the embryo are implanted in a brood‐sac wall. Moreover, Braem (1897) reported that embryonic cells of the placental interface in *Plumatella fungosa* can ‘digest’ the cells of the brood chamber, thus mirroring activities of the trophoblast in mammals. Similarly, the cytoplasmic outgrowths of the egg capsule are sometimes ‘inserted into the [uterine wall] as roots’ in some matrotrophic cestodes (Korneva, 2005, p. 555; Korneva *et al*., 2010, 2014).

These diverse structures are among the most striking examples of convergence. Achievement of close contact between parental and embryonic tissues is the consistent theme emerging from the entire range of structures, from simple apposition of the embryo to the wall of the incubation chamber to the invasion of embryonic envelopes or embryonic cells in the maternal wall (as occurs in some cestodes and phylactolaemate bryozoans, although the nutritive function of such placental analogues has not yet been demonstrated experimentally). In some arthropods and onychophorans nutritional organs are astonishingly similar to placentas in vertebrates, showing that convergent evolution acts at all levels – positional, structural and physiological. For instance, the nutritional complex comprising the embryo sac (of embryonic origin) and placenta (of maternal origin) in the peripatid onychophorans is analogous to the noninvasive epitheliochorial placenta of some mammals (Anderson *et al*., 1972; Anderson, 1973; Campiglia & Walker, 1995; see also Wooding & Burton, 2008). Analogous ‘two‐component’ placentas evolved in apoikogenic scorpions and the dermapteran insect *Hemimerus talpoides* (Hagan, 1951). As in vertebrates, EEN occurs in the sexual duct, involving a parent–offspring cell complex. Nutritive mechanisms also can be very similar: in the blue shark *Prionace glauca* epithelial cells of the yolk‐sac placenta are microvillous and the space between maternal and fetal epithelia is filled with electron‐dense material, suggesting exo‐ and endocytosis (Otake & Mizue, 1985). On the other hand, some nutritional adaptations are found only in invertebrates; examples include various nourishing appendages and internal ‘milk glands’ formed by the parent. In general, chordates have nutritive organs that are more complex, but similar organs are structurally and positionally more diverse across invertebrates.

### (7) Multiple independent origins of matrotrophy across Animalia

We consider the high diversity and scattered distribution of matrotrophy in phylogenetic trees to be evidence of its multiple independent evolution in Animalia. Our estimate of the number of separate origins of parental resourcing of offspring prior to their birth/release is based on the pattern of distribution of oviparity, non‐matrotrophic (lecithotrophic) incubation and matrotrophic incubation across phyla.

We support the generally accepted assumption that oviparity is an ancient reproductive mode from which embryonic incubation (both viviparity and brooding, matrotrophic and non‐matrotrophic) originated numerous times. On the other hand, some recent data show that viviparity and matrotrophy can be lost as well as gained (e.g. Riesgo *et al*., 2013, see also below). The liberation of ova to the external environment (oviparity or oviposition into a brood chamber) is present in most (29) animal phyla. Exceptions are Placozoa, Cycliophora, Orthonectida, Dicyemida and Acanthocephala, which are all viviparous, with brooding (during asexual reproduction) also in cycliophorans. Six phyla are exclusively oviparous (Sipuncula, Nematomorpha, Tardigrada, Gnathostomulida, Kinorhyncha, Xenoturbellida), and spawners comprise the vast majority of taxa in most of the remaining phyla. While hardly conclusive evidence, this dominance is at least consistent with the assumption that oviparity is a plesiomorphic condition.

We found no reports indicating matrotrophy for 5 of the 28 phyla in which embryonic incubation is known to occur. These phyla include species with broadcasting release and with embryonic incubation. Oviparity and lecithotrophic viviparity occur in Priapulida, and oviparity and lecithotrophic brooding are present in Chaetognatha, Phoronida, Brachiopoda and Hemichordata. Ctenophora is predominantly oviparous, but also includes two brooding and one viviparous species. If the latter is matrotrophic, then EEN has evolved independently in this phylum. Species in an additional phylum, Placozoa, gestate their embryos until the death of the parent, with further development taking place in the external medium.

In the remaining 21 phyla, matrotrophy is either established according to our criteria or strongly inferred from indirect evidence. Sixteen phyla include both oviparous and incubating (lecithotrophic and matrotrophic) species. Five other phyla are represented exclusively by incubating species. Kamptozoa are brooders, whereas Acanthocephala, Orthonectida and Dicyemida are viviparous. Both brooding and vivipary are present in Cycliophora.

Notwithstanding recent progress in molecular phylogenetics, the resolution of lower‐level taxonomic detail in phylogenies of animal phyla varies substantially with sampling distributions and densities, and with gene selection and analytical methods. Consequently, the position of many matrotrophic taxa is imprecise or in certain cases even unknown. We have attempted to cope with these varying levels of imprecision in our estimates of matrotrophic origins, especially with reference to reproductive character states and hypothesized phylogenetic relationships often well above the species level. Thus, our analysis should be considered as a first, preliminary estimate of the number of matrotrophic origins within phyla. We now briefly consider each phylum, providing examples from disparate data sources.

Blackburn (2014) estimated that substantial matrotrophy evolved at least 33 times among vertebrates (fishes, reptiles, amphibians and mammals) (see also Blackburn, 1992, 2005). Family Syngnathidae should be added to this list, as EEN occurs in a sealed brood pouch of the males in the ‘patrotrophic’ genera *Hippocampus* and *Syngnathus* (Carcupino *et al*., 2002). Among Tunicata, most colonial ascidians incubate their progeny, and some species are matrotrophic. Doliolids and pyrosomatids are viviparous–lecithotrophic, and salpids are all viviparous–matrotrophic. Based on a recent molecular phylogeny (Tsagkogeorga *et al*., 2009), it is likely that nutrient‐resourcing of embryos evolved independently in ascidians and salps, which have different nutritive structures. Moreover, it seems that EEN evolved twice in the phylogenetically distant families of brooding and viviparous ascidians (Stach & Turbeville, 2002; Tsagkogeorga *et al*., 2009). Hence there appear to have been at least 37 originations of matrotrophy among Chordata.

In **Porifera**, oviparity is known only in the Demospongiae, which also includes lecithotrophic and matrotrophic larviparous species, and species with direct development (Ereskovsky, 2010; Riesgo *et al*., 2013). Three other sponge classes—Hexactinellida, Calcarea and Homoscleromorpha—are all larviparous, and there are matrotrophic species in each. Demospongiae is often considered to be a sister group to Hexactinellida, and Calcarea is more closely related to Homoscleromorpha. The two latter classes are considered as a clade sharing a common ancestor with Demospongiae–Hexactinellida (Dohrmann *et al*., 2008; Philippe *et al*., 2009; Gazave *et al*., 2010; Wörheide *et al*., 2012). Riesgo *et al*. (2013) argues that viviparity is an ancestral character state in Porifera, with oviparity secondarily acquired in Demospongiae. Families with EEN are insufficiently sampled in the molecular analysis, and the number of independent origins of matrotrophy among Porifera might be better estimated at present by comparing the developmental origins of cells providing nourishment to embryos. We suggest at least four such independent origins—once in Calcarea (from choanocytes), once in Homoscleromorpha (from endopinacocytes) and twice in the clade Demospongiae–Hexactinellida (from pinacocytes and cells of the mesohyl). This estimate should be considered preliminary, since the formation of trophic structures (‘placental membrane’ and ‘nutritive capsule’) differs in different calcareous sponges.

The majority of **Cnidaria** spawn gametes directly to the aquatic environment. Embryo retention is known in some anthozoans, scyphozoans and hydrozoans. Whereas most corals are oviparous, embryonic incubation exists in both octo‐ and hexacorals (sometimes accompanied by feeding on particulate matter in the gastrocoel, reminiscent of juvenile nourishment in the mantle cavity in some bivalves, see Section IV.6), but matrotrophy is suggested only in two species of Acroporidae (Hexacorallia). Among Medusozoa that are predominantly oviparous, embryonic incubation is known in some Scyphozoa and Hydrozoa. Only two matrotrophic scyphozoan jellyfish species have been recorded in the order Semaeostomeae, in the phylogenetically distant families Pelagiidae and Ulmariidae (Dawson, 2004; Bayha *et al*., 2010). One of these species incubates larvae in the ovary; the other broods them in specialized brood chambers—protrusions of the stomach. Among the mostly oviparous hydrozoans, matrotrophic incubation that begins in the ovary and continues as brooding is known only in one species of Trachymedusae. Thus, the phylogenetic distribution (Collins *et al*., 2006) of matrotrophy and differences in the sites and modes of incubation strongly point to independent matrotrophic origins in three cnidarian classes, and twice in Scyphozoa (four in total).

In **Platyhelminthes**, matrotrophy exists in several species of free‐living rhabdocoel flatworms (turbellarians of the families Typhloplanidae and Graffillidae) and in several groups of parasitic Neodermata. Since the overhelming majority of turbellarians are oviparous, including in these two phylogenetically distant families (Willems *et al*., 2006), two independent origins of matrotrophy are indicated in this clade. Current molecular phylogenies of Neodermata consider classes Trematoda (Digenea plus Aspidogastrea) and Cestoda (Gyrocotylidea, Amphilinidea and Eucestoda) as sister groups, and class Monogenea as a sister group to this clade (Lockyer, Olson & Littlewood, 2003; Baguñà & Riutort, 2004; Willems *et al*., 2006; Caira & Littlewood, 2013). Oviparity (production of non‐embryonated eggs) is known in all three major neoderm clades. Intrauterine development of embryonated eggs also occurs here, evincing a link with intrauterine matrotrophy in some families. The phylogenetic distribution of these different reproductive patterns points to at least three independent acquisitions of matrotrophy, but many more appear likely. In Monogenea, matrotrophic viviparity is known in Gyrodactylidae and Polystomatidae, which belong to two major monogenean clades (Monopisthocotylea and Polyopisthocotylea, respectively). Both families include species producing either non‐embryonated or embryonated eggs. In Eucestoda, matrotrophy has been recorded in eight families (Nippotaeniidae, Tetrabothriidae, Nematotaeniidae, Proteocephalidae, Bothriocephalidae, Hymenolepididae, Dilepididae, Linstowiidae) belonging to five orders (Waeschenbach *et al*., 2007; Waeschenbach, Webster & Littlewood, 2012*a*). Bothriocephalidae (order Bothriocephalidea) includes species with embryonated (including one matrotrophic) and non‐embryonated eggs, whereas the four orders including the remaining seven families with matrotrophic species all appear to have embryonated eggs. These are all ‘oligolecithal’ (having few vitelline cells in the complex egg) and possess thin, non‐sclerotised embryonic envelopes (Swiderski & Xylander, 2000; Conn & Swiderski, 2008), suggesting EEN. We conservatively reason that matrotrophy may have evolved at least twice in this class. In the hermaphroditic generation of digeneans, matrotrophy is known in four phylogenetically distant families (Hemiuridae, Plagiorchiidae, Philophthalmidae, Heronimidae) (Olson *et al*., 2003), interspersed between clades with non‐embryonated eggs in the phylogenetic tree (Galaktionov & Dobrovolskij, 2003). While EEN may exist in some other families that produce eggs with fully formed miracidia, there are currently no supporting data. Thus, while secondary losses of EEN are theoretically possible, the mosaic distribution of matrotrophy in Neodermata suggests two originations in Monogenea, two in Cestoda and at least four in Digenea. In addition, based on the nutrient provisioning of embryos in the parental body cavity, matrotrophy is characteristic of all digenean parthenitae, suggesting a single origin for this additional kind of matrotrophic nutrition in Digenea. We suggest that maternal provisioning evolved at least 11 times in Platyhelminthes. Given the huge number of unstudied platyhelminth species, we anticipate discovery of more instances of independent origination of matrotrophy.

**Orthonectida** are all viviparous matrotrophs, incubating their sexual generation within a plasmodial stage. Similarly, nematogen and infusoriform larvae grow inside parental stages in all **Dicyemida**. The phylogenetic position of these two lophotrochozoan phyla is poorly resolved (Petrov *et al*., 2010; Suzuki *et al*., 2010; Dunn *et al*., 2014), but their bizarre incubational modes relative to other major taxa strongly indicate independent origins of matrotrophy.

The prevalence of non‐matrotrophic species among **Kamptozoa** suggests that EEN evolved secondarily in this phylum. Matrotrophy has been recorded in three species of Loxosomatidae and two species of Pedicellinidae in the two orders. Although all species nourishing their offspring form a placental analogue from the wall of the brood pouch, phylogenetic relationships within the phylum (Fuchs *et al*., 2010) strongly suggest at least one independent origin of EEN in each order, since all other species are non‐matrotrophic brooders. The alternative is numerous losses of parental provisioning in the phylum. In **Cycliophora**, united with Kamptozoa as sister groups in recent molecular studies (Hejnol *et al*., 2009), matrotrophic viviparity and matrotrophic brooding are exhibited by different generations (sexual and asexual, respectively) of the complex life cycle. Both incubational variants employ different modes of embryonic nutrition (placentotrophy and inferred histotrophy). Since all kamptozoans are brooders and cycliophoran females are viviparous, it is highly likely that EEN evolved independently in these phyla. Moreover, it has seemingly evolved twice in the same life cycle in cycliophorans, as also in digeneans.

Five species from the three classes of **Acanthocephala** are matrotrophic. Their acanthor larvae are incubated in the pseudocoel, presumably nourished by histotrophy. Inasmuch as the related **Rotifera** (Near, 2002; García‐Varela & Nadler, 2006) are almost entirely oviparous, it is reasonable to suggest that the viviparous Acanthocephala acquired matrotrophy independently. Moreover, if most acanthocephalans are lecithotrophic, then the distant phylogenetic positions of those families including matrotrophic species (Verweyen, Klimpel & Palm, 2011) could point to three independent origins of matrotrophy in this group (see also García‐Varela *et al*., 2000, 2002 for phylogenies). Only a few Rotifera are viviparous–lecithotrophic and one species is viviparous–matrotrophic. **Gastrotricha** are similarly all oviparous except for one viviparous matrotrophic species that incubates a single embryo in the uterus.

Compared with all aquatic invertebrates, **Bryozoa** have the widest within‐phylum taxonomic distribution of placental analogues, unusually diverse incubational structures, and numerous instances of incipient matrotrophy (Ostrovsky *et al*., 2009; 2013a, 2013b, see also Ostrovsky & Schäfer, 2003; Ostrovskii, 2004; Ostrovsky, 2008; Ostrovsky, Vávra & Porter, 2008). As a reproductive strategy, yolky eggs combined with low levels of parental provisioning is well known among vertebrates (Blackburn, 2005; Pollux *et al*., 2009, see also Section III), and considered by some workers as an initial step in the evolution of advanced substantial matrotrophy (Stewart & Thompson, 2003, 2009). Incipient matrotrophy almost certainly occurs in several invertebrate groups, for example, in scorpions (Farley, 2001). Yet until recently it was recorded (although not realized as such) only in a few insects (Bontems, 1984; see also discussion in Hagan, 1951). Our investigation showed that both non‐matrotrophic and matrotrophic brooders (with incipient and substantial placentotrophy) co‐occur in some bryozoan families and even genera, suggesting numerous independent evolutionary transitions from lecithotrophy to EEN (Ostrovsky *et al*., 2009; 2013a, 2013b). Possible reversions are presently unknown.

Apart from the simultaneous presence of different reproductive patterns within the same lower‐level taxa, additional evidence of multiple origins of extraembryonic nutrition in bryozoans is provided by the independent evolution of brood structures, different sequences of events during EEN (histotrophy *versus* placentotrophy), differences in the origin (cell source), position, anatomy and ultrastructure of placental analogues in different groups, and the current level of molecular‐phylogenetic resolution (Reed, 1991; Ostrovsky & Taylor, 2004, 2005; Fuchs, Obst & Sundberg, 2009; Ostrovsky *et al*., 2009; Knight, Gordon & Lavery, 2011; Waeschenbach, Taylor & Littlewood, 2012*b*; discussed in Lidgard *et al*., 2012; 2013a, 2013b). Matrotrophy evolved at least five times in the order Ctenostomata (class Gymnolaemata), as inferred from the distribution of reproductive patterns (Todd, 2000; 2013a, 2013b). It may have evolved more than 15 times in the gymnolaemate order Cheilostomata, based on the co‐occurrence of non‐matrotrophic and matrotrophic brooders in some families and even genera. Bryozoans of the order Cyclostomata (class Stenolaemata) and the cheilostome family Epistomiidae employ intracoelomic incubation that almost certainly originated independently. The same can be said for the class Phylactolaemata, which according to recent molecular analyses is the sister group of all other bryozoans. Thus, matrotrophy apparently evolved at least 23 times in Bryozoa.

Most **Nemertea** are egg‐laying except for a few viviparous species that either incubate their progeny in the ovary or in a female gonoduct with or without parental nourishment. Matrotrophic species belong to five phylogenetically distant families (Prosorhochmidae, Sacconemertidae, Tetrastemmatidae, Zygonemertidae, Emplectonematidae) within class Enopla. Each family also includes oviparous species (Stricker *et al*., 2001; Sundberg, Turbeville & Lindh, 2001; Thollesson & Norenburg, 2003; Chernyshev, 2005; Maslakova & Norenburg, 2008a). Thus, EEN appears to have evolved at least five times in Nemertea.

Spawning or egg laying are the dominant or exclusive modes of reproduction in all classes of **Mollusca**, and oviparity is widely regarded as plesiomorphic for this phylum. Aplacophora are all oviparous except for some Neomeniomorpha that brood their eggs in the mantle. Aplacophora may be a sister group of Polyplacophora, whose species are mostly oviparous, excluding about 30 brooders. Indicative viviparous species (Plate, 1899) have not been confirmed (B. I. Sirenko, unpublished data). A clade uniting Polyplacophora and Aplacophora is considered to be a sister group to Conchifera, which includes the wholly oviparous Cephalopoda and Scaphopoda (both having basal positions in this clade) (Kocot *et al*., 2011). Sister classes Bivalvia and Gastropoda each have oviparity (dominant) and embryonic incubation and both include matrotrophic species. In bivalves, matrotrophy is associated with brooding, whereas gastropods exhibit both brooding and viviparity, pointing to independent origins of EEN in these classes. Among gastropods, egg laying and lecithotrophic and matrotrophic incubation exist in Caenogastropoda (matrotrophy recorded/inferred in Thiaridae, Paludomidae, Planaxidae and Janthinidae), Patellogastropoda (Acmaeidae) and Heterobranchia (Achatinellidae, Acavidae and Veronicellidae), also suggesting independent matrotrophic origins in these three subclasses. Based on the distant phylogenetic position of the above‐mentioned families in these clades (Wade, Mordan & Clarke, 2001; Colgan *et al*., 2007; Dayrat *et al*., 2011), matrotrophy may have originated at least eight times in Gastropoda. Among Bivalvia, most of which are broadcast spawners, matrotrophic brooding is known in five families. Two (Unionidae and Hyriidae) are in the Palaeoheterodonta and three others (Corbiculidae, Sphaeriidae, Teredinidae) in three different clades of Neoheterodontei (Bieler *et al*., 2014; see also Graf, 2000; Giribet & Wheeler, 2002; Taylor *et al*., 2007). The distant position of these families in the bivalve phylogenetic tree suggests that matrotrophic nutrition evolved 5 times in this class and 13 times in Mollusca.

The vast majority of **Annelida** are oviparous and some are viviparous and brooders. Matrotrophy is known only among polychaetes and three species of leeches. Matrotrophic polychaetes are all viviparous, belonging to seven families (Ctenodrillidae, Cirratulidae, Nereididae, Spionidae, Syllidae, Ampharetidae, Geobangiidae) with distant positions in phylogenetic trees (Struck *et al*., 2007; Zrzavý *et al*., 2009), suggesting six independent origins of matrotrophy. The three known matrotrophic leeches (genera *Glossiphonia*, *Helobdella* and *Marsupiobdella*) are members of the obligate brooding family Glossiphoniidae. However, their phylogenetic position within the family (Siddall, Budinoff & Borda, 2005) suggests three independent origins of parental provisioning. Thus, the pattern of distribution of matrotrophy in the two annelid classes suggests that it evolved at least nine times in this phylum.

Matrotrophic viviparity occurs in both living families of **Onychophora**, but placentotrophy is structurally different in Peripatidae and Peripatopsidae (see Section IV.2). Both these families also include lecithotrophic viviparous species, and there are oviparous forms among peripatopsids. Mayer *et al*. (in press) mapped reproductive characters onto a recently published phylogeny (Murienne *et al*., 2014, see also Gleeson *et al*., 1998; Monge‐Najera, 1995), and suggested that the last common ancestor of Onychophora was either lecithotrophic–viviparous or that it combined lecithotrophy with matrotrophy. Depending on what was the case, EEN has evolved twice or once in this phylum, respectively.

Oviparity is the dominant mode of reproduction in **Arthropoda** although brooding and viviparity are common or even obligatory for subgroups at some intermediate taxonomic ranks. Most Chelicerata, considered to be the basalmost arthropod clade (Regier *et al*., 2010), are oviparous, strongly suggesting that oviparity is plesiomorphic for the phylum. By contrast, Scorpionida and Pseudoscorpionida are entirely matrotrophic; EEN appears to have evolved independently in these orders since species of the former exhibit viviparity whereas those of the latter have brooding. Two matrotrophic viviparous mites are in families (Spinturnicidae, Epidermoptidae) belonging to different superorders (Krantz & Walter, 2009), which together with the dominance of oviparity, suggest that Acari acquired matrotrophy at least twice.

Myriapoda, sister to Pancrustacea, are mostly oviparous, although some brooders are known. Matrotrophy is undocumented here, so oviparity is inferred to be ancestral in both Crustacea and Hexapoda. Among Crustacea, EEN occurs in the Branchiopoda and Malacostraca. Among branchiopods, matrotrophy is known in five families belonging to three cladoceran clades – Anomopoda (Moinidae), Ctenopoda (Sididae) and in all Gymnomera (Polyphemidae, Cercopagidae, Podonidae). The position of these clades with respect to each other, as well as the position of families within clades (Stenderup, Olesen & Glenner, 2006) points to three independent origins of matrotrophy in Cladocera. Among malacostracans, EEN is known in Decapoda (Hippolytidae) and Isopoda (Gnathiidae, Cirolanidae, Armadillidiidae, Armadilliidae, Porcellionidae, Ligiidae, Oniscidae, Hemioniscidae, Chaetiliidae). The phylogenetic position of those isopod families with matrotrophic species (Wilson, 1996; Mattern & Schlegel, 2001; Schmidt, 2008) suggests 7–8 independent origins in this group. Among hexapods, matrotrophy has been recorded in seven insect orders – Dermaptera, Diptera, Hemiptera, Coleoptera, Psocoptera, Blattoidea and Strepsiptera – the last being entirely matrotrophic. Since the other six orders are dominated by oviparous species, matrotrophy may have evolved independently in each. Support for this view is provided by the relative position of these orders in the hexapod phylogenetic tree (Kjer *et al*., 2006). In Diptera, the phylogenetic position of the wholly or partly matrotrophic families (Nirmala, Hypša & Žurovec, 2001) suggests 5–6 independent originations of EEN. A similar analysis suggests two originations in Hemiptera (Schuh, Weirauch & Wheeler, 2009; Ortiz‐Rivas & Martínez‐Torres, 2010), and three in Coleoptera (Hunt *et al*., 2007; Beutel, Ge & Hörnschemeyer, 2008). Thus EEN probably originated at least 14–15 times in Insecta (see also Hagan, 1951; Meier *et al*., 1999), and 29–31 times in Arthropoda.

Most **Nematoda** are oviparous, but a substantial number of species incubate their progeny *in utero*, and are either lecithotrophic or matrotrophic. It is likely that EEN evolved independently in both classes (Enoplea and Chromadorea) (for recent phylogenies see De Ley, 2006; Smythe, Sanderson & Nadler, 2006). In Enoplea, matrotrophy is known in three families of two orders, whereas among Chromadorea all known examples of matrotrophy are confined to order Rhabditida (18 families). Seven families of Rhabditida include both oviparous and matrotrophic species, the latter having a highly mosaic distribution in gene trees (Holterman *et al*., 2006; Meldal *et al*., 2007), indicating independent origins of EEN. Available data are not sufficient to draw firm conclusions; we conservatively estimate at least 10 independent transitions to matrotrophy among Nematoda.

Whereas most **Loricifera** are oviparous, both oviparity and viviparity (lecithotrophic and matrotrophic) occur in Pliciloricidae (Heiner & Kristensen, 2009). Both oviparous and viviparous species are known among **Acoelomorpha** (for phylogeny see Jondelius *et al*., 2011), and matrotrophy is inferred in two distant families (Diopisthoporidae and Childiidae). One and two originations of EEN are thus suggested for these phyla correspondingly.

Oviparity (broadcasting and egg laying) is predominant in all five extant classes of **Echinodermata** (for phylogeny see Janies, Voight & Daly, 2011), and is widely regarded as the ancestral character state. Matrotrophic brooding also exists in all classes. The structure and position of brood pouches as well as different mechanisms of nutrient uptake by embryos point to EEN evolving independently in all classes. The same can be claimed for matrotrophic viviparity, which occurs either in a coelomic cavity (Asteroidea, Holothuroidea) or is intra‐ovarian (Asteroidea, Ophiuroidea, Holothuroidea, Crinoidea). Among Ophiuroidea, three families with parental provisioning (Ophiuridae, Ophiacanthidae, Amphiuridae) are phylogenetically distant (Smith, Paterson & Lafay, 1995), and one brooding and one viviparous matrotrophic species co‐exist in the Ophiuridae. We infer four independent origins of matrotrophy in this class. There are five families with EEN among Asteroidea (Asterinidae, Asteriidae, Stichasteridae, Pterasteridae, Xyloplacidae), all phylogenetically distant (Janies *et al*., 2011; Mah & Foltz, 2011; Mah & Blake, 2012). The first four families include matrotrophic brooders, and Asterinidae also includes a viviparous matrotrophic species. By contrast, Xyloplacidae comprises only viviparous matrotrophs. Six originations of matrotrophic nutrition are suggested in this class. Among Holothuroidea, seven families (Cucumariidae, Phyllophoridae, Psolidae, Deimaidae, Synaptidae, Chiridotidae, Sclerodactylidae) include viviparous matrotrophs. Cucumariidae and Psolidae also include matrotrophic brooders. Only Synaptidae and Chiridotidae form a clade in both morphological (Kerr & Kim, 2001) and molecular (Lacey *et al*., 2005) analyses. Accordingly, we infer at least five independent origins of matrotrophy in Holothuroidea. More are likely, however, pending further research on holothurian reproduction. Among Echinoidea, matrotrophic brooding is known in the Urechinidae and Schizasteridae and the genus *Amphipneustes* (Paleopneustina). Urechinids are very distantly related to paleopneustines and schizasterids, which are close in gene trees (Kroh & Smith, 2010). We infer two independent originations of EEN in Echinoidea. Among Crinoidea, a viviparous matrotrophic species is known in the Comasteridae and a matrotrophic brooding species in the Notocrinidae, suggesting two originations. Therefore, we conservatively estimate at least 19 independent originations of matrotrophy in Echinodermata.

In summary, with respect to the incidence of independent EEN originations across the animal phyla, Chordata, Arthropoda, Bryozoa and Echinodermata are highest with 37, 29–31, 23, and 19, respectively. Matrotrophy apparently originated 13, 11, 10 and 9 times among Mollusca, Platyhelminthes, Nematoda and Annelida, respectively. In the majority of phyla with parental provisioning it evolved once.

We caution that both new or alternative phylogenetic hypotheses and new discoveries of EEN will affect our preliminary estimates of independent origins. The primary source of caution is that the distribution of matrotrophy in some phyla (e.g. Porifera, Nematoda, Mollusca, Arthropoda, Acanthocephala) is clearly understudied and thus likely to be underestimated. Second, while most authors tend to consider the acquisition of viviparity (and thus matrotrophy) from oviparous ancestors as predominant over loss (Wourms & Lombardi, 1992; Lee & Shine, 1998; Blackburn, 1999*a*; Shine & Lee, 1999; Pollux *et al*., 2009; Ereskovsky, 2010 and references therein), maternal input can be highly varied. For instance, the phylogenetic analysis of Dulvy & Reynolds (1997) showed 6–8 reversals from matrotrophic to lecithotrophic viviparity in elasmobranchs. A recent large‐scale phylogenetic analysis of squamate reptiles (Pyron & Burbrink, 2014) argues that ∼115 putative origins of viviparity in this group (of a claimed 150+ in vertebrates; Blackburn, 2014) should be reinterpreted based on evidence for an early origin of viviparity at the base of this clade. Their results suggest a complex pattern of multiple reversions to oviparity, a much‐reduced number of originations of viviparity, and consequent uncertainty about the origins of matrotrophy. Discussing this issue in respect to elasmobranchs, Blackburn (2014) stated that this problem will not be solved without a consensus on their phylogeny.

Among invertebrates, oviparity is thought to have arisen twice from viviparity in Onychophora (Reid, 1996). Also, Riesgo *et al*. (2013) suggested that viviparity (and, thus, matrotrophy) may have been be lost in some sponges. At finer scales, the evolution of varying degrees of matrotrophy and placentation can be very labile and strongly correlated with other life‐history characteristics and environmental factors (Chen & Caswell‐Chen, 2004; Lewitus & Soligo, 2011; Pires *et al*., 2011). Thus, estimates of the number and distribution of independent origins of matrotrophy should be combined with phylogenetic character mapping at several scales. Across Animalia as a whole, these tasks have only just begun.

### (8) Implications for evolution and ecology

Matrotrophic patterns encompass numerous structural and physiological variants. These variants reflect stages (often transitional) in the evolution of parental care and apparent trends in the transformation of parent–offspring cell–tissue relationships, studied most thoroughly in vertebrates (reviewed in Blackburn *et al*., 1985; 1992, 1999b, 2014; Wake, 1992; Wourms & Lombardi, 1992; Wooding & Burton, 2008; Hemberger, 2013). Recently Blackburn (2014, p. 20) highlighted the morphological and evolutionary principles that, together with the selective background, shape the evolutionary trajectories of matrotrophy in vertebrates. These are ‘constraints … due to phylogenetic inertia coupled with design limitations, … exaptation/preadaptation, and heterochrony.’ Examples include embryonic membranes that, on the one hand, prevent embryos from ingesting surrounding fluid/tissue/eggs/siblings, thus acting as a constraint in the evolution of certain matrotrophic modes, and, on the other hand, readily establish placental contact (preadaptation). Early development of the functional feeding apparatus and digestive tract in embryos, allowing them to ingest nutrients, can be interpreted as an example of heterochrony. Our results present evidence for similar trends and involvement of the same principles in invertebrates, promising a comparative context broader than vertebrates alone. The scope of different developmental, physiological, morphological, and life‐history patterns presents additional questions and challenges for a general understanding of the evolution and adaptive significance of matrotrophy.

One component in the evolution of matrotrophy and placentation is maternal–fetal conflict (Crespi & Semeniuk, 2004), important parts of which are cell–cell communication in regulation of embryonic development and nutrient provisioning, and mediation of deleterious immune responses. In contrast to the situation in vertebrates, where genomic conflicts between embryos and mothers are implicated as a potent evolutionary force in the evolution of life histories, reproductive structures, immune tolerance and maternal investment (Schrader & Travis, 2009; Banet, Au & Reznick, 2010; Wildman, 2011; Pollux *et al*., 2014), studies on invertebrates – including but not focusing on matrotrophy – are far fewer (e.g. Kamel, Grosberg & Marshall, 2010; Trumbo, 2012; Wong, Meunier & Kölliker, 2013), entreating biologists to broaden comparative studies. For instance, analysis of the members of the conserved molecular signalling pathways that act in concert during mammalian placenta formation (Sonderegger, Pollheimer & Knofler, 2010; Park & Dufort, 2011; Singh, Chaudhry & Asselin, 2011) could be a starting point for comparison of incipient and fully developed placental analogues in Bryozoa, representing an entirely separate model of placental evolution. Here, too, conflicts and maternal provisioning are already being explored (Marshall, 2008; Marshall & Keough, 2008).

Evolutionary changes in reproductive modes, placental nutrition, and matrotrophy in general are also considered largely adaptive, but this inference cannot be considered in isolation from entire life cycle and ecological contexts (Blackburn, 1992; Lombardi, 1996; Trexler & DeAngelis, 2003). Two different life‐cycle patterns are vividly apparent among many matrotrophic invertebrates, but not among vertebrates. First, many colonial matrotrophic invertebrates, including virtually all bryozoans, remain sessile once larval dispersal occurs. Their distribution is concentrated in patches in space and time, exposed to whatever water‐borne nutritional resources are available, and to predators from which they cannot flee. Mating is by ‘spermcasting’ (Bishop & Pemberton, 2006) and multiple inseminations by different genotypes may be common. This mode of life, with analogies among land plants (Graham & Wilcox, 2000), may have influenced the repeated acquisition and diversification of matrotrophy in bryozoans and the plasticity of maternal investment (Marshall & Uller, 2007; Marshall *et al*., 2008; Ostrovsky *et al*., 2009). Second, parasite life cycles in many invertebrate clades may be even more concentrated in both time and space to exploit host vulnerability and immediately infective transmission (Viney & Cable, 2011). Repeated evolution of matrotrophy among invertebrate parasites is tangled with a welter of adaptive factors and trade‐offs: size and age at infectivity, growth rate and resource intake, the timing of transmission before host death, and more (Tinsley, 2004, 2005). For example, in parasitic nematodes matrotrophy (and viviparity) may increase the rapidity of maturation, outpacing the threatening immune response of the final host, but may also act to ‘seclude’ the organism from the hazards of larval exposure in the external environment by reducing the transmission interval (Hugot & Quentin, 2000). Among other factors, matrotrophy (and viviparity) enable parasitic insects to reduce the vulnerability of early developmental stages, but this reproductive pattern has also been found in association with non‐parasitic lifestyles (Meier *et al*., 1999). In gyrodactylid monogenean flatworms, nearly all of which are matrotrophic parasites, acceleration of the life cycle and juvenile or larval reproduction (progenesis) undoubtedly contributed to the enormous diversity of this group (Boeger, Kritsky & Pie, 2003).

In summary, adaptive hypotheses explaining why matrotrophy and placentation evolved repeatedly in some animal groups but not others are numerous and sometimes conflicting (Houston *et al*., 2007; Marshall *et al*., 2008; Blackburn, 2014): maximizing fecundity when resources are abundant, allowing flexible energetic allocation in changing or unpredictable environments, lowering gestation times to permit additional broods, lowering risk to vulnerable early life stages by retaining offspring longer or accelerating their maturation, enhancing survivorship by producing fewer but larger and fitter offspring (including transgenerational effects), being impeded by immunological interactions or driven by parent–offspring conflicts, facilitating inoculation of offspring with beneficial symbionts, weakening precopulatory mate choice and more (Wourms & Lombardi, 1992; Crespi & Semeniuk, 2004; Marshall & Uller, 2007; Haine, 2008; Marshall *et al*., 2008; Ostrovsky *et al*., 2009; Pollux *et al*., 2009, 2014; Capellini, Venditti & Barton, 2011). Reasons for evolving matrotrophy across Animalia may turn out to differ among animal groups at different taxonomic scales, may be variable or facultative in some groups but not others, or may best be thought of as a continuum of reproductive and developmental processes. We cannot say at this point. But seeking a better understanding of the empirical distribution of matrotrophy is one step in that direction, encouraging a broader look beyond the first question – how and when did mom feed the embryo?

## V. CONCLUSIONS

(1) Our analysis shows that matrotrophy is demonstrated or inferred in two‐thirds of all animal phyla, i.e. 21 out of 34, changing the paradigm that it is comparatively rare in invertebrates. In fact, matrotrophy has an almost pan‐metazoan distribution. In some phyla EEN is represented by only one or few matrotrophic species; in others it is an obligate expression of parental care. Platyhelminthes, Arthropoda and Bryozoa dominate, with 162, 83 and 53 families, respectively, that are wholly or partly matrotrophic.

(2) The distribution of matrotrophy among and within phyla has led us to estimate from 140 to 145 independent originations in all superclades—among Parazoa and Eumetazoa, Radiata (Diploblastica) and Bilateria (Triploblastica), Protostomia and Deuterostomia, Lophotrochozoa and Ecdysozoa. Non‐matrotrophic phyla are also represented in all the above superclades. Matrotrophy evolved at least 29–31 times among Arthropoda alone, comparable with Chordata (37 times). In Cycliophora and some Digenea it evolved twice in the same life cycle. By contrast, matrotrophy has not evolved in phyla that either lack incubation of the embryo or which brood externally, thereby precluding nutritional exchange.

(3) Matrotrophy is associated with all known types of incubation chambers (external brood sacs excepted). Matrotrophic viviparity is more widespread, found in 20 phyla (excluding Kamptozoa), whereas matrotrophic brooding is known in 10 phyla. Nine phyla possess both types of incubation. As to the variety of matrotrophic incubation sites, arthropods and bryozoans exhibit the most diverse range. The most exotic variant is intracellular and intraplasmodial matrotrophy (in Dicyemida and Orthonectida).

(4) EEN in invertebrates is expressed by the five matrotrophic modes, of which histotrophy and placentotrophy (similar to chordates) are prevalent. Most phyla have a very restricted range of nutritional modes; a few have several. Platyhelminthes, Arthropoda and Echinodermata have as many as four, exceeded only by Chordata. As in vertebrates, matrotrophic modes can shift, intergrade and be performed simultaneously during gestation in invertebrates.

(5) Evolution of the nutritional organs that are formed either by a parent or offspring, or both, includes formation of (*i*) local zones of enhanced nutritional transport (placental analogues), including specialized parent–offspring cell complexes and various appendages increasing the entire secretory and absorbing surface and/or the contact surface between embryo and parent, (*ii*) compartmentalization of the common incubatory space into compact chambers with more effective nutritional relationships, and (*iii*) internal secretion (‘milk’) glands. Heterochronic (early) formation of mouthparts and digestive system in embryos appears to be an important innovation. Some placental analogues in Onychophora and Arthropoda mimic the simplest vertebrate placentae, constituting striking examples of convergent evolution acting at all levels—positional, structural and physiological. Generally speaking, invertebrate matrotrophic adaptations are structurally less complex than in chordates but are more diverse. Some nutritional organs, like internal ‘milk glands’ are known only in invertebrates.

(6) The broad distribution and high diversity of nutritional modes, structures, sites and mechanisms in invertebrates suggest high adaptive potential. However, matrotrophic and non‐matrotrophic species often coexist within the same clades, and the mosaic distribution of EEN is difficult to explain. Many more important questions remain unanswered. Our analysis is the first attempt to encompass the full range of invertebrate matrotrophy in one integrated picture, and we hope that it will promote more research in this field.

## Supporting information


**Appendix S1.** Distribution of matrotrophy among invertebrates and invertebrate chordates (proved, stated or inferred based on indirect evidence).
