# Supplementary material for: Matrotrophy and placentation in invertebrates: a new paradigm
Source: Biol Rev Camb Philos Soc. 2015 Apr 29;91(3):673–711. doi: 10.1111/brv.12189 (PMC5098176; doi:10.1111/brv.12189)
Supplement: Supplementary file 1 — Appendix S1. Distribution of matrotrophy among invertebrates and invertebrate chordates (proved, stated or inferred based on indirect evidence). [file BRV-91-673-s001.pdf]

Appendix S1. Distribution of matrotrophy among invertebrates and invertebrate chordates (proved, stated or inferred based on indirect evidence).

References containing histochemical and/or ultrastructural[1] and experimental[2] (autoradiographic labelling, calcium transfer, diet manipulation, dry mass and organic mass analysis, estimation of energetic content) evidence of matrotrophy are marked by superscript numbers. Original terminology, descriptive phrases and egg/embryo/larva/juvenile sizes are given in square brackets. Measurements (diameter or length×width) made from published illustrations are given in braces {}. Examples given in red are considered here as potentially matrotrophic. Symbols and abbreviations: ø, diameter; ER, endoplasmic reticulum; GER, granular endoplasmic reticulum; PAS, Periodic acid-Schiff staining; S.E.M., standard error of the mean; S.D., standard deviation.

Taxon	Matrotrophy (proved, recorded or suggested) in	Type of incubation and site of embryonic nutrition	Presumed method and structure(s)/organ(s) for nutrient delivery	Presumed mode and fetal organs for nutrient uptake	Reference
**<u>Phylum Porifera</u>**<br>**Class Calcarea**<br>**Family Sycettidae**	*Sycon raphanus* [oocyte 40.0×50.0 μm], {stomoblastula with a cavity 30.0×60.0 μm} (Duboscq & Tuzet, 1937)	Viviparity: embryos and larvae develop in mesohyl, each being surrounded by protective capsule [capsule protectrice] (Duboscq & Tuzet, 1937) also called [embryonic capsule] (Korotkova, 1981) formed from the cells of mesohyl and having no nutritional function; additionally, from the side of the nearest choanocyte chamber embryos are partially surrounded by placental membrane [membrane	[Membrane placentaire] is formed from modified choanocytes during formation of blastula (Duboscq & Tuzet, 1937); [transformed choanocytes] (Fell, 1989; Leys & Ereskovsky, 2006); [placental membrane] (Ereskovsky, 2010)	[Embryo obtains its nutrition through interaction of its granular cells with cells of placental membrane] (Ereskovsky, 2010: p. 14) (based on Duboscq & Tuzet (1937); Korotkova (1981) schematically depicted stages in the placental membrane formation: according to her images it is placed between embryonic capsule and blastula being in contact with granular cells (macromeres) during incurvation (nutrition method is unclear –	Duboscq & Tuzet (1937, 1938);<br>Korotkova (1981);<br>Fell (1989);<br>Ley & Ereskovsky (2006);<br>Ereskovsky (2010)

		placentaire].		intercellular transport?); also Korotkova wrote that during blastula formation a number of modified choanocytes that are used for embryonic development move to the larval cavity.	
	Sycon elegans		[Membrane nourricière].		Duboscq & Tuzet (1944) in Vacelet (1964)
**Family Grantiidae**	*Grantia compressa* [zygote ~40.0 µm, early stomoblastula (with a cavity) 280.0×450.0 µm] (Gallissian, 1983)	Viviparity: embryos and larvae develop in mesohyl, being surrounded by protective capsule [capsule protectrice] formed from the cells of mesohyl (Duboscq & Tuzet, 1937) and, partially, by [placental membrane].	[Nutrient capsule; capsule of nutrient squamous cells; larva is nourished by a special nutrient chamber formed of mesogleal cells] (Gatenby, 1920: pp. 266, 288); [nutritive membrane; there can be no doubt that these membranes form a sort of placenta between the mother sponge and the larvae the latter contains] (Gatenby & King, 1929: p. 319); [membrane placentaire] (Duboscq & Tuzet, 1933, 1934, 1937); [placental membrane] (Webb, 1935; Lufty, 1957); [membrane nourricière, membrane placentaire] is formed from modified choanocytes during	*Text modified during translation*: [during formation of placental membrane some cells are detached from it and phagocytozed by granular cells (macromeres), some others move inside the larval cavity; granular cells are changed during embryogenesis: in stomoblastula they are oval, about 8.0 µm, in amphiblastula just after incurvation they are irregular, about 15.0 µm, in mature amphiblastula they are pyramidal, about 20.0 µm. In both young and mature amphiblastulae granular cells phagocytoze cells of nutritive membrane either by whole or in pieces; before incurvation	Gatenby (1920); Gatenby & King (1929); Duboscq & Tuzet (1933, 1934, 1937); Webb (1935); Lufty (1957); Gallissian (1983); Fell (1989)

			incurvation of stomoblastula (Gallissian, 1983);<br>[placenta] (Fell, 1989); mechanism of [placental membrane formation] is the same as in *Sycon raphanus.*	both flagellate and granular larval cells contain abundant yolk granules inherited from the egg, whereas they disappear from the granular cells during incurvation. Thus, before incurvation larval development is based on the egg resouces, whereas the nourishment of the larva is provided by phagocytosis of the placental membrane cells after incurvation] (Gallissian, 1983: pp. 68-69, 70, 73)	
**Family Amphoriscidae**	*Paraleucilla magna* [Brasil: mature oocyte mean size 28±4.4 µm (up to 45.0 µm)] (S.E.M.) (Lanna & Klautau, 2010);<br>[Mediterranean: oocyte 37.0 µm, amphiblastula 62.33±12.38 µm] (S.E.M.)<br>(Longo *et al.*, 2012)	Viviparity: embryos and larvae develop in mesohyl, being surrounded by [continous sheath...of epilarval trophocyte epithelium] (Lanna & Klautau, 2012: p. 281)	[Epilarval trophocyte epithelium (ETEC): some choanocytes delaminated and became the presumptive trophocyte cells; while the embryo was inverting, the cells of the trophocyte plate proliferated and actively enveloped the whole embryo; amoeboid cells of maternal origin migrated into the larval cavity before it sealed its pore. These maternal amoeboid cells	[Presence of similar types of inclusion in choanocytes, ETECs, and granular cells …suggests the transference of these inclusions from choanocytes to the larva through the epilarval trophocyte epithelium. Besides this, the presence of small vesicles and some extruded cytoplasm material in the inner space of the epilarval trophocyte epithelium reinforces the possibility of a nutrient fow	Lanna & Klautau (2010, 2012);<br>Longo *et al*. (2012)

			apparently degenerated in the larval cavity, perhaps providing a nutritional reserve for the multiplication of the latter] (Lanna & Klautau, 2012: pp. 280–281)	between the larva and the epilarval trophocyte epithelium] (Lanna & Klautau, 2012: p. 288)	
**Family Leucosoleniidae**	*Leucosolenia complicata* {mature oocytes 70.0–80.0 µm; stomoblastula 60.0×120.0 µm} (Anakina, 1981, 1997; Anakina & Drozdov, 2000)	Viviparity: embryos develop in mesohyl being in contact with modified choanocytes whereas prelarvae are brooded in choanocyte chamber for some time, and its macromeres are in contact with transformed choanocytes too (but it is unknown if nutrition is provided at this stage).	[Nurse cells]=modified choanocytes (Anakina, 1981, 1997)	*Text modified during translation*: [nurse cells make a close contact with macromeres of the embryo, arbonhydrate granules are formed in them, and the same granules appear in the macromeres. Starting from the stomoblastula stage, the macromeres show more intense staining for proteins. All this evidences the direct transfer of nutrients from the nurse cells to the embryo; during incurvation some macromeres and nurse cells migrate to the blactocoel] (Anakina, 1981: pp. 56, 57);<br>[development of embryos provisioned by the nutrients delivered by the maternal body as a result of the interrelationships between	Anakina (1981, 1997); Korotkova (1981); Anakina & Drozdov (2000)

				macromeres and complex nurse cells (derived from choanocytes)] (Anakina, 1997: p. 47)	
**Family Petrobionidae**	*Petrobiona massiliana* {oocyte 80.0 µm} (Gallissian, 1981); {stomoblastula ~70.0×80.0 µm} (Vacelet, 1964)	Viviparity: early embryogenesis occurs in mesohyl;<br>and brooding: prelarva develops in modified choanocyte chamber.	*Text modified during translation*: [stomoblastula incurvates to the choanocyte chamber, and the entire chamber modifies for larval nutrition to 'capsule nourricière', thus larval nutrition begins after incurvation: some cells of the 'capsule nourricière' form pseudopodia towards granular cells, some cells of the capsule migrate to the larval cavity] (Vacelet, 1964: p. 64);<br>[The transformation of choanocytes into nurse cells begins with those choanocytes closest to the carrier cell, then spreads to the whole choanocyte chamber; nurse membrane is formed by the *in situ* transformation of choanocytes from the chamber where the embryo develops; the cytoplasm	*Text modified during translation*: [in stomoblastula size of granular cells is 15.0–20.0 µm, after incurvation 25.0–30.0 µm; the number of granular cells is four; after incurvation granular cells form numerous pseudopodia towards the cells of 'capsule nourricière'] (Vacelet, 1964: p. 67);<br>[phagocytosis of the nurse cell fragments enclosed in the embryo cavity (by) amoeboid cells of the posterior pole in the mature amphiblastula] (Gallissian & Vacelet, 1992: p. 140);<br>[young amphiblastulae phagocytosing modified choanocytes] (Gilis *et al*., 2011: p. 196)	Vacelet (1964);<br>Gallissian (1981);<br>Gallissian & Vacelet (1992);<br>Gilis *et al*. (2011)

			contains numerous inclusions: clear vesicles, lipid droplets, well-developed ergastoplasm, mitochondria, and numerous inclusions with heterogeneous structure. They often detach from the membrane and come into contact with pseudopodia released by the embryo cells] (Gallissian & Vacelet, 1992: pp. 137, 139, 140)		
**Family Clathrinidae**	*Guancha arnesenae*	Viviparity: embryos develop in mesohyl, in a [follicle] made of transformed choanocytes.	[Embryos develop inside a follicle], consisting of the internal layer of modified choanocytes and external collagenous lining. [The internal layer is made up of large cells, which may be cuboidal, prismatic or flattened… Cytoplasm of the follicular cell is foamy, about one-third of it being filled with electron-transparent vacuoles... It also contains numerous phagosomes … with heterogeneous content and a few lipid droplets] (Ereskovsky & Willenz, 2008: p. 178);		Ereskovsky & Willenz (2008);<br>A. V. Ereskovsky, unpublished data

			[follicle cells form projections towards the embryo; there is also extracellular material visible between embryo and follicle] (A. V. Ereskovsky, unpublished data)		
**Class Demospongiae Family Stylocordylidae**	*Stylocordyla borealis* [mature oocyte 300.0 µm, immature embryo 400.0–600.0 µm, mature embryo/young sponge 700.0 µm] (Sará *et al*., 2002)	Viviparity: embryos develop in mesohyl, being surrounded by a follicle of the 'nurse cells'of mesohylar origin, inherited from the oocyte.	[Immature embryo coated with a layer of nurse cells 100.0 µm thick; in mature embryo… layer of nurse cells can be slightly detached, reduced (about 30.0 µm thick) or even lacking] (Sará *et al*., 2002: p. 426)		Sará *et al*., (2002); Ereskovsky (2010)
**Family Thoosidae**	*Thoosa mismalolli* [oocytes 70.0–120.0 µm, late embryos 240.0–289.0 µm]	Viviparity: embryos develop in mesohyl, being surrounded by a [follicle-like envelope of flattened cells] (Bautista-Guerrero *et al*., 2010: p. 290)			Bautista-Guerrero *et al*. (2010)
	Alectona wallichii {oocyte ~90.0 µm}, [embryo 200.0–320.0 µm]	Viviparity: embryos develop in mesohyl, surrounded by a [thin outer envelope made up of very thin, elongated cells, and by a dense inner layer, up to 10 µm			Vacelet (1999)

		thick, made of collagen fascicles] (Vacelet, 1999: p. 631)			
**Family Spongiidae**	*Spongia officinalis* [oocytes up to 200.0 µm, mature parenchymella up to 600.0 µm along the main axis] (Baldacconi *et al*., 2007); [eggs 150.0–190.0 µm, morulae 156.0–564.0 µm, oval larvae before release 340.0×540.0 µm] (A.V. Ereskovsky, unpublished data)	Viviparity: embryos develop in mesohyl, [in a cavity … the wall of which were delimited by a monolayer of flattened (lentiform) cells] (Baldacconi *et al*., 2007: p. 972; A. V. Ereskovsky, unpublished data)			Baldacconi *et al*. (2007); Pérez *et al*. (2006); A. V. Ereskovsky, unpublished data
	Hippospongia lachne [zygote 300.0 µm, larvae 375.0–525.0× 335.0–485.0 µm] (Kaye, 1991)	Viviparity: embryos develop in mesohyl, being surrounded by a [single layer of flattened nurse cells] and non-cellular [embryonic membrane] (Kaye, 1991: p. 21)	[During cleavage of the zygote, umbilici (collagen-like connections) form between embryos and nurse cell layers and these function in the transfer of symbiotic bacteria and other mesohyl substances from the maternal parent to the embryo] (Kaye, 1991: p. 13)		Kaye (1988, 1991)
	Hippospongia communis [egg 160.0×210.0 µm, morula 230.0×300.0 µm, larvae before release 350.0×500.0	Viviparity: embryos develop in mesohyl, in [brood chambers] (Zarrouk *et al*., 2013: p. 5)			Zarrouk *et al*. (2013); A. V. Ereskovsky, unpublished data

	µm] (A.V. Ereskovsky, unpublished data); [morula 350.0 µm, (solid) parenchymella larva 446.58 ±22.37 µm] (S.E.M.) (Zarrouk *et al*., 2013)				
**Family Irciniidae**	*Sarcotragus spinosulus* [oocytes 200.0 µm, early solid stereoblastula 415.1±30.4 µm (site 1), 396.3±5.2 µm (site 2)] (S.E.M.)	Viviparity: embryos develop in mesohyl, being surrounded by [a monolayer of binucleolate flattened cells] (Mercurio *et al*., 2013: p. 4)	[Embryos were delimited by a monolayer of binucleolate flattened cells presenting a large amount of inclusions, mainly accumulated in the central region] (Mercurio *et al*., 2013: p. 4)		Mercurio *et al*. (2013)
**Family Thorectidae**	*Thorecta farlovii* [longer ø of the ovum 0.076 mm, embryo ø 'from about 3 mm to nearly 5 mm'] (Dendy, 1888: p. 350); [the unusual length of time during which the embryo remains within the mother Sponge, and great size to which it attains, necessitate some special arrangement whereby it can be nourished] (Dendy, 1888: p. 351)	Viviparity: embryos develop in mesohyl.	[Nutrient epithelial cells] (Dendy, 1888: p. 352)		Dendy (1888)
**Family Verticillitidae**	*Vaceletia crypta*	Viviparity: embryos	Nutritive membrane	Phagocytosis of	Vacelet (1979);

	[coeloblastula 220.0–300.0 µm (sometimes Up to 350.0 µm), morula ~350.0 µm, larva 340.0×160.0 µm] (Vacelet, 1979)	develop in mesohyl being surrounded by [nutritive membrane].	[membrane nourricière] consisting of multinucleate 'nutrient cells' with dense granules surrounds each embryo (Vacelet, 1979: p. 96)	symbiotic bacteria (Vacelet, 1979)	Ereskovsky (2010)
**Family Spongilidae**	*Ephydatia muelleri* {egg 170.0–200.0× 120.0–140.0 µm, morula 330.0×215.0 µm}	Viviparity: embryos develop in mesohyl being surrounded by a [follicular envelope].			Mukai (1989)
**Family Malawispongiidae**	*Ochridaspongia rotunda* {150.0×230.0 µm, prelarvae without a cavity 160.0×450.0 µm}	Viviparity: embryos develop in mesohyl being surrounded by a layer (follicle) of flattened cells.			Gilbert & Hadzisce (1977)
**Family Chalinidae**	*Haliclona aquaeductus* [egg ~200.0 µm, embryos and larvae ~280.0 µm]	Viviparity: embryos develop in mesohyl being surrounded by a follicle of flattened cells supposedly of mesohylar origin.			A. V. Ereskovsky, unpublished data
	Haliclona loosanofi [eggs ranging in size from 25.0×25.0 µm to 43.0×35.0 µm, embryos from 150.0×130.0 µm to 215.0×185.0 µm]	Viviparity: embryos develop in mesohyl being surrounded by a [thin follicular envelope consisting of flattened pinacocytes] inherited from the oocyte (Fell, 1976: p. 202)			Fell (1976)
	Chalinula ecbasis	Viviparity: embryos			Fell (1969)

	[oocyte ~140.0 µm], {early embryo ~190.0×230.0 µm, late embryo ~200.0×270.0 µm, larva ~180.0×290.0 µm}	develop in mesohyl being surrounded by [the oocyte follicle] (Fell, 1969: p. 137)			
**Family Tedaniidae**	*Tedania charcoti* [oocytes oval in shape, 0.1 by 0.07 mm, the embryo … oval and measuring 0.24 mm in longest ø, … the embryo increases considerably in size, becomes approximate-ly spherical, 0.35 by 0.32 mm] (Burton, 1932: pp. 360-361)	Viviparity: embryos develop in mesohyl, [lying in a capsule which is itself connected with the choanosomal tissues by radiating suspensoria] (Burton, 1932: pp. 360-361)	[In the earliest stages, the embryonic capsule is thick-walled, and entirely empty except for the ovum, but in its walls may be seen a number of granular cells, spherical or oval in form, and measuring, on an average, 0.01 mm across. There can be little doubt that these are nutritive cells. Immediately prior to the first cleavage … they become much more numerous and a few are seen migrating into the capsular cavity…, and as segmentation proceeds the cavity becomes partially filled with a granular mass…, derived presumably from the breaking down of these cells. After the second cleavage, the nutritive cells become appreciably less in the capsular wall] (Burton,		Burton (1932)

			1932: pp. 360-361)		
**Family Latrunculiidae**	*Latrunculia magnifica* [oocytes 393.0±91.0 µm, up to 570.0 µm, larvae up to 1200.0 µm (mean 868.0±144.0 µm)]	Viviparity: embryos develop in mesohyl being [encircled with a layer of cells, some of which secrete a collageneous coat of fibrils around the embryo] (Ilan, 1995: p. 310)			Ilan (1995)
**Family Myxillidae**	*Myxilla incrustans* {egg 160.0×165.0 µm, embryo ~300.0×325.0 µm}	Viviparity: embryos develop in mesohyl, [in a cavity of brood chamber, formed by a layer of flattened pinacocyte-like cells surrounded by a layer of collagen; these cells apparently originate from nucleolate amoebocytes] (Efremova *et al*., 1987: p. 259)			Efremova *et al*. (1987)
**Family Cladorhizidae**	*Asbestopluma occidentalis* [oocyte 24.0 µm], {pre-larva 138.4×107.6 µm}	Viviparity: embryos develop in mesohyl being surrounded by [follicle cells] of mesohylar origin.	[Follicle cells (nurse cells) extend… pseudopodia both toward the mesohyl, and inwards to contact the embryo] (Riesgo *et al*., 2007*b*: p. 621)		Riesgo *et al*. (2007*b*)
**Family Darwinellidae**	*Aplysilla sulfurea* [egg cells, 180.0×250.0 µm, embryos	Viviparity: embryos develop in mesohyl being surrounded by follicle			A. V. Ereskovsky, unpublished data

	230.0×280.0 µm]	cells.			
**Family Tetillidae**	*Craniella schmidtii* {morula 640.0–700.0×400.0 µm, young sponge inside of parent ~4.0 mm}	Viviparity: embryos develop in mesohyl.	[On the exterior of the embryo is a thin layer of structureless or finely granular stained material, which extends inwards between the cells, filling the interstices left by them] (Sollas, 1888: pp. 39-41)		Sollas (1888)
**Family Halichondriidae**	*Hymeniacidon perlevis* [egg 100.0–120.0 µm, larva 160.0–180.0 µm] (Bergquist *et al*., 1970); [oocyte 100.0 µm, steroblastula 200.0 µm, parenchymella 250.0 µm] (Gaino *et al*., 2010)	Viviparity: embryos develop in mesohyl being surrounded by a thin cell layer.			Bergquist *et al*. (1970); Gaino *et al*. (2010)
	Halichondria sp. {egg 158.3×114.5 µm}, [larva 275.0×150.0 µm]	Viviparity: embryos develop in mesohyl being surrounded by [follicular epithelium].			Fell & Jacob (1979)
	Halichondria panicea [oocyte 170.0×200.0 µm, prelarva 445.0×215.0 µm]	Viviparity: embryos develop in mesohyl; [during vitellogenesis oocytes were being gradually surrounded by aggregates of ameboid cells. These aggregates became a single layer of flat pinacocyte-like cells over mature oocytes and developing embryos]			Gerasimova & Ereskovsky (2007)

		(Gerasimova & Ereskovsky, 2007: p. 329)			
**Family Suberitidae**	*Terpios hoshinota* {oocyte 65.0 µm} (Hirose & Murakami, 2011); {embryo 297.1×274.2 µm} (Fang, 2011)	Viviparity: embryos develop in mesohyl being surrounded by a thin cell layer visible in illustrations.			Fang (2011); Hirose & Murakami (2011)
**Class Hexactinellida Family Farreidae**	*Farrea sollasi* {later oocyte ~70.0×130.0 µm, morula ~145.0×173.0 µm, prelarva I ~145.0×175.0 µm, late prelarva ~117.0×270.0 µm}	Viviparity: in mesohyl [the fertilized ova and embryos in various developmental stages... are lodged in a cavity lined with a single layer of endothelial-like cells] (Okada, 1928: p. 3)			Okada (1928)
**Class Homoscleromorpha Family Oscarellidae**	*Oscarella lobularis, Oscarella tuberculata* [oocytes 141.0–159.0 µm] (Ereskovsky & Boury-Esnault, 2002); [embryos 150.0-170.0 µm, prelarvae 130.0–200.0 µm] (Ereskovsky *et al*., 2013)	Viviparity: embryos develop in mesohyl being surrounded by a [follicle formed by endopinacocytes] (Ereskovsky & Boury-Esnault, 2002: p. 1765)			Ereskovsky & Boury-Esnault (2002); Ereskovsky *et al*. (2013)
	Oscarella nicolae [oocyte 100.0 µm, morulae 140.0 µm]	Viviparity: embryos develop in mesohyl being surrounded by trapezoid	Trapezoid ‘follicle’ cells produce a secretion ‘towards’ embryo.		A. V. Ereskovsky, unpublished data

		'follicle' cells.			
**Family Plakinidae**	*Corticium candelabrum* [oocytes 100.0–150.0 µm, free larvae 180.0–260.0 µm] (De Caralt *et al*., 2007); [oocytes 125.0–175.0 µm (up to 200.0–250.0 µm)] (Riesgo *et al*., 2007*a*)	Viviparity: in mesohyl, [embryos are surrounded by firmly interlaced follicular cells from the parental tissue] (De Caralt *et al*., 2007: p. 211); [a cellular follicle appeared to envelope the newly formed zygotes... We suspect that it formed rapidly from nurse cells trans-differentiating into flattened pseudo-epithelial cells...the follicle enclosed embryos tightly] (Riesgo *et al*., 2007*a*: p. 410)	[Cells at the basal part of the embryos contain phagocytosed bacteria] (De Caralt *et al*., 2007: p. 213); [neither phagocytosed bacteria nor symbiotic bacteria were observed within the cytoplasm of the larval cells] (Maldonado & Riesgo, 2008: p. 309)		De Caralt *et al*. (2007); Riesgo *et al*. (2007*a*); Maldonado & Riesgo (2008)
**Phylum Cnidaria**<br>**Class Scyphozoa**<br>**Family Pelagiidae**	*Chrysaora hysoscella* [egg 47.0 µm, planulae from 85.0×65.0 µm to 750.0×400.0 µm; planulae from 300 to 1000 times prevail the egg in volume] (Teissier, 1929: pp. 140, 149); [egg 0.13 mm] (Hadzi, 1907 in Berrill, 1949);	Viviparity : in ovary.	[La nutrition par le parent] (Teissier, 1929: p. 150); planulae are [nourished by the internal medium of the parent] (Berrill, 1949: p. 396, referring to Teissier, 1925)	[Absorption of nourishment from the internal medium of the parent] (Teissier, 1925: p. 531)	Teissier (1925, 1929); Berrill (1949)

	[blastula… grows to a size 7 to 10 times the linear dimension (ø) or 50 to 100 times the volume]<br>(Berrill, 1949: p. 396, referring to Teissier, 1925)				
**Family Ulmaridae**	*Stygiomedusa gigantea* [young scyphistoma 0.35 mm, largest capsula 6.5 cm long, 4.3 cm wide]	Brooding: in a [capsule] – protrusion of the stomach wall to a [brood chamber] beneath; embryo is additionally surrounded by a [cyst or chorion].	[Cyst wall acts as a chorion for the scyphistoma and draws its nourishment directly from the (stomach) lumen of the parent by means of its tube-like projections]<br>(Russell & Rees, 1960: p. 316)		Russell & Rees (1960)
**Class Hydrozoa Family Rhopalonematidae**	*Crossota millsae* {egg/early embryo 1.5–1.6 mm in ø}, [juveniles 5.0 mm ø; juveniles reach almost 6.0 mm in length before separation from the mother]<br>(Thuesen, 2003: pp. 4, 8)	Viviparity: early embryogenesis occurs in ovary;<br>and brooding: later juveniles burst out being suspended underneath a subumbrella.		Juveniles [gain nutrients from the mother]<br>(Thuesen, 2003: p. 10)	Thuesen (2003)
**Class Anthozoa Family Acroporidae**	*Acropora cuneata* [mean ø oocytes 255±26.2 µm,	Viviparity: in tissues [within an envelope of mesoglea and	[The embryos, … encompassed by mesenterial tissue,		Kojis (1986)

	maximal ø 325.0 µm, mean larval ø 1687±280 µm] (S.D.)	gastrodermis remaining attached to the mesentery by a stalk until the larvae matured] (Kojis, 1986: p. 291)	remained connected to the mesentery while developing in the coelenteron. As the embryos increased in size, they filled the coelenteron and were deeply indented by the projecting septa. …Polyp's mesenteries adhered firmly to the mesenterial envelope surrounding the large planulae suggesting that an exchange of nutrients might be occurring] (Kojis, 1986: p. 294)		
	Acropora palifera [mean ø oocytes 249±38.8 µm, maximal ø 325.0 µm, mean larval ø 1459±180 µm] (S.D.)	Viviparity: in tissues [within an envelope of mesoglea and gastrodermis remaining attached to the mesentery by a stalk until the larvae matured] (Kojis, 1986: p. 291)	As above.		Kojis (1986)
**<u>Phylum Ctenophora</u>** **Family Lyroctenidae**	*Lyrocteis imperatoris* [egg and gastrula 0.5 mm in ø, late embryo 1.5 mm in ø and more]	Viviparity: in [brood chamber …expansion of the terminal end of the ovarian diverticulum], its wall is [extremely thin (cell) membrane] (Komai, 1942: p. 22)			Komai (1942); Pianka (1974)

**<u>Phylum Platyhelminthes</u> Class Rhabditophora Family Typhloplanidae**	*Mesostoma ehrenbergii* [subitaneous embryonated egg with few blastomeres and peripheral cells, 100.0 µm; with continuing embryonic development, the S egg increased rapidly in size] (Domenici & Gremigni, 1977: p. 249)	Viviparity: in uterus.	Uterine cells with microvilli; [parentally derived substances are transferred to developing embryo] (Gremigni & Domenici, 1977: p. 263)	[Peripheral cells] (of parental origin) with microvilli; [uptake of paternal proteins by… pinocytosis] (Gremigni & Domenici, 1977: p. 263)	Bresslau (1904);<br>Fiore & Ioalè (1973[2]);<br>Gremigni & Domenici (1976[2], 1977[1,2]);<br>Domenici & Gremigni (1977[1]);<br>Cable *et al*. (1996, 1997)
	Mesostoma lingua	Viviparity: in uterus.		Oophagy.	Heitkamp (1972)
	Bothromesostoma essenii [summer eggs 0.18–0.2 mm, juveniles 198.0–230.0×500.0–646.0 µm] (J. V. Korneva, unpublished data); [embryos developing from summer eggs differ in size] (Mamkaev *et al*., 2014: p. 2)	Viviparity: on early stages in parenchyma, on later stages in uterus.	Uterine cells with microvilli.	Embryonic epithelial cells with microvilli.	Mamkaev *et al.* (2014);<br>J. V. Korneva, unpublished data
**Family Graffillidae**	*Paravortex cardii* [embryos grow] (MacKinnon *et al*., 1981: p. 246)	Viviparity: in parenchyma =[mesenchyme] [thin-walled capsules usually contain…two	Presumed [transfer of soluble and particulate nutriments from the parental gut] *via* intact embryonic	[Developing embryo utilizes the lipid reserves of the yolk globules… There was some evidence	MacKinnon *et al*. (1981);<br>Jennings & Phillips (1978[1]);<br>Jennings (1981)

		developing embryos and a mass of yolk cells] (Jennings & Phillips, 1978: p. 544)	[capsule wall]… (for) [supplementary nourishment of the embryos] (Jennings & Phillips, 1978: p. 552)	that once the yolk reserves are exhausted, and before the embryos leave their capsule, nutrients are passed from the parental gut through the (intact) capsule (wall) to the embryos] (Jennings & Phillips, 1978: p. 551)	
	Paravortex karlingi {embryonic increase is visible in Figs. 3 and 10} (Pike & Burt, 1981); [embryos grow] (MacKinnon *et al*., 1981: p. 246)	Viviparity: in parenchyma.			Pike & Burt (1981); MacKinnon *et al*. (1981)
	Paravortex scrobiculariae	Viviparity: in parenchyma (as in *Paravortex cardii*).		As in *Paravortex cardii*	Jennings & Phillips (1978[1]); Jennings (1981)
	Paravortex panopea {early embryo 100.0×61.0 µm, fully developed embryo 138.8×88.8 µm}	Viviparity: in parenchyma.			Brusa *et al*. (2011)
	Paravortex gemellipara	Viviparity: in parenchyma.		[Uptake of free yolk globules by wandering amoeboid ectoderm and primary entoderm cells in gastrulae and post-gastrulae] (Jennings & Phillips, 1978: p. 559)	Ball (1916) in Jennings & Phillips (1978)

**Class Monogenea**<br>**Family Gyrodactylidae**	446 species (almost entire family except 4 genera);<br>examples:<br>*Gyrodactylus bullatarudis, Gyrodactylus gasterostei*,	Viviparity: in uterus.	[Embryo nutrition is a function of the mother *via* uterus] (Cable & Harris, 2002: p. 263);<br>[syncytial… uterus lining of gyrodactylids is metabolically active, and responsible for transfer of nutrients to developing embryos]<br>(Cable *et al*., 1996: p. 524)	[Fluid-filled uterus (is) a medium by which embryos could take up nutrients across their entire surface] (Braun, 1966 in Cable & Harris, 2002: p. 263)	Cable *et al*. (1996);<br>Jones *et al*. (1998);<br>Braun (1966) in Cable & Harris (2002);<br>Boeger *et al*. (2003);<br>Bakke *et al*. (2007)
	Gyrodactylus turnbulli when [embryo has almost attained its full complement of cells, … (it) has grown to a size five to ten times that of the oocytes] (Cable *et al*., 1996: p. 524)	Viviparity: in uterus.			Cable *et al*. (1996);
	Macrogyrodactylus polypteri<br>[egg 200.0–300.0 μm]	Viviparity: in uterus.	[…Process of nutrient transfer occurs across each of the series of interfaces between parent with embryo, and embryo with embryo. … Provision of nutrients from the mother presumably becomes increasingly important as intrinsic stores of the oocyte are depleted] (Cable *et al*., 1996: p. 524)		Cable *et al*. (1996)
**Family**					

**Polystomatidae**	*Pseudodiplorchis americanus* smallest–largest size of [cell mass 83.0×148.0–234.0×408.0 µm, fully developed larva 185.0×537.0–297.0×574.0 µm] (Cable & Tinsley, 1991)	Viviparity: in uterus.	[Fine cytoplasmic strands; luminal cytoplasmic extensions of the uterus] (Cable & Tinsley, 1991: p. 257); [direct transfer of nutrients from parent to offspring] (Cable & Tinsley, 1991: p. 253); [almost complete dependence on uterine provision of nutrients] (Cable *et al*., 1997: p. 1079)	Egg [capsule lining… extends into the cytoplasmic processes which meander across the lumen and attach to the larval tegument between ciliated cells] (Cable & Tinsley, 1991: p. 257); [these connexions are packed with glycogen and appear to perform a placenta-like function] (Cable & Tinsley, 1991: p. 253)	Cable & Tinsley (1991[1]); Cable *et al*. (1996, 1997); Tinsley (1983)
	Neodiplorchis scaphiopodis [the capsule is a thin flexible sac which invests the newly formed embryo yet expands to accommodate an extremely large infective stage, up to 600 µm in length; the egg capsule ø of fully formed larvae is about 8 times longer than the ø of capsules surrounding recently developed embryos] (Cable & Tinsley, 1991: pp. 254, 255); [egg capsule is only 60 µm in ø, but as it passes	Viviparity: in uterus.	Uterine wall.	[Cytoplasmic lining of the egg-capsule] and its cytoplasmic connexions; …primary function of the cytoplasmic connexions… (is) the transfer of nutrients to the embryo] (Cable & Tinsley, 1991: p. 261)	Cable & Tinsley (1991[1]); Cable *et al*. (1997); Tinsley (1983)

	to the distal uterus it expands to 800 µm in ø to accommodate the growing larva] (Cable *et al*., 1997: p. 1075)				
	Parapolystoma crooki [eggs dissected from large, gravid adults showed various degrees of embryonation and increase in size with development from an average of 52 by 31 to one of 60 by 38 (µm)] (Vande Vusse, 1976: p. 554)	Viviparity: in uterus.			Vande Vusse (1976); Tinsley (1983)
	Eupolystoma anterorchis [eggs developing *in utero* to the point of hatching; capsules... increasing in size during development from 0.135×0.080 to 0.185×0.105 (µm)] (Tinsley, 1978: p. 294); [in a flexed, doubled-up position within their capsules… the fully developed oncomiracidium… (350 µm long) is confined inside a	Viviparity: in uterus.			Tinsley (1978, 1983)

	capsule little more than half its length (185 µm)] (Tinsley, 1983: p. 166)				
**Class Digenea**<br><br><br>**Family Schistosomatidae**	In parthenits of all ~18,000 species, and in adult worms of some species;<br>examples:<br>*Schistosoma japonicum*	Viviparity: in parthenit body cavity – pseudocoel.<br><br><br>Viviparity: in parthenit body cavity – pseudocoel.	Tegument of daughter sporocyst, redial wall; [nutrients might be taken in from the snail haemolymph, by way of connections with the syncytial tegument of the daughter sporocyst, and pass into the brood chamber where the cercarial embryo obtains the nutrients by way primitive epithelium] (Göbel & Pan, 1985: p. 238)	[Primitive epithelium of cercaria; spherical and rod-shaped bodies situated in the matrix of the fully developed tegument were also observed in canals which pass from the syncytial and muscular layer to the subtegument of the cercaria. We postulate that these structures also function in the transport of nutrients] (Göbel & Pan, 1985: pp. 238-239)	Cable *et al*. (1996); Galaktionov & Dobrovolskij (2003)<br><br><br>Göbel & Pan (1985[1])
	Schistosoma mansoni	Viviparity: in parthenit body cavity – pseudocoel.		[Primitive epithelium (transitory envelope) of daughter sporocyst… probably… has a function of nutrition] (Meuleman *et al*., 1980: p. 205)	Meuleman *et al*. (1980)
**Family Heterophyidae**	*Cryptocotyle lingua*	Viviparity: in parthenit body cavity – pseudocoel.		Primitive epithelium, [transmission of metabolites from the redial lumen and	Rees & Day (1976)

				the passage outwards of excretory products] (Rees & Day 1976: p. 316); epidermal cells of cercaria; [absorption of nutrients from the redial lumen or by direct transfer from cells of the redial wall] (Rees & Day 1976: p. 318)	
**Family Hemiuridae**	In maritae of some species, including *Halipegus eccentricus*	Viviparity: in uterus.<br><br>Viviparity: in uterus.	[Embryo nutrition is provided, during development, by the parent] (Holy & Wittrock, 1982, in Tinsley, 1983: p. 170)	[Narrow channels in egg shell presumably permit passage of nutrients to embryo from glycogen-rich parenchyma adjacent to uterine epithelium] (Holy & Wittrock, 1982, in Tinsley, 1983: p. 170)	Holy & Wittrock (1986); Cable *et al*. (1996); Holy & Wittrock (1982) in Tinsley (1983)
**Family Plagiorchiidae**	*Haematoloechus medioplexus*	Viviparity: in uterus.		[Uptake of glucose] by developing embryos in egg shell (Burton, 1962: p. 881)	Burton (1962²); Tinsley (1983)
**Family Philophthalmidae**	*Philophthalmus megalurus* [eggs… increase in size as they traverse the uterus] (Nollen, 1968: p. 302)	Viviparity: in uterus.	[As the embryo develops and increases in size the resultant stretching of the thin, elastic shell may facilitate entry of substances from the uterine fluid… Thus it seems that in trematodes whose eggs develop appreciably before they are	[Entry and incorporation of thymidine, glucose, tyrosine, and leucine in developing miracidia] (Nollen, 1968: p. 295)	Nollen (1968²); Tinsley (1983)

			laid, the uterus…may well provide the eggs with a variety of nutrients beyond any supplied by the vitelline cells] (Nollen, 1968: p. 303)		
	Parorchis acanthus [egg capsule increases from 90.0×35.0 to 130.0×68.0 µm during passage along the uterus] (Rees, 1940: p. 379 in Tinsley, 1983: p. 172)	Viviparity: in uterus.			Rees (1940) in Tinsley (1983)
**Family Heronimidae**	*Heronimus chelydrae* [uterine eggs average 256.0×164.0 µm, miracidium maximum length of half a millimetre; thin membranous (egg) shell enlarges as the miracidium grows; the more advanced the stage of development of the miracidium, the larger the egg] (Lynch, 1933: p. 13)	Viviparity: in uterus.			Lynch (1933)
**Class Cestoda Family Nippotaeniidae**	*Nippotaenia mogurndae* [in the early gravid uterus … eggs' (with early embryo) size	Viviparity: in uterus.	[Placental-like interaction] (Davydov & Korneva, 2000: p. 77); [uterine epithelium …	[Small knobs and long thin lamellae located on the surface of developing (embryonated) eggs]	Davydov & Korneva (2000[1]); Korneva (2007[1]); Korneva *et al*. (2014[1])

	averages 7.0–12.0 µm]; embryonated [eggs have a maximum diameter of 30 µm in gravid proglottids] (Korneva *et al*., 2014: pp. 427–428)		forms numerous diverticula. Numerous cytoplasmic microlamellae of the uterine epithelium intimately interlace with the thin and delicate outer envelope surrounding the hexacanth larva] (Korneva *et al*., 2014: p. 427)	(Korneva *et al*., 2014: p. 427)	
**Family Proteocephalidae**	*Proteocephalus thymalli*	Viviparity: in uterus.	[Placental type interaction between occur outgrowths of uterine epithelium and thin capsule of embryos] (Korneva, 2005: p. 552)		Korneva (2005[1], 2007)
	Proteocephalus torulosus	Viviparity: in uterus.	As above.		Korneva (2005[1], 2007[1])
**Family Bothriocephalidae**	*Clestobothrium acheilognathi*	Viviparity: in uterus.	[Placental type interaction …are limited in time and space; multiple epithelial lamellar outgrowths] of uterine wall] (Korneva, 2005: pp. 552-553)		Korneva (2005[1], 2007[1])
**Family Hymenolepididae**	*Hymenolepis diminuta*	Viviparity: in uterus.	[Autoradiographs… demonstrate that at least a portion of the 14C-labelled lipid is transported to the eggs and incorporated therein] (King & Lumsden, 1969: p. 259)		King & Lumsden (1969[2]); Conn (1993)
	Ditestolepis diaphana	Viviparity: in uterus.	Uterine epithelium;		Korneva *et al*. (2010[1]);

	[early embryos 12.0 µm, hexacanths in embryonated eggs 20.0–24.0 µm] (J. V. Korneva, personal communication 2013)		[interactions of placental type] (Korneva *et al*., 2010: p. 1181)		Korneva & Kornienko (2013a[1])<br>J. V. Korneva, personal communication 2013
	Skrjabinacanthus diplocoronatus<br>[early embryos 8.0–10.0 µm, hexacanths in embryonated eggs 20.0–21.0 µm] (J. V. Korneva, personal communication 2013)	Viviparity: in uterus.	[Columnar cords of syncitial uterus] (Korneva *et al*., 2012: p. 1525)		Korneva *et al*. (2012[1]); Korneva & Kornienko (2013a[1])
	Urocystis prolifer<br>[early embryos 11.0–16.7 µm, hexacanths in embryonated eggs 11.0–18.5 µm] (J. V. Korneva, personal communication 2013) (incipient matrotrophy?)	Viviparity: in uterus.	[In gravid proglottids, the uterine epithelium becomes a substantially thicker layer than in earlier stages. Apical microlamellae of the epithelium generally extend into the lumen parallel to the uterine surface... Much of the cytoplasm contains extensive GER, which consists of moderately dilated cisternae containing electron-transparent material] (Korneva *et al*., 2012: p. 1524)		Korneva *et al*. (2012[1]); Korneva & Kornienko (2013a[1]): authors suggested no nutrient transfer;<br>J. V. Korneva, personal communication 2013
	Microsomacanthus paracompressa	Viviparity: in uterus.	Cytoplasmic projections of the uterine wall [=uterine	[Folded outermost envelope of oncosphere;	Chomicz (1996[1])

	{intrauterine eggs 8.8–22.2 µm} (Chomicz, 1996: figs. 4-5)		envelope];<br>[participation of the uterus in uterus-egg exchange] (Chomicz, 1996: p. 198)	condensation of electron-dense material on] the outer embryonic envelope, (Chomicz, 1996: p. 195)	
	Diorchis parvogenitalis	Viviparity: in uterus.	As above.	As above.	Chomicz (1996[1])
	Fimbriaria fasciolaris {intrauterine eggs 16.0–16.8 µm} (Chomicz, 1996: figs. 1, 3)	Viviparity: in uterus.	[Ultrastructure of uterine envelope reflects a high level of protein synthesis and suggests the importance of (its) immense surface…for inter-envelope exchange] (Chomicz & Czubaj, 1991: p. 506);<br>[cytoplasmic layer forming uterine envelope; participation of the uterus in uterus-egg exchange] (Chomicz, 1996: pp. 195, 198)	As above.	Chomicz & Czubaj (1991[1]);<br>Chomicz (1996[1])
	Fimbriaria czaplinskii {intrauterine eggs 44.3–90.0 µm} (Chomicz, 1996: figs. 8-9)	Viviparity: in uterus.	Cytoplasmic projections of the uterine wall;<br>[participation of the uterus in uterus-egg exchange] (Chomicz, 1996: p. 198)	As above.	Chomicz (1996[1])
	Arostrilepis tenuicirrosa [eggs' size averages 30 µm in early stages (of embryonic development); in gravid proglottids… the eggs have a maximum diameter of 46 µm] (Korneva *et al*., 2014:	Viviparity: in uterus.	Uterine epithelium forms [epithelio-mesenchymal …long and thick diverticula…filled with lipid droplets; extracellular matrix concentrated into dense aggregations, which settles among the eggs]		Korneva *et al*. (2014[1])

	p. 428)		(Korneva *et al*., 2014: p. 428)		
	Lineolepis scutigera [early embryos 10.0 µm, embryonated eggs measured without a shell 20.0–25.0 µm on average] (J.V. Korneva, personal communication 2014)	Viviparity: in uterus.	Uterine epithelium.		Korneva & Kornienko (2013b[1])
**Family Dilepididae**	*Monocercus arionis* embryonated [eggs' size averages 10–12 µm in…early stages; in gravid proglottids… eggs have a maximum size of 40 µm] (Korneva *et al*., 2014: p. 428)	Viviparity: in uterus.	[Uterine epithelium (forms) bulges (with) lipid drops; epithelial layer (of) uterus contains…large stacks of cisternae of ER], (Korneva *et al*., 2011: p. 23); [uterine wall forms a few epithelio-mesenchymal diverticula] (Korneva *et al*., 2014: p. 428); [supplying developing eggs with nutrients through the thin capsule occurs…upon the direct contact of eggs with the uterine wall] (Korneva *et al*., 2011: p. 26)	[Abundant fibrils, appear throughout the (uterine) lumen, connecting the outer envelopes of adjacent eggs and eggs with nearby uterine diverticula. The fibrils vary in thickness… and are strongly interwoven among themselves… The quantity of fibrils forming a network, which involves all eggs in the uterus, becomes more prominent and abundant as the proglottids mature] (Korneva *et al*., 2014: pp. 428-429)	Korneva *et al*. (2011[1], 2014[1]); Korneva & Kornienko (2013a[1])
**Family Tetrabothriidae**	*Tetrabothrius erostris* [in mature proglottids (early embryonated) eggs' size averages	Viviparity: in uterus.	[Uterine epithelium in mature proglottids extends fungiform papillae] (Korneva *et al*., 2014: p.		Korneva *et al*. (2014[1])

	20.0 µm; in gravid proglottids…the eggs have a maximum diameter of 40–50 µm] (Korneva *et al*., 2014: p. 427)		427)		
**Family Nematotaeniidae**	*Cylindrotaenia hickmani* {early embryo without envelopes 10.1×6.0 µm, fully developed embryo without envelopes 13.0×8.5 µm}	Viviparity: in uterus.	[Uterine envelope; nourishment of developing eggs, or the provision of materials for the various embryonic envelopes may be postulated] (Jones, 1988: p. 557)		Jones (1988[1])
**Family Linstowiidae**	*Oochoristica anolis*	Viviparity: in uterus.	[Egg capsules…consist of syncytial uterine epithelium, with material that appears to be its secretory product deposited in the lumen; [packing of egg capsules in tissue rich in lipid droplets (plays)…possible role in oncosphere nutrition] (Conn & Etges, 1984: pp. 769, 777)		Conn & Etges (1984[1])
**Phylum Gastrotricha** **Family Macrodasyidae**	*Urodasys viviparus* [morula 90.0×50.0 µm, juvenile 120.0×80.0 µm] (M. A. Todaro, personal communication	Viviparity: in uterus.			Wilke (1954); M. A. Todaro, personal communication 2010; *http://biogeodb.stri.si.edu/* bioinformatics/dfm/metas/

	2010)				view/44266
**<u>Phylum Rotifera</u>**<br>**Class Monogononta**<br>**Family Asplanchnidae**	*Asplanchna sieboldi* […females fed α-tocopherol have an internal environment which stimulates their embryos to undergo additional growth during their development *in utero*. This tocopherol-mediated growth is due both to an increased number of mitotic divisions and also to an enlargement of the cytoplasm] (Gilbert, 1974: pp. 1009, 1989)	Viviparity: in uterus.			Gilbert (1974[2])
**<u>Phylum Acanthocephala</u>**<br>**Class Archiacanthocephala**<br>**Family Moniliformidae**	*Moniliformis moniliformis* {early embryo 65.7×26.3 µm, acanthor 89.4×34.2 µm} (Nicholas, 1967); {early embryo 56.0×14.0 µm,	Viviparity: in pseudocoel.			Nicholas (1967); Marshall *et al*. (1973)

	advanced embryo 68.0×24.0 µm} (Marshall *et al*., 1973)				
**Class Palaeacanthocephala Family Polymorphidae**	*Polymorphus minutus* [unfertilized egg 40.0×12.0 µm, acanthor 45.0×15.0 µm] {fertilized ovum 33.3×10.6 µm, acanthor 53.0×16.0 µm}	Viviparity: in pseudocoel.			Nicholas & Hynes (1963)
**Class Eoacanthocephala Family Neoechinorhynchidae**	*Neoechinorhynchus salmonis* [oocytes 5.0–7.0 µm, embryos 15.0×8.0–10.0 µm]	Viviparity: in pseudocoel.			E. I. Mikhaylova, personal communication 2013
	Neoechinorhynchus beringianus {oocytes 11.2 µm, early embryos 12.3–20.0×8.2–11.7 µm, acanthor 32.9×10.5 µm}	Viviparity: in pseudocoel.			E. I. Mikhaylova, unpublished photos
	Neoechinorhynchus tumidus {oocytes 10.5×10.2 µm, acanthors 30.2×10.5 µm}	Viviparity: in pseudocoel.			V. P. Nikishin, unpublished photos

**<u>Phylum Mollusca</u>**<br>**Class Bivalvia**<br>**Family Corbiculidae**	*Corbicula madagascarensis*<br>[shell length of brooded juveniles from 0.5 mm to 2–3 mm]	Brooding: among ctenidial lamellae.	[Truly euviviparous strategy, matrotrophy, maternal nourishment]<br>(Glaubrecht *et al*., 2006: p. 645)		Glaubrecht *et al*. (2006)
	Corbicula fluminea<br>[progressive increase in the mean length of the developing larva from 150 µm as a post fertilized ovum to 200–220 µm at the time of release as a D stage larva]<br>(Morton, 1977: p. 37);<br>[oocyte 140.0 µm, blastula 175.0 µm, gastrula 175.0–180.0 µm, veliger 190.0–250.0 µm, pediveliger with straight-hinged valves 230.0 µm in length, later straight-hinged juvenile shell valve 240.0 µm]<br>(Kraemer & Galloway, 1986)	Viviparity: in case of self-fertilization early embryos develop in oogenic follicles further being obviously moved to demibranchs;<br>and brooding: in the inner, marsupial gills<br>(Kraemer & Galloway, 1986: p. 68)	[Mucous cells in interlamellar junctions of ctenidia…may nourish the developing larvae]<br>(Morton, 1977: p. 37)	[Production of fecal material] in rectum;<br>[straight-hinded juveniles are capable of feeding while still in the parental gill cavity] (Kraemer & Galloway, 1986: pp. 71-72)	Morton (1977);<br>Kraemer & Galloway (1986)
	Corbicula australis<br>[fully grown oocytes 125.0 µm, released	Brooding: in interlamellar spaces of inner demibranchs.	Mucous cells in epithelium of interlamellar septa of enlarged inner demibranchs	[Microvilli-rich surface of velum of veliger…is characteristic of	Byrne *et al*. (2000)

	juveniles 250.0 µm shell length]		[nutrient uptake in marsupial environment; exogenous nutrients provided by parent] (Byrne *et al*., 2000: pp. 185, 195)	absorptive epithelium] (Byrne *et al*., 2000: p. 195)	
	Corbicula moltkiana [final size of larvae 0.35–0.40 mm (occasionally up to 0.56 mm)]	Brooding: in water tubes formed by interlamellar septae of inner demibranchs.	Interlamellar septae [thickened along their entire length] and consisting of high cylindrical cells; assumption of availability of nourishment, limited role of matrotrophy] (Korniushin & Glaubrecht, 2003: pp. 298, 302)		Korniushin & Glaubrecht (2003)
	Corbicula matannensis [final size of larvae 0.33–0.42 mm, juveniles occasionally up to 0.5 mm]	Brooding: in inner and, occasionally, in both inner and outer demibranchs.	Voluminous mucous cells in interlamellar septae and interfilament junctions; thickened interlamellar septa of inner demibranchs; [assumption of availability of nourishment, limited role of matrotrophy] (Korniushin & Glaubrecht, 2003: p. 302)		Korniushin & Glaubrecht (2003)
	Corbicula possoensis [final size of larvae 0.25–0.30 mm long]	Brooding: in water tubes formed by interlamellar septae of both outer and inner demibranchs.	Thickened interlamellar septae and interfilamentar junctions (of both inner and outer demibranchs) with voluminous mucous cells; [embryonic nourishment] (Korniushin & Glaubrecht, 2003: p. 304)		Korniushin & Glaubrecht (2003)

	Corbicula linduensis [small larvae 0.27 mm, final larval shell length up to 1.5 mm] (Korniushin & Glaubrecht, 2003)	Brooding: in water tubes of two inner demibranchs.	[Specialized nourishing tissue]: cylindrical epithelial and mucous cells in interlamellar septae; thickening of entire inner demibranchs; [tight contact between epithelium of septae and incubated juveniles, cylindrical epithelial cells were noticeable in this area; provision of nourishment other than yolk; several indications that matrotrophy might play a more important role in the larval development of this taxon] (Korniushin & Glaubrecht, 2003: pp. 304, 305, 307)		Korniushin & Glaubrecht (2003); Glaubrecht *et al*. (2006)
**Family Sphaeriidae**	*Musculium partumeium* [marsupial young under 0.6 mm in length very rarely had a definite shell; smallest marsupial young 0.585–0.668 mm, largest1.3–1.5 mm] (Van Cleave *et al*., 1947); [prodissoconch 0.17-<1.0 mm] (Heard, 1977); [intramarsupial stages: cellular "ball" embryo	Brooding: in marsupial sacs – outgrowths of gill filaments.			Van Cleave *et al*. (1947); Heard (1977); Hornbach *et al*. (1980)

	0.1 mm, fetal larvae 0.2–0.4 mm, prodissoconch larvae 0.5–1.1 mm, extra-marsupial larvae ≥1.2 mm; mean size at birth 1.4 mm with the upper limit 1.8 mm] (S.D.) (Hornbach *et al*., 1980)				
	Musculium secures [prodissoconch <1.0 mm] (Heard, 1977)	Brooding: in brood sacs – outgrowths of gill filaments.			Mackie *et al*. (1974); Heard (1977)
	Musculium lacustre [prodissoconch <1.0 mm] (Heard, 1977)	Brooding: in brood sacs – outgrowths of descending lamella of anterior inner demibranchs.			Heard (1977); Hetzel (1993) in Korniushin & Glaubrecht (2003)
	Musculium transversum [prodissoconch <1.2 mm]	Brooding: in brood sacs – outgrowths of descending lamella of anterior inner demibranchs.			Heard (1977)
	Musculium sp.	Brooding: in incubation sacs of internal hemibranchs.			Ituarte (2009)
	Sphaerium solidum	Brooding: in marsupial pouches.			Foster (1932)
	Sphaerium corneum [prodissoconch <2.0–3.02 mm, smallest extramarsupial larva 1.13 mm] (Heard, 1977)	Brooding: in brood sacs of inner gill.	Glandular cells of the internal wall of the brood sac; [embryonic nutrition] (Korniushin & Glaubrecht, 2003: p. 296)	Absorption of the secretions of glandular cells and ingestion of glandular cells by embryos.	Leydig (1855); Stepanoff (1865); Ziegler (1885); De Bruyne (1898); Poyarkoff (1910); Wasserloos (1911);

					Schereschewsky (1911);<br>Couteaux-Bargeton (1948)<br>Heard (1977);<br>Hetzel (1993) in Korniushin & Glaubrecht (2003)
	Sphaerium rivicola<br>[egg 50.0 µm, length of intramarsupial embryos from <1.0 mm to 6.0 mm] (Groenewegen, 1926);<br>[longest prodissoconch >4.0 mm]<br>(Heard, 1977)	Brooding: in brood sacs of inner gill.	[Nourishment of embryos] (Groenewegen, 1926: p. 269)		Groenewegen (1926);<br>Heard (1977);<br>Hetzel (1993) in Korniushin & Glaubrecht (2003)
	Schaerium japonicum	Brooding: in marsupial sacs on the anterior part of descending filaments of inner demibranchs.	[Nourishment of embryos]: (1) [secretory action] of inner epithelial layer of secondary and tertiary marsupial sacs – for marsupial embryos; (2) [nutriment layer of the inner branchial chamber] – for extra-marsupial embryos<br>(Okada, 1935: pp. 387-389)		Okada (1935)
	Sphaerium simile<br>[size of young 0.5–8.0 mm] (Gilmore, 1917);<br>[ovarian oocytes from 35.0×28.0 µm to 58.0×46.0 µm, embryos 0.3 mm,	Brooding: in brood pouches in inner gills.	[Very thick glandular cells] in the inner wall of brood pouch (Gilmour, 1917: p. 22);<br>[undoubted evidence of secretion in the brood pouch]		Gilmore (1917);<br>Zumoff (1973)

	juveniles 3.0–7.0 mm, newborn young 8.0 mm] (Zumoff, 1973); [embryos grow and develop] (Zumoff, 1973: p. 222)		(Gilmore, 1917: p. 23)		
	Sphaerium notatum {mature ovum 53.0×28.0 µm, embryo 0.64×54.0 µm}	Brooding: in brood pouches formed in upper parts of inner lamellae of inner gills.			Monk (1928)
	Sphaerium striatinum [oocytes 100.0 µm ø, intra-marsupial offspring ~3.0 mm shell length, extra-marsupial offspring 4.0 mm shell length (up to 4.5 mm)] (Beekey *et al*., 2000; Beekey & Hornbach, 2004); [general prodissoconch length 2.0 mm] (Heard, 1977)	Brooding: in marsupial sacs in both inner demibranchs.	[Development of intra-marsupial offspring is fueled solely by yolk and dissolved organics in the adult hemolymph] (Beekey *et al*., 2000: p. 1700)	[Ability of retained offspring to ingest particles; particles were only present in extra-marsupial offspring] (Beekey *et al*., 2000: p. 1698)	Heard (1977); Beekey *et al*. (2000); Beekey & Hornbach (2004)
	Sphaerium fabale [early prodissoconch 0.19–0.36 mm, general prodissoconch length 2.6 mm]	Brooding: [in brood sacs in the anterior, inner demibranchs] (Heard, 1977: p. 421)			Heard (1977)
	Sphaerium rhomboideum [general prodissoconch length 2.8 mm]	Brooding: [in brood sacs in the anterior, inner demibranchs] (Heard, 1977: p. 421)			Heard (1977)
	Pisidium lilljeborgii [egg 0.04 mm, early	Brooding: in brood sacs formed by inner	[Sac tissue (is used) for nutrition] (Meier-Brook,		Meier-Brook (1970, 1977)

	embryos 0.2–0.3 mm, prodissoconch stage with shells 0.3 to 0.7 mm long, largest embryo ~0.8 mm] (Meier-Brook, 1970, 1977)	demibranchs of each side and walls of visceral sac.	1977: p. 54)		
	Pisidium hibernicum [early embryos 0.1–0.2 mm, later embryos 0.3–0.8 mm]	Brooding: in brood pouches formed by gill filaments.			Meier-Brook (1970)
	Pisidium nitidum [early embryo (egg?) 0.04 mm, largest embryo 1.15 mm]	Brooding: in brood pouches formed by gill filaments.			Meier-Brook (1970)
	Pisidium conventus [early embryos smaller than 0.3 mm, later embryos larger than 0.8 mm, up to 1.0–1.25 mm]	Brooding: in brood pouches formed by gill filaments.			Meier-Brook (1970)
	Pisidium forense [early embryos 0.1–0.15 mm, juveniles at birth about 1.0 mm]	Brooding: in brood pouches formed by gill filaments.			Meier-Brook (1970)
	Pisidium obtusale [earliest embryos observed 0.3–0.6 mm, largest embryo 1.1 mm]	Brooding: in brood pouches formed by gill filaments.			Meier-Brook (1970)
	Pisidium amnicum [smallest embryos observed 0.3–0.4 mm, presumed birth size 0.5–1.0 mm] (Meier-Brook, 1970);	Brooding: in brood pouches formed by gill filaments.			Meier-Brook (1970); Araujo *et al*. (1999); Hetzel (1993) in Korniushin & Glaubrecht (2003)

	[smallest embryos 0.08 mm, maximal size of embryos (larvae) in gravid animals 2.26±0.09 mm (S.E.M.); in the last month of incubation, larval growth was highest] (Araujo *et al*., 1999: p. 124)				
	Pisidium dubium [range of length of post-dissoconch embryos in 1-year old parents: 0.192–0.744 mm (in April), 1.008–1.701 mm (in May); in 3-years old parents: 0.168–0.960 mm (in April), 1.386–2.520 mm (in May); from April through May…embryos increased in size more than two-fold] (Heard, 1965: pp. 386, 388)	Brooding: [in marsupial sacs].			Heard (1965)
	Pisidium fallax {post-dissoconch embryos 0.1–0.87 mm}, [the size of the embryos increases as the size of the parent increases] (Heard, 1965: p. 394)	Brooding: [in marsupial sacs].			Heard (1965)

	Pisidium henslowanum [largest ova 34.0–36.0 µm ø, zygotes <20.0–28.0 µm ø, embryos in broodsacs 44.0–68.0 µm ø, fetal larvae in broodsacs 96.0–124.0 µm ø, prodissoconch larvae <156.0 µm, extramarsupial larvae <224.0–360.0 µm ø, <224.0–296.0 µm in length]	Brooding: [in brood sacs].	Brood sac wall has [large vesicular cells that gradually grow rich in nutrients for the embryos] (Pettinelli & Bicchierai, 2009: p. 84)		Pettinelli & Bicchierai (2009)
	Parapisidium reticulatum [oocytes 160.0–170.0 µm, largest juvenile 0.83 mm]	Brooding: between inner demibranch lamellae, no brood pouches; [juveniles are partly surrounded by thick tissue formed in both the ascending and descending gill lamellae and consisting of enlarged cells] (Korniushin & Glaubrecht, 2006: p. 190)	[During later stages of incubation the larvae are surrounded by large cells similar to nourishing cells in other sphaeriids and probably with similar function. …The large size of the released embryos (greater than 0.8 mm) relative to the size of the eggs (up to 0.17 mm) is here considered as indicative of matrotro-phy…, i.e. of providing nourishment other than yolk only] (Korniushin & Glaubrecht, 2006: pp. 185, 193)		Korniushin & Glaubrecht (2006)
	Pisidium casertanum	Brooding: in brood			Bespalaya *et al.* (2015)

	[length of embryos of 1-2 size classes 0.05–0.04 mm, length of embryos of 3-4 size classes 0.5–>0.8 mm, length of juveniles at birth 0.8–1.6 mm]	sacs inside the inner gills.			
	Non-identified sphaeriid (as *Calyculina truncata*)	Brooding: in brood pouches in inner gills.	[Very thick glandular cells] in the inner wall of brood pouch; [undoubted evidence of secretion in the brood pouch] (Gilmore, 1917: p. 23)		Gilmore (1917)
	Sphaerium spp.	Brooding.	[There is evidence that food is passed from the maternal tissues to the embryos] (Purchon, 1977: p. 272)		Purchon (1968, 1977); Brahmachary (1989)
	Sphaerium spp.<br>*Musculium* spp.<br>*Pisidium* spp.	Brooding: in brood sacs.	Layer of large cells in the internal wall of the brood pouch; [those cells provide nourishment to the developing embryos] (Korniushin & Glaubrecht, 2006: p. 193)		Korniushin & Glaubrecht (2006)
**Family Unionidae**	*Pyganodon cataracta* [early glochidia 120.0–130.0 μm, mature glochidia shell length 380.0 μm]	Brooding: in marsupial chamber formed by primary water tubes of entire outer demibranchs.	[Large deposits of glycogen were present in the interlamellar septa … until midway through brooding. The abundant microvilli on the surface of these septa…provided extensive contact between		Schwartz & Dimock (2001[1]); Tankersley & Dimock (1992)

			the glochidia and parental tissues and could facilitate nutritional exchange] (Schwartz & Dimock, 2001: p. 233); [the narrow (anteriorposterior dimension) arrangement of the individual brood chambers…keeps the larvae in close contact with the interfilamentar septa and may facilitate the transfer of nutrients from the female to developing larvae] (Tankersley & Dimock, 1992: p. 153)		
	Utterbackia imbecillis [early glochidia 120.0–130.0 µm, mature glochidia shell length 250.0 µm]	Brooding: in marsupial chamber formed by primary water tubes of entire outer demibranchs.	[Cells of the interlamellar septa forming the transverse boundaries of the brood chambers… early in brooding were filled with large deposits of glycogen; …septa were covered with numerous branched and unbranched microvilli…that increased the surface area and appeared to provide physical contact between glochidia and parental tissues]		Schwartz & Dumock (2001[1])

			(Schwartz & Dimock, 2001: p. 231)		
	Anodonta cygnea	Brooding: in interlamellar spaces of demibranchs.	Mucus-secreting epithelium of demibranchs.	[Glochidia ingest food. This almost certainly comes from adult, probably as mucus, secreted by cells in the interlamellar connections] (Wood, 1974: p. 9)	Wood (1974[2])
	Anodonta grandis [egg ø 0.15 mm] (Richard *et al*., 1991)	Brooding: in marsupial chamber formed by medial water tubes of lateral demibranchs.	Passage of maternal calcium to glochidia (Silverman *et al*., 1987: p. 137)	[Each embryo (is) in direct contact with the septal epithelium (that) could be important in calcium transfer or other exchanges (gas, other nutrients) between the female and the embryo] (Richard *et al*., 1991: p. 1753)	Silverman *et al*. (1985[2], 1987[2]); Richard *et al*. (1991)
	Ligumia subrostrata	Brooding: in brood chamber formed by the central water channel of the posterior half of lateral demibranch.	[Ca(lcium) from a maternal source is deposited in the glochidial shells forming in the water channel of the gills] (Silverman *et al*., 1987: p. 145)		Silverman *et al*. (1985[2], 1987[2]); Richard *et al*. (1991)
	Carunculina parva texasensis	Brooding: in brood chamber formed by the central water channel of the posterior half of lateral demibranch.	[Ability to provide essential nutrients to brooding larvae] (Richard *et al*., 1991: p. 1753)		Richard *et al*. (1991)
**Family Hyriidae**	*Hyridella depressa* [gastrula mean ø	Brooding: in marsupial pouch in the middle third	Thickened interlamellar septa.		Jupiter & Byrne (1997)

	151.0 µm, glochidium mean length 243.0 µm, mean height 249.0 µm] [embryos increase in size as they developed to the glochidia stage] (Jupiter & Byrne, 1997: p. 181);	portion of inner demibranchs.			
**Family Teredinidae**	*Lyrodus pedicellatus* [430-fold increase in ash-free dry weight during the brood period] (Calloway, 1982: p. 860)	Brooding: in closed pouches in adult gills.	[“Placental” function… (of) the gill epithelium surrounding the larvae… (that) is heavily laden with intracellular glycogen] (Lane *et al*., 1952: p. 388); [cells of brood pouch wall with large quantities of glycogen; extraembryonic source of nutrition, adults… transfer nutrients to embryos] (Calloway, 1982: p. 860)	[Detached wall cells are free in the lumen of the pouch and the guts of embryos] (Calloway, 1982: p. 860)	Lane *et al*. (1952); Calloway (1982²)
**Class Gastropoda Coenogastropoda Family Thiaridae**	*Melanoides tuberculata* [egg 60.0 µm, juveniles up to 2.6–3.4 mm tall] (Berry & Kadri, 1974); [smallest-shelled embryo 0.07 mm in shell height, largest juvenile 2.33 mm in shell height] (Ben-Ami & Hodgson, 2005];	Brooding: in [subhaemocoelic brood pouch] (Glaubrecht, 2006: p. 1).	Epithelium lining of subhaemocoelic brood pouch; [brood-pouch produces secretions] (Berry & Kadri, 1974: p. 380); brood pouch [epithelium… has a nutritive function] (Stagl, 1993: p. 187); [nutritive tissue; “pseudoplacental”	[Many eggs fail to develop, …perhaps providing further nourishment for the remainder] (Berry & Kadri, 1974: p. 380); [juveniles…within the brood pouch after hatching from the egg capsule, feed…on	Berry & Kadri (1974); Stagl (1993); Glaubrecht (1999, 2006); Von Rintelen & Glaubrecht (2005); Strong & Glaubrecht (2007); Ben-Ami & Hodgson (2005: p. 322): [embryos derive little nutrition from

	[height×width of apical whorl of embryo 80.9×83.9±8.64 µm, maximum hatchling height 4.3 mm] (S.D.) (Strong & Glaubrecht, 2007)		nourishment of the young; juveniles are nourished…by matrotrophy, …by nutrients secreted from placenta-like epithelium lining the inside of the subhaemocoelic brood pouch] (Glaubrecht, 2006: pp. 3-4)	nutritive secretions from the brood pouch epithelium] (Strong & Glaubrecht, 2007: p. 101)	the mother].
	Tarebia granifera [height×width of apical whorl of embryo (~egg ø) 53.0×76.0±7.3 µm, maximum hatchling height 10.0 mm] (S.D.) (Strong & Glaubrecht, 2007)	Brooding: in [subhaemocoelic brood pouch].	As above.	As above.	Glaubrecht (1999, 2006); Von Rintelen & Glaubrecht (2005); Strong & Glaubrecht (2007)
	Thiara scabra [width of apical whorl of embryo (~egg ø) 82.0 µm, maximum hatchling height 3.0 mm] (Strong & Glaubrecht, 2007)	Brooding: in [subhaemocoelic brood pouch].	[Pseudoplacenta, matrotrophy].	Histophagy, [viviparous adelphophagy] =embryophagy (Strong & Glaubrecht, 2007: p. 101)	Muley (1977); Glaubrecht (1999, 2006); Von Rintelen & Glaubrecht (2005); Strong & Glaubrecht (2007)
	"*Thiara*" *australis* [embryos less than 0.5 mm, shelled juveniles from 0.5 to over 3.0 mm]	Brooding: in [subhaemocoelic brood pouch].			Glaubrecht *et al*. (2009): clear evidence for matrotrophy was not found; N. Maaß & M. Glaubrecht, unpublished data
	Melasma onca [embryos less than 0.5 mm, shelled juveniles from 0.5 to 2.5 mm]	Brooding: in [subhaemocoelic brood pouch].			Glaubrecht *et al*. (2009): clear evidence for matrotrophy was not found;

					N. Maaß & M. Glaubrecht, unpublished data
	Sermyla venustula [embryos less than 0.5 mm, shelled juveniles from 0.5 to over 3.0 mm]	Brooding: in [subhaemocoelic brood pouch].			Glaubrecht *et al*. (2009): clear evidence for matrotrophy was not found; N. Maaß & M. Glaubrecht, unpublished data
	Hemisinus brasiliensis [height×width of apical whorl of embryo (~egg ø) 63.7×96.4±6.1 µm, maximum hatchling height 3.0 mm] (S.D.) (Strong & Glaubrecht, 2007)	Brooding: in [subhaemocoelic brood pouch].	[Matrotrophy].	[Juveniles…within the brood pouch after hatching from the egg capsule, feed… on nutritive secretions from the brood pouch epithelium] (Strong & Glaubrecht, 2007: p. 101)	Glaubrecht (1996) in Von Rintelen & Glaubrecht (2005); Strong & Glaubrecht (2007)
	Fijidoma maculata [maximum hatchling height ~1.6 mm] (Strong & Glaubrecht, 2007); [early cleavage stages with ø of 0.07 mm, embryos with the shells up to four whorls and shell height of 2.3 mm; the volume of the embryos undergoes an enormous increase during…development] (Hubendick, 1952: pp. 181-182)	Viviparity: in uterus.	Embryonic [velum and uterus wall may possibly function as a sort of placenta] (Hubendick, 1952: p. 182)	[Richly lobated…velum takes part in the feeding mechanism of the embryo] (Hubendick, 1952: pp. 181-182)	Hubendick (1952); Strong & Glaubrecht (2007)
**Family Paludomidae**	Genus *Lavigeria*	Viviparity: in uterine	The lower lateral lamina		Kingma & Michel (2000);

	including *Lavigeria coronata* [oocyte 0.5–1.0 mm, V-stage embryo 1.0–1.75 mm]	brood pouch=pallial oviduct.	(of pallial oviduct) acts as an attachment point for drapes of fine tissue which hang between embryos as they are brooded in the pallial oviduct] (Michel 1995: p. 299); [matrotrophy].		Michel (1995); Michel *et al*. (2009); McIntyre *et al*. (2009²)
	Lavigeria grandis [oocyte 0.25–1.0 mm, V-stage embryo 0.75–2.5 mm]	Viviparity: in uterine brood pouch=pallial oviduct.	[Matrotrophy].		Kingma & Michel (2000)
	Lavigeria nov. sp. M [oocyte 0.25 mm, V-stage embryo 0.5–1.25 mm]	Viviparity: in uterine brood pouch=pallial oviduct.	[Matrotrophy].		Kingma & Michel (2000)
	Lavigeria nov. sp. J [oocyte 0.25–0.75 mm, V-stage embryo 0.75–2.0 mm]	Viviparity: in uterine brood pouch=pallial oviduct.	[Matrotrophy].		Kingma & Michel (2000)
	Lavigeria paucicostata [oocyte 0.25 mm, V-stage embryo 0.75–2.0 mm]	Viviparity: in uterine brood pouch=pallial oviduct.	[Matrotrophy].		Kingma & Michel (2000)
	Lavigeria nov. sp. B [oocyte 0.25 mm, V-stage embryo 0.75–1.5 mm]	Viviparity: in uterine brood pouch=pallial oviduct.	[Matrotrophy].		Kingma & Michel (2000)
	Lavigeria sp. A [fertilized egg about 217 µm in ø, maximum height of embryos	Viviparity: in brood pouch.			Strong & Glaubrecht (2007)

	within brood pouch about 288 μm] (Strong & Glaubrecht, 2007: p. 94)				
	Tiphobia horei [egg 520.0 μm, juveniles 1.14×1.23 mm, 1.21×1.47 mm]	Viviparity: in pallial oviduct.			Strong & Glaubrecht (2007: p. 87) [no evidence of adelphophagy]= embryophagy
**Family Planaxidae**	*Planaxis sulcatus* [oocyte 56.08±9.44 μm] (S.D.) (Ahmed & Siddiqui, 1997); [veliger width 131.0–152.0 μm] (Ohgaki, 1997)	Viviparity: in pallial oviduct.		Feeding on nurse-eggs (Thorson, 1940 in Baur, 1994)	Thorson (1940) in Baur (1994); Webber (1977); Ahmed & Siddiqui (1997); Ohgaki (1997)
**Family Janthinidae**	*Janthina janthina*	Viviparity: in ovary and three lateral brood pouches of oviduct.	[Mucus-secreting section of the oviduct] (Graham, 1954: p. 4)	[Embryos…may make excursions into the part secreting mucus to ingest the histotrophe produced there] (Graham, 1954: p. 4); [embryos ingest mucus produced by glands in oviduct] (Lalli & Gilmer, 1989: p. 18)	Graham (1954); Lalli & Gilmer (1989); Fretter & Graham (1994)
**Family Acmaeidae**	*Erginus moskalevi* [eggs 0.15 mm, embryonic shell 0.3 mm]	Brooding: in mantle [nuchal] cavity.			Golikov & Kussakin (1972)
	Erginus rubella [eggs 75.0–100.0 μm, embryonic shell slightly less than 1.00 mm]	Viviparity: in [brood chamber]=distalmost part of female duct.	[Nutrition for the developing embryos could be provided by the columnar epithelial cells]		Thorson (1935) in Lindberg (1983)

			of the brood chamber (Lindberg, 1983: p. 144)		
**Eupulmonata Family Achatinellidae**	*Tekoulina pricei* in shelled embryos [2.5× linear increase in shell size, equalling an approximately 15× increase in volume] (Solem, 1972: p. 108)	Viviparity: in uterine oviduct.	Embryos are covered by [be-stalked sac] with two stalks of the sac [appressed to the inner, columellar side of the uterine oviduct, which is thick and noticeably glandular; …gradual transfer of nutritive materials from the parent to the embryo during growth] (Solem, 1972: pp. 107-108)		Solem (1972); Tompa (1984); Baur (1994)
	Achatinella bellula [length of fertilized egg with embryo and granular nutriment in capsule 0.75–0.78 mm, length of the smallest shelled embryo with no nutriment in capsule 1.78 mm, largest shelled embryo in capsule 3.78 mm in length; 11.5× increase in volume between the largest and the smallest shelled embryos] (Solem, 1972: p. 107); [largest embryo	Viviparity: in uterine oviduct.	[Supplemental nutritive transfer] *via* egg membrane (capsule) (Solem, 1972: p. 107)		Solem (1972); Baur (1994)

	found in the female reproductive tract was 92 times the size of a fertilized egg] (Baur, 1994: p. 9)				
**Family Acavidae**	*Stylodon studeriana*	Viviparity: in uterus.	[Massive long-term calcium transfer from parental tissues to embryos through placenta-like podocyst] (Tompa, 1984: p. 124)	Placenta-like podocyst formed by embryo [complete, round sac, enveloping the body of the entire embryo] (Tompa, 1984: p. 123)	Tompa (1984[2])
	Stylodon unidentata	Viviparity: in uterus.		Placenta-like podocyst formed by embryo.	Tompa (1984[2])
**Family Veronicellidae**	*Pseudoveronicella zootoca* [parent 29 mm (in length) had eight embryos, the largest was 11–12 mm long] (Degner, 1934: p. 299 in Solem, 1972: p. 106)	Viviparity: in uterine oviduct.			Degner (1934); Solem (1972); Baur (1994)
	Pseudoveronicella pauliani [parent 21 mm (in length) had four embryos, the largest was 8 mm long] (Forcart, 1953: p. 52 in Solem, 1972: p. 106)	Viviparity: in uterine oviduct.			Forcart (1953); Solem (1972); Baur (1994)

**<u>Phylum Annelida</u>**<br>**Class Polychaeta**<br>**Family Ctenodrilidae**	*Raphidrilus nemasoma*	Viviparity: in maternal coelom.			Schroeder & Hermans (1975);<br>Schroeder (1989);<br>Wilson (1991)
**Family Nereidae**	*Hediste limnicola*	Viviparity: in maternal coelom.		[Active feeding upon coelomic corpuscles of mother] (Smith, 1950: p. 436)	Smith (1950);<br>Schroeder & Hermans (1975);<br>Schroeder (1989);<br>Wilson (1991)
**Family Spionidae**	*Streblospio benedicti* [uncleaved eggs 53.0–76.0 µm, 7-setiger incubated larva 1.9 mm] (Dean, 1965); [planktotrophic development: eggs 70.0–90.0 µm, 3-7-setiger larva at release 200.0–300.0 µm; lecithotrophic development: eggs 100.0–220.0 µm, 9-12-setiger larva at release 500.0–650.0 µm] (Levin, 1984 *a*, *b*)	Viviparity: incubation in the coelom, and, further in [coelomic brood pouches] (Levin, 1984*a*);<br>[paired dorso-lateral swellings – brood pouches] (Dean, 1965: p. 68)	[Larviparous].		Dean (1965);<br>Levin (1984*a*, *b*);<br>Schroeder (1989);<br>Wilson (1991);<br>Blake & Arnofsky (1999)
	Rhynchospio arenicola asiatica [larvae are released at the 3-setiger stage, 175.0 µm] (Levin, 1984*b*:	Viviparity: [in dorsal brood pouches].	[Larviparous].		Levin (1984*b*);<br>Blake & Arnofsky (1999)

	p. 1188)				
	Boccardia natrix	Viviparity: in maternal coelom.	[Larviparous].	[Larvae feed on coelomic content and perhaps nurse eggs] (Söderström, 1920 in Blake & Arnofsky, 1999: p. 95)	Söderström (1920) in Blake & Arnofsky (1999)
**Family Syllidae**	*Syllis vivipara* [embryos grow within the body cavity of the parent to an advanced stage] (Krohn, 1869 in Goodrich, 1900: p. 106); [as the embryos grow larger they have some difficulty in accommodating themselves within the parental body cavity] (Goodrich, 1900: p. 106)	Viviparity: in maternal coelom.		[Embryos must obtain nourishment from the coelomic fluid of the mother; either by mere diffusion through the general surface of the body, or by the mouth or anus] (Goodrich, 1900: p. 107)	Krohn (1869) in Goodrich (1900); Goodrich (1900); Franke (1999)
	Syllis nepiotica	Viviparity: in maternal coelom.			Franke (1999)
	Syllis incisa	Viviparity: in maternal coelom.			Augener (1929); Franke (1999)
	Syllis botosaneaunui	Viviparity: in maternal coelom.			Aguado & San Martín (2006)
	Syllis parturiens [most advanced embryos…(with) 6 setigers – seen through body wall of parent] (Ben-Eliahu, 1977: p. 23)	Viviparity: in maternal coelom.			Ben-Eliahu (1977); Franke (1999)
	Parexogone	Viviparity: in maternal			San Martín Peral (1984);

	parahomoseta mediterranea	coelom.			Franke (1999)
	Exogone hebes [fully differentiated juveniles (7 segments long) within modified (maternal) segments] (Pocklington & Hutcheson, 1983: p. 239)	Viviparity: in maternal coelom.			Pocklington & Hutcheson (1983); Franke (1999)
	Dentatisyllis mangalis coelomic [young… with up to 13 setigers] (Russell, 1995: p. 571)	Viviparity: in maternal coelom.			Russell (1995); Franke (1999)
	Dentatisyllis mortoni	Viviparity: in maternal coelom.		[Juveniles appear to take up coelomic fluid (of the mother animal) with their hind gut; epidermal uptake of dissolved organic matter may also be very likely] (Ding *et al*., 1998: p. 41)	Ding *et al*. (1998); Franke (1999)
	Sphaerosyllis lateropapillata uteae	Viviparity: in maternal coelom.			Hartmann-Schröder (1993)
**Family Cirratulidae**	*Tharyx vivipara* [mature eggs 100.0–120.0 µm, advanced embryos of 6 segments up to 450.0 µm]	Viviparity: in maternal coelom.			Christie (1984)
**Family Ampharetid[illegible]**	*Alkmaria romijni* [inside the worms big embryos … are found] (Wesenberg-Lund, 1934:	Viviparity: in maternal coelom.			Wesenberg-Lund (1934)

	p. 219)				
**Family Geobangiida**	*Geobangia brandti* [incubated larvae 0.60–0.95 mm in length, 0.20 mm wide]	Viviparity: [in larval ducts].	[Larviparous].		Jones (1974)
	Geobangia abbotti {fixed oocyte 39.0×25.0 µm}, [incubated larvae 0.60–0.95 mm in length, 0.20 mm wide; walls of (larval) ducts are capable of considerable extension for… developing embryos and larvae] (Jones, 1974: p. 27) (in photos embryos considerably larger than eggs)	Viviparity: [in larval ducts].	[Larviparous].		Jones (1974)
**Class Clitellata**<br>**Family Glossiphoniidae**	*Glossiphonia complanata*<br>[egg 0.80×0.75 mm] (Whitman, 1878);<br>[egg ~600.0 µm] (Kutschera & Wirtz, 2001);<br>[embryos at hatching 700.0 µm embryos at phase III up to 1.5 mm, embryos at phase IV up to 2.0 mm] (de Eguileor *et al*., 1994)	Brooding: on the ventral side of the body wall.	Epithelium of ventral body side [sort of “placenta”] (Kutschera & Wirtz, 2001: p. 127); [transfer of nutrients across the parental and young epithelia; weight loss in a parent]<br>(de Eguileor *et al*., 1994: p. 400)	Epithelium of posterior sucker.	Whitman (1878);<br>de Eguileor *et al*. (1993[1]);<br>de Eguileor *et al*. (1994[1,2]);<br>Kutschera & Wirtz (2001)

	Helobdella stagnalis<br>[eggs 0.29–0.30 mm] (Tillman & Barnes, 1973);<br>[eggs 0.55×0.50 mm] (Whitman, 1878);<br>[egg ~500.0 µm] (Kutschera & Wirtz, 2001);<br>[juveniles become independent when diameter of the posterior sucker reaches about 0.40 mm] (Learner & Potter, 1974: p. 204)	Brooding: on the ventral side of the body wall.	Epithelium of ventral body side;<br>[maternal transfer; trophic relationships… between the maternal body and the young] (Cornec, 1978: p. 113)	Epithelium of posterior sucker.	Whitman (1878);<br>Tillman & Barnes (1973);<br>Learner & Potter (1974);<br>Cornec (1978[1]);<br>Needham (1990);<br>Kutschera & Wirtz (2001)
	Marsupiobdella africana<br>[mature eggs 260.0 µm, newly discharged young average 0.77 mm]	Brooding: in the ventral [brood pouch].			Lande & Tinsley (1976)
<u>**Phylum Bryozoa**</u><br>**Class Phylactolaemata**<br><br>**Family Cristatellidae**	All 87 species described (6 families);<br>examples:<br>*Cristatella mucedo*	Brooding: in a brood sac, invagination of the body wall.	Spot-like placenta.		Reed (1991)<br><br>Jullien (1890);<br>Davenport (1891)
**Family Fredericellidae**	*Fredericella sultana*	Brooding: in a brood sac, invagination of the body wall.	Spot-like placenta.		Braem (1908)

**Family Pectinatellidae**	*Pectinatella magnifica*	Brooding: in a brood sac, invagination of the body wall.	Spot-like placenta.		Braem (1908)
**Family Plumatellidae**	*Plumatella* spp.	Brooding: in a brood sac, invagination of the body wall.	Ring-like placenta.	In *Plumatella fungosa* embryonic cells of the placental contact can 'digest' the cells of the brood chamber (Braem, 1897)	Kraepelin (1886, 1892); Korotneff (1887, 1889); Braem (1890, 1897); Brien (1953)
	Hyalinella punctata	Brooding: in a brood sac, invagination of the body wall.	Ring-like placenta.		Mukai (1982)
**Family Lophopodidae**	*Lophopus crystallinus*	Brooding: in a brood sac, invagination of the body wall.			Marcus (1934)
**Class Stenolaemata Order Cyclostomata**<br><br>**Family Crisiidae**	All 626 species described (23 families); examples:<br>*Crisia ramosa*	 <br><br>Viviparity: in 'secondary' follicle, later in gonozooidal coelom.			Reed (1991)<br><br><br>Harmer (1890, 1893)
	Crisia denticulata	Viviparity: in 'secondary' follicle, later in gonozooidal coelom.			Calvet (1900)
	Cirisia eburnea	Viviparity: in 'secondary' follicle, later in gonozooidal coelom.	[Nutritive tissue, nutritive cylinder].		Borg (1926)
	Crisia occidentalis	Viviparity: in 'secondary' follicle, later in gonozooidal			Robertson (1903)

		coelom.			
	Filicrisia franciscana	Viviparity: in ‘secondary’ follicle, later in gonozooidal coelom.			Robertson (1903)
	Filicrisia sp.	Viviparity: in ‘secondary’ follicle, later in gonozooidal coelom.			Robertson (1903)
	Bicrisia robertsonae	Viviparity: in ‘secondary’ follicle, later in gonozooidal coelom.			Robertson (1903)
	Bicrisia edwardsiana	Viviparity: in ‘secondary’ follicle, later in gonozooidal coelom.	[Nutritive tissue, nutritive cylinder].		Borg (1926)
	Crisidia cornuta	Viviparity: in ‘secondary’ follicle, later in gonozooidal coelom.	[Nutritive tissue, nutritive cylinder].		Borg (1926)
	Crisiella producta	Viviparity: in ‘secondary’ follicle, later in gonozooidal coelom.	[Nutritive tissue, nutritive cylinder].		Borg (1926)
**Family Tubuliporidae**	*Tubulipora liliacea*	Viviparity: in ‘secondary’ follicle, later in gonozooidal coelom.	[Nutritive tissue, nutritive cylinder].		Harmer (1896a); Borg (1926)
	Tubulipora plumosa	Viviparity: in ‘secondary’ follicle, later in gonozooidal coelom.	[Nutritive tissue].		Harmer (1896*a*, 1898)
	Tubulipora phalangea	Viviparity: in ‘secondary’ follicle, later in gonozooidal coelom.	[Nutritive tissue, nutritive cylinder].		Harmer (1896*a*); Borg (1926)
	Idmidronea radians var. *erecta*	Viviparity: in ‘secondary’ follicle, later in gonozooidal coelom.			Waters (1914)
**Family**					

**Plagioeciidae**	*Plagioecia patina*	Viviparity: in 'secondary' follicle, later in gonozooidal coelom.	[Nutritive tissue, nutritive cylinder].		Borg (1926)
	Entalophoroecia wasinensis	Viviparity: in 'secondary' follicle, later in gonozooidal coelom.			Waters (1914)
**Family Horneridae**	*Hornera frondiculata*	Viviparity: in 'secondary' follicle, later in gonozooidal coelom.			Ostroumoff (1887)
	Hornera antarctica	Viviparity: in 'secondary' follicle, later in gonozooidal coelom.	[Nutritive tissue, nutritive mass].		Waters (1904*a*); Borg (1926)
	Hornera lichenoides	Viviparity: in 'secondary' follicle, later in gonozooidal coelom.	[Nutritive tissue, nutritive mass].		Waters (1904*b*); Borg (1926)
	Hornera violacea	Viviparity: in 'secondary' follicle, later in gonozooidal coelom.	[Nutritive tissue, nutritive mass].		Borg (1926)
**Family Lichenoporidae**	*Patinella verrucaria*	Viviparity: in 'secondary' follicle, later in gonozooidal coelom.	[Nutritive tissue].		Harmer (1894, 1895, 1896*b*)
	Disporella fimbriata	Viviparity: in 'secondary' follicle, later in gonozooidal coelom.	[Nutritive tissue].		Borg (1926)
	Disporella hispida	Viviparity: in 'secondary' follicle, later in gonozooidal coelom.	[Nutritive tissue].		Borg (1926)
**Class Gymnolaemata Order Ctenostomata Family**					

**Labiostomellidae**	*Labiostomella gisleni*	Brooding: in brood sac, invagination of the body wall.	Embryophore.		Ostrovsky (2008); Ostrovsky *et al*. (2008); Silén (1944); Ström (1977)
**Family Walkeriidae**	*Walkeria uwa*	Brooding: in introvert.			Joliet (1877); Ostrovsky *et al*. (2008)
**Family Nolellidae**	*Nolella stipata*	Brooding: in brood sac, invagination of the body wall.			Hincks (1880); Ostrovsky *et al*. (2008)
	Nolella dilatata	Brooding: in brood sac, invagination of the body wall.			Prouho (1892); Ostrovsky *et al*. (2008)
	Nolella papuensis	Brooding: in brood sac, invagination of the body wall.	Embryophore: [the wall of the sac… (is) rather thick] (Silén, 1944: p. 26)		Harmer (1915); Silén (1944); Ström (1977); Ostrovsky *et al*. (2008)
**Family Flustrellidridae**	*Flustrellidra hispida*	Brooding: in introvert.			Pace (1906); Ostrovsky *et al*. (2008)
**Family Mimosellidae**	*Bantariella cookae*	Brooding: in introvert.			Banta (1968); Ström (1977); Reed (1991); Ostrovsky *et al*. (2008)
**Family Vesiculariidae**	*Zoobotryon verticillatum*	Brooding: in introvert.	Embryophore: exocytosis.	Endocytosis.	Waters (1914); Ostrovsky *et al*. (2008); Ostrovsky & Schwaha (2011[1])
**Family Sundanellidae**	*Sundanella sibogae*	Brooding: in brood sac, invagination of the body wall.	Embryophore.		Braem (1939); Silén (1944); Ström (1977);

					Reed (1991); Ostrovsky *et al*. (2008)
**Order Cheilostomata Family Calloporidae**	*Crassimarginatella falcata*	Brooding: in ovicell.			Cook (1985)
**Family Flustridae**	*Retiflustra schoenaui*	Brooding: in ovicell.			Harmer (1902, 1926); Ostrovsky *et al*. (2008); Ostrovsky (2008)
	Gregarinidra serrata [zygote 100.0×75.0 µm, early embryo 102.0×75.0 µm] (Ostrovsky, 2009, 2013*a*, *b*)	Brooding: in ovicell.	Embryophore.		Ostrovsky (2009); Ostrovsky *et al*. (2009); Ostrovsky (2013*a*, *b*)
	Isosecuriflustra angusta [oocyte 180.0×170.0 µm, late embryo 225.0×155.0 µm, embryonic increase 1.27-fold] (Ostrovsky, 2009, 2013*a*, *b*)	Brooding: in ovicell.	Embryophore.		Ostrovsky (2009); Ostrovsky *et al*. (2009); Ostrovsky (2013*a*, *b*)
	Klugeflustra antarctica [early/mid-stage embryos 260.0×200.0 µm, 310.0×220.0 µm, embryonic increase 1.5-fold] (Ostrovsky, 2009, 2013*a*, *b*)	Brooding: in ovicell.	Embryophore.		Ostrovsky (2009); Ostrovsky *et al*. (2009); Ostrovsky (2013*a*, *b*)
**Family Bugulidae**	77 described species previously assigned to the genus *Bugula*, including:				

	Bugula neritina [oocyte 36.0 µm, larvae 200.0–300.0×300.0–400.0 µm, embryonic increase 500-fold] (Woollacott & Zimmer, 1975); [submature oocyte 32.0×30.0 µm, late embryo 230.0×190.0 µm, embryonic increase 310-fold] (Ostrovsky, 2009, 2013*a*, *b*)	Brooding: in ovicell.	[Placenta-like system, exocytosis]; [embryophore cells enlarged drastically] (Ostrovsky, 2013*b*: p. 1373)	[Endocytosis].	Woollacott & Zimmer (1972, 1975[1]); Ryland (1976), Dyrynda & King (1983); Reed (1991); Ostrovsky (2008, 2009, 2013*a*, *b*); Ostrovsky *et al*. (2008, 2009)
	Bugulina flabellata [oocyte 80.0 µm, late embryo 130.0 µm] (Corrêa, 1948); [oocyte 77.0 µm ($=2.4\times10^5$ µm³), late embryo 150.0 µm ($=1.7\times10^6$ µm³), embryonic increase 7.1-fold] (Dyrynda & King, 1983); [oocyte 96.0×55.0 µm, late embryo 160.0×120.0 µm, embryonic increase 6.3-fold] (Ostrovsky, 2009, 2013*a*, *b*)	Brooding: in ovicell.	As above.		Hincks (1861); Corrêa (1948); Ryland (1976); Dyrynda & Ryland (1982); Dyrynda & King (1983); Reed (1991); Ostrovsky *et al*. (2009); Ostrovsky (2009, 2013*a*, *b*)
	Bugulina turbinata [egg 5.8×10^{4} µm³, embryo 1.9×10^6 µm³, embryonic increase	Brooding: in ovicell.			Hincks (1861); Dyrynda & King (1983); Reed (1991)

	32.7-fold]<br>(Dyrynda & King, 1983)				
	Bugulina calathus<br>[egg 6.9×10^{4} μm^3,<br>embryo 1.2×10^{6} μm^3,<br>embryonic increase<br>17.4-fold]<br>(Dyrynda & King, 1983)	Brooding: in ovicell.	Embryophore.		Vigelius (1886);<br>Dyrynda & King (1983);<br>Reed (1991)
	Bugulina fulva<br>[egg 7.8×10^{4} μm^3,<br>embryo 1.5×10^{6} μm^3,<br>embryonic increase<br>19.2-fold]<br>(Dyrynda & King, 1983)	Brooding: in ovicell.			Dyrynda & King (1983);<br>Reed (1991)
	Bugulina stolonifera<br>[egg 1.4×10^{5} μm^3,<br>embryo 1.1×10^{6} μm^3,<br>embryonic increase<br>7.8-fold]<br>(Dyrynda & King, 1983)	Brooding: in ovicell.	Embryophore.		Marcus (1938);<br>Ryland (1976);<br>Dyrynda & King (1983);<br>Reed (1991);<br>T. Schwaha &<br>A. N. Ostrovsky,<br>unpublished data
	Bugulina simplex	Brooding: in ovicell.	Embryophore.		Calvet (1900);<br>Ryland (1976);
	Crisularia plumosa	Brooding: in ovicell.			Dyrynda & King (1983);<br>Reed (1991)
	Bicellariella ciliata<br>[oocyte 60.0–63.0 µm,<br>larva 132.0 µm,<br>embryonic increase<br>10-fold]<br>(Moosbrugger *et al*.,<br>2012; Ostrovsky, 2013*a*)	Brooding: in ovicell.	[Embryophore: placental analogue].	[Endocytosis].	Nitsche (1869);<br>Hincks (1861, 1873);<br>Ryland (1976);<br>Dyrynda & King (1983);<br>Reed (1991);<br>Ostrovsky *et al*. (2008);<br>Moosbrugger *et al*. (2012[1])<br>Ostrovsky (2013*a*)

**Family Beaniidae**	*Beania magellanica*	Brooding: in internal brood sac.	Embryophore.		Waters (1912, 1913); Ostrovsky (2008);
	Beania bilaminata [oocyte 55.2×50.4 µm, late embryo 490.0×330.0 µm, embryonic increase 468.2-fold] (Ostrovsky, 2009, 2013*a*, *b*)	Brooding: in internal brood sac.	Embryophore.		Ostrovsky *et al*. (2009); Ostrovsky (2009, 2013*a*, *b*)
**Family Candidae**	*Scrupocellaria ferox* [egg 1.6×10^6 µm³, embryo 8.2×10^6 µm³] {embryonic increase 5.1-fold} (Santagata & Banta, 1996)	Brooding: in ovicell.	Embryophore.		Santagata & Banta (1996); Ostrovsky *et al*. (2008)
	Scrupocellaria scruposa [early embryos 120.0×105.0 µm, 145.0×130.0 µm, embryonic increase 1.8-fold] (Ostrovsky, 2009, 2013*a*, *b*)	Brooding: in ovicell.			Ostrovsky *et al*. (2009); Ostrovsky (2009, 2013*a*, *b*)
**Family Microporidae**	*Micropora notialis* [oocyte 105.0×90.0 µm, early embryo 210.0×135.0 µm, embryonic increase 1.5-fold] (Ostrovsky, 2009, 2013*a*, *b*)	Brooding: in ovicell.	Enlarged epithelial cells of ooecial vesicle.		Ostrovsky *et al*. (2009); Ostrovsky (2009, 2013*a*, *b*)
	Mollia multijuncta [oocyte 33.6×28.8 µm, larva 175.0×60.0 µm, embryonic increase	Brooding: in ovicell.	Embryophore.		Ostrovsky (2013*a*, *b*)

	53.41-fold] (Ostrovsky, 2013*a*, *b*)				
**Family Cellariidae**	*Cellaria fistulosa* [oocyte 90.0×57.5 µm, late embryo 127.0×125.0 µm, embryonic increase 4.9-fold] (Ostrovsky, 2009, 2013*a*, *b*)	Brooding: in ovicell.	Embryophore.		Calvet (1900); Ostrovsky *et al*. (2009); Ostrovsky (2009, 2013*a*, *b*)
	Cellaria tenuirostris [oocyte 85.0×58.0 µm, late embryo 115.0×100.0 µm, embryonic increase 3.39-fold] (Ostrovsky, 2009, 2013*a*, *b*)	Brooding: in ovicell.	Embryophore.		Ostrovsky *et al*. (2009); Ostrovsky (2009, 2013*a*, *b*)
**Family Poricellariidae**	*Poricellaria ratoniensis*	Brooding: in ovicell.			Waters (1913); Ostrovsky (2008)
**Family Epistomiidae**	Presumably all 5 known species of this family, including *Epistomia bursaria* {oocyte 13.5×10.1 µm} (Dyrynda & King, 1982); {larva 0.31×0.29 mm} (Dyrynda, 1981); [very substantial increase in volume… during embryogenesis (almost 1000-fold)]	Viviparity: first in ovary, later in gonozooidal coelom.			Dyrynda (1981); Dyrynda & King (1982); Reed (1991); Ostrovsky *et al*. (2008); Ostrovsky (2008)

	(Dyrynda, 1981: p. 76; Dyrynda & King, 1982)				
	Synnotum circinatum [blastula 30.0 µm, larva 180.0×90.0×95.0 µm, embryonic increase 50-60-fold] (Marcus, 1941)	Viviparity: first in ovary, later in gonozooidal coelom.			Marcus (1941); Ostrovsky (2008)
**Family Cribrilinidae**	*Figularia figularis* [oocyte 240.0×180.0 µm, late embryo 260.0×220.0 µm, embryonic increase 1.49-fold] (Ostrovsky, 2009, 2013*a*, *b*)	Brooding: in ovicell.	Embryophore.		Ostrovsky *et al*. (2009); Ostrovsky (2009, 2013*a*, *b*)
**Family Catenicellidae**	*Catenicella elegans*	Brooding: in ovicell.	Embryophore.		Waters (1913); Marcus (1938); Ryland (1976); Ostrovsky *et al*. (2008); Ostrovsky (2008);
	Cribricellina cribraria [oocyte 370.0×300.0 µm, late embryo 560.0×440.0 µm, embryonic increase 3.3-fold] (Ostrovsky, 2009, 2013*a*, *b*)	Brooding: in ovicell.	Embryophore.		Ostrovsky *et al*. (2009); Ostrovsky (2009, 2013*a*, *b*)
	Costaticella solida [early embryo 190.0×165.0 µm, late embryo 365.0×240.0 µm, embryonic increase	Brooding: in ovicell.	Embryophore.		Ostrovsky *et al*. (2009); Ostrovsky (2009, 2013*a*, *b*)

	4.9-fold] (Ostrovsky, 2009, 2013*a*, *b*)				
	Costaticella bicuspis [early embryos 320.0×210.0 µm, 420.0×260.0 µm, embryonic increase 2.1-fold] (Ostrovsky, 2009, 2013*a*, *b*)	Brooding: in ovicell.	Embryophore.		Ostrovsky *et al*. (2009); Ostrovsky (2009, 2013*a*, *b*)
	Pterocella scutella [oocyte 63.0×62.5 µm, early embryo 235.0×170.0 µm, embryonic increase 33-fold] (Ostrovsky, 2009, 2013*a*, *b*)	Brooding: in ovicell.	[Embryophore epithelial cells enlarged drastically] (Ostrovsky, 2013*b*: p. 1373)		Ostrovsky *et al*. (2009); Ostrovsky (2009, 2013*a*, *b*)
**Family Hippothoidae**	*Celleporella hyalina* [egg 80.0 µm, larva 200.0 µm, embryonic increase 15.6-fold] (Hughes, 1987); [oocyte 80.0×70.0 µm, late embryo 170.0×140.0 µm, embryonic increase 8.8-fold] (Ostrovsky, 2009, 2013*a*, *b*)	Brooding: in ovicell.	Embryophore: [nutrient-storage tissue].	[Pinocytosis].	Marcus (1938); Ryland (1976); Hughes (1987[1]); Reed (1991); Ostrovsky (1998, 2009, 2013*a*, *b*); Ostrovsky *et al*. (2008, 2009)
	Celleporella carolinensis [young embryo 55.0 µm, larva near liberation 120.0-140.0 µm]	Brooding: in ovicell.			Ryland (1979)
**Family Adeonidae**	*Adeonella lichenoides*	Brooding: in internal brood sac=[ovicellular sac].	[Thick-walled sac] (Waters, 1912: p. 495)		Waters (1912, 1913); Ostrovsky (2008)

	Adeonella polymorpha	Brooding: in internal brood sac.	As above.		Waters (1912); Ostrovsky (2008)
	Adeonella polystomella	Brooding: in internal brood sac.	As above.		Waters (1912, 1913); Ostrovsky (2008)
	Adeonellopsis sp.	Brooding: in internal brood sac.			Waters (1912); Ostrovsky (2008)
	Laminopora contorta	Brooding: in internal brood sac.	As above.		Waters (1912); Ostrovsky (2008)
**Family Watersiporidae**	Apparently all 13 known species of the genus *Watersipora*, including: *Watersipora cucullata*	Brooding: in internal brood sac.	Embryophore.		Mawatari (1952)
	Watersipora arcuata	Brooding: in internal brood sac.			R. L. Zimmer, personal communication in Reed (1991)
	Watersipora subtorquata [early embryo 126.2×100.0 µm, late embryo 163.0×147.5 µm, embryonic increase 3-fold] (Ostrovsky, 2009, 2013*a*, *b*)	Brooding: in internal brood sac.	Embryophore.		Ostrovsky *et al*. (2009); Ostrovsky (2009, 2013*a*, *b*)
**Family Hippopodinidae**	*Hippopodina feegensis*	Brooding: in ovicell.			Marcus (1938); Ryland (1976)
**Family Myriaporidae**	*Myriapora truncata*	Brooding: in ovicell.	Embryophore.		Ostrovsky *et al*. (2009); Ostrovsky (2009, 2013*a*,*b*)
**Family Lanceoporidae**	‘*Calyptotheca*’ *variolosa*	Brooding: in ovicell.	Embryophore.		Ostrovsky *et al*. (2009);

	[oocyte 250.0×190.0 µm, late embryo 460.0×320.0 µm, embryonic increase 5.57-fold] (Ostrovsky, 2009, 2013*a*, *b*)				Ostrovsky (2009, 2013*a*, *b*)
**Family Urceoliporidae**	*Urceolipora nana* [oocyte 100.0×50.0 µm, late embryo 180.0×145.0 µm, embryonic increase 10.17-fold] (Ostrovsky, 2009, 2013*a*, *b*)	Brooding: in ovicell.	[Embryophore epithelial cells enlarged drastically] (Ostrovsky, 2013*b*: p. 1373)		Ostrovsky *et al*. (2009); Ostrovsky (2009, 2013*a*, *b*)
	Reciprocus regalis [oocyte 54.0×45.0 µm, late embryo 370.0×260.0 µm, embryonic increase 257.7-fold] (Ostrovsky, 2009, 2013*a*, *b*)	Brooding: in internal brood sac.	As above.		Ostrovsky *et al*. (2009); Ostrovsky (2009, 2013*a*, *b*)
<u>**Phylum Kamptozoa**</u> **Family Loxosomatidae**	*Loxosomella leptoclini*	Brooding: in brood pouch.	Glandular epithelium of the brood pouch; [placenta-like structure at the bottom of the brood pouch] (Nielsen, 2005: p. 405)		Harmer (1885); Nielsen (2005)
	Loxosomella vivipara [egg 10.0 µm, fully grown larva 200.0 µm] (Nielsen, 1990)	Brooding: in brood pouch.	As above.		Nielsen (1966, 1971, 1990, 2005)
	Loxosoma davenporti	Brooding: in brood pouch.	Embryophore – epithelium of the brood	[Cells, laden with nutrients, loosen from the	Nickerson (1901); Nielsen (1990)

			pouch [mammary organ] formed by [thickened epithelium of the atrium floor, which cells contain an elaborated food material…for the purpose of supplying nutriment to the developing embryos] (Nickerson, 1901: pp. 368-369)	embryophore and become ingested by the larvae] (Nielsen, 1990: p. 203); [contents of…cells (of mammary epithelium) have been absorbed by the embryo] (Nickerson, 1901: p. 369)	
**Family Pedicellinidae**	*Pedicellina cernua*	Brooding: in brood pouch.	Embryophore – epithelium of the brood pouch.	Feeding on secretions and 'nutritive cells' derived from embryophore walls.	Becker (1937); Cori (1936); Mariscal (1975); Nielsen (1990)
	Loxosomatoides sirindhornae	Brooding: in brood pouch.	As above.	Feeding on detached cells of embryophore walls.	Schwaha *et al*. (2010); T. Schwaha, personal observations 2012
**Phylum Cycliophora** **Family Symbiidae**	*Symbion pandora* [oocyte 22.0–34.0 µm], {chordoid larva 189.0×72.0 µm} (Funch & Kristensen, 1997)	Asexual reproduction: development of Prometheus and Pandora larvae as well as female in a feeding stage and dwarf males inside Prometheus larva: matrotrophic brooding in a cuticle-bound [fluid-filled cavity of the brood chamber] (Obst & Funch, 2003: p. 265); Sexual reproduction:	Matrotrophic brooding inside the brood chamber of a feeding stage: [each individual (feeding stage) contained a large larva (here Pandora) in a kind of pseudoplacental development… The larva attaches to the pseudoplacental wall at the posterior end by a double layer of	[Chordoid larva develops inside the female nourished by dying female cells] (Funch & Kristensen, 1997: p. 427); [dwarf males…obtain most of the nutrition for their development from the cells of the Prometheus larva] (Obst & Funch, 2003: p. 275); [the embryo developing	Funch & Kristensen (1997); Obst & Funch (2003); Neves *et al*. (2012)

		viviparity: chordoid larvae develops inside female, being surrounded by its tissues.	supporting cells] (Funch & Kristensen, 1997: p. 415); [the posterior end of the embryo (female) attaches to a double-walled pseudoplacenta] (Funch & Kristensen, 1997: p. 416); [a mantle of nourishing cells…forms pseudoplacenta, in intimate contact with the poster end of the Pandora larva] (Funch & Kristensen, 1997: p. 418); during matrotrophic brooding inside the brood chamber of the Prometheus larva and viviparous incubation in a female all the internal structures of the parent degenerate to nourish either chordoid larva or dwarf males.	inside the encysted female is nourished by its degenerative body] (Neves *et al*., 2012: p. 852)	
	Symbion americanus [egg or zygote average ø 25.0–34.0 µm, chordoid larva 184.0×76.0 µm] (Obst *et al*., 2006)	Brooding and viviparity as above.	As above.	As above.	Obst *et al*. (2006)

**<u>Phylum Nemertea</u>**<br>**Class Enopla**<br>**Family Prosorhochmidae**	*Notogaeanemertes folzae*<br>[fully mature oocytes 130.0 µm, pre-release 'juveniles' 6.0–8.0 mm]	Viviparity: in closed female gonoduct=[embryo sac].	Cells of female gonoduct wall; [true vivparity].		Crandall *et al*. (1998)
	Arhochmus korotneffi<br>[brooded young show through the body wall of the mature individual as large opaque, lighter-coloured areas between the intestinal diverticula]<br>(Maslakova & Norenburg, 2008*b*: p. 1251)	Viviparity: in ovary.			Maslakova & Norenburg (2008*b*)
	Prosorhochmus chafarinensis<br>[mature oocyte 80.0-90.0 µm]<br>(Frutos *et al*., 1998);<br>[several embryos may start cleaving, but only one per ovary develops into a juvenile]<br>(Maslakova & Norenburg, 2008*b*: p. 1229)	Viviparity: in ovary.		Embryophagy?	Frutos *et al*. (1998);<br>Maslakova & Norenburg (2008*b*)
	Prosorhochmus claparedii	Viviparity: in ovary.			Bierne (1983);<br>Crandall *et al*. (1998);

					Maslakova & Norenburg (2008*b*)
	Prosorhochmus adriaticus	Viviparity: in ovary.			Senz & Tröstl (1999) Maslakova & Norenburg (2008*b*)
	Prosorhochmus americanus	Viviparity: in ovary.			Gibson *et al*. (1986); Maslakova & Norenburg (2008*b*)
	Prosorhochmus albidus	[Occasionally viviparous]: development in ovary (Coe, 1940: p. 294)			Coe (1940); Maslakova & Norenburg (2008*b*)
	Prosadenoporus agricola [mature eggs 0.35-0.45 mm in ø, length of embryos before birth from 1.5 to 2.00 mm, ø from 0.2 to 0.25 mm] (Coe, 1904)	Viviparity: in ovary.			Coe (1904); Maslakova & Norenburg (2008*a*)
**Family Sacconemertidae**	*Cyanophthalma obscura* [mature ova 140.0 µm, length at birth up to 4.0 mm]	Viviparity: in ovary.	[Juveniles probably obtain significant nourishment from the parent] (Norenburg, 1986: p. 291)		Norenburg (1986)
**Family Tetrastemmatidae**	*Koinoporus mapochi*	Viviparity in ovary and (?matrotrophic) brooding in intestine.			Sánchez & Moretto (1988)
**Family Zygonemertidae**	*Pheroneonemertes dianae* [mature ova 150.0 µm in ø]	Viviparity: in ovary.	[Ovum is attached to its ovarian wall by a peduncle or 'placental stalk'; whether or not developing		Gibson (1990)

			embryos obtain nourishment *via* this connection is not known]; wall of the ovary with embryo is [vacuolated in appearance] (Gibson, 1990: p. 185)		
	Zygonemertes maslowskyi [newborn juveniles 1.4–1.7 mm]	Viviparity: in ovary.			Müller & Scripcariu (1967)
**Family Emplectonematidae**	*Poikilonemertes vivipara* [embryos are quite large] (Stiasny-Wijnhoff, 1942: p. 183)	Viviparity: in ovary.			Stiasny-Wijnhoff (1942)
	Dichonemertes coensis	Viviparity: in ovary.			Friedrich (1970)
**Class Anopla** **Family Lineidae**	*Lineus viviparus*	Viviparity.			Isler (1900); Coe (1943)
**Phylum Dicyemida**	All ~107 species known (3 families); examples:				Malakhov (1990); Furuya *et al*. (2003)
**Family Dicyemidae**	*Dicyema dolichocephalum* [agamete mean ø 4.5 µm, vermiform embryo length 50.0 µm, mature egg mean ø 12.0 µm, infusoriform embryo length 28.0 µm]	Viviparity: inside nematogen and rhombogen stages.			Furuya *et al*. (2003)
	Dicyema monodi [agamete mean ø 5.1 µm, vermiform embryo				Furuya *et al*. (2003)

	length 40.0 µm, mature egg mean ø 9.8 µm, infusoriform embryo length 31.0 µm]				
	Dicyema gyrinum [agamete mean ø 8.8 µm, vermiform embryo length 80.0 µm, mature egg mean ø 17.6 µm, infusoriform embryo length 29.9 µm]	Viviparity: inside nematogen and rhombogen stages.			Furuya *et al*. (2003)
	Dicyema apalachiensis [agamete mean ø 5.5 µm, vermiform embryo 30.0 µm×10.0 µm] (Furuya *et al*., 2001); [agamete mean ø 5.4 µm] (Furuya *et al*., 2003)	Viviparity: inside nematogen and rhombogen stages.			Furuya *et al*. (2001, 2003)
	Dicyema acuticephalum [vermiform embryo length is about 55 µm, body width is about 11.0 µm] (Furuya *et al*., 1994); [agamete mean ø 6.4 µm, vermiform embryo length 50.0 µm, mature egg mean ø 12.5 µm, infusoriform embryo length 29.8 µm] (Furuya *et al*., 2003)	Viviparity: inside nematogen and rhombogen stages.			Furuya *et al*. (1994, 2003)
	Dicyema japonicum [fertilized egg 12.0 µm,	Viviparity: inside nematogen and rhombogen			Furuya *et al*. (1992, 1994, 1996, 2003)

	infusoriform embryo 24.0×19.0 µm] (Furuya *et al*., 1992, 1996); [vermiform embryo body length is about 65 µm, body width is about 12 µm] (Furuya *et al*., 1994); [agamete mean ø 5.4 µm, vermiform embryo length 70.0 µm, mature egg mean ø 12.3 µm, infusoriform embryo length 23.7 µm] (Furuya *et al*., 2003)	stages.			
	Pseudiciema nakaoi [agamete 6.5 µm, vermiform embryo 70.0×16.0 µm] (Furuya *et al*., 2001); [agamete mean ø 6.5 µm, vermiform embryo length 90.0 µm, mature egg mean ø 12.2 µm, infusoriform embryo length 29.6 µm] (Furuya *et al*., 2003)	Viviparity: inside nematogen and rhombogen stages.			Furuya *et al*. (2001, 2003)
**Family Conocyemidae**	*Conocyema polymorpha* [agamete 7.0 µm, vermiform embryo length 25.0×10.0 µm]	Viviparity: inside nematogen and rhombogen stages.			Furuya *et al*. (2001, 2003)

	(Furuya *et al*., 2001);<br>[agamete mean ø 6.6 µm, vermiform embryo length 31.0 µm, mature egg mean ø 11.0 µm, infusoriform embryo length 25.3 µm]<br>(Furuya *et al*., 2003)				
	Microcyema vespa<br>[agamete 6.0 µm, vermiform embryo length 50.0 µm, width 20.0 µm]<br>(Furuya *et al*., 2001);<br>[agamete mean ø 5.8 µm, vermiform embryo length 25.0 µm, mature egg mean ø 11.3 µm, infusoriform embryo length 25.2 µm]<br>(Furuya *et al*., 2003)	Viviparity: inside nematogen and rhombogen stages.			Furuya *et al*. (2001, 2003)
**Phylum Orthonectida**<br><br>**Family Rhopaluridae**	All 24 species known (2 families);<br>examples:<br><br>*Intoshia variabili*<br>*Intoshia linei*<br>[in plasmodium: germinal cell ø 7.0–9.0 µm, female length 150.0–160.0 µm, width 31.0–33.0 µm]	Viviparity: inside plasmodium.			Malakhov (1990); Slyusarev & Miller (1998)<br><br>G. S. Slyusarev, personal communication 2013

**<u>Phylum Arthropoda</u>**<br>**Subphylum Chelicerata**<br>**Class Arachnida**<br>**Order Scorpiones**	All 1753 species described (14 families), apoikogenous, examples:	Viviparity: in the lumen of ovariuterine tubule.	[Epithelial vacuolation, fragmentation, and sloughing may be a common mechanism for nutrient transfer to embryo] (Farley, 1998: p. 203)	Absorption *via* integument; suggested transport of nutrients by amnion and serosa (Farley, 2001)	Francke (1982);<br>Sissom (1990);<br>Polis & Sissom (1990);<br>Lourenço (2002);<br>Farley (1998, 2001, 2011)
**Family Euscorpiidae**	*Scorpiops* sp.	Viviparity: in the lumen of ovariuterine tubule.	[Diffuse pseudoplacenta].		Sissom (1990)
**Family Vaejovidae**	*Vaejovis* sp.	Viviparity: in the lumen of ovariuterine tubule.	[Diffuse pseudoplacenta].		Sissom (1990)
	Smeringurus mesaensis [oocytes 0.1–0.2 mm, blastula 0.5–0.6 mm, embryo with metasoma not extended ~1.5 mm] (Farley, 1998);<br>embryo at birth 11.0 mm in length, weight 0.03 g]<br>(Farley, 1996)	Viviparity: in the lumen of ovariuterine tubule.	[Follicular placenta derived from follicular cells inside the protuberance] of [ovariuterine tubule] (Farley, 1996: p. 189)	[Nutrients are probably absorbed through the integument]<br>(Farley, 1996: p. 197).	Farley (1996, 1998, 2001, 2011)
**Family Buthidae**	*Centruroides sculpturatus* [suggested dry mass increased by at least 40-fold]	Viviparity: in the lumen of ovariuterine tubule.			Toolson (1985[2]);<br>Sissom (1990)

	(Toolson, 1985)				
	Lychas tricarinatus [common size of follicular ova 28.5 µm, largest ovum 54.0 µm; growth of the embryo in … early stages, 172.8 µm; advanced embryos 4.1 mm long, 2.0 mm broad]<br>(Mathew, 1960: pp. 222-223)	Viviparity: in the lumen of [ovarian tubes].		[Conical process (of extra-embryonic origin) terminating in a globular body; this structure pierces through… mesosoma and enters the midgut of the embryo; conical structure… secret(es) a nutritive substance at the globular free end, which is passed along distinct duct into the lumen of the midgut]<br>(Mathew, 1960: p. 222);<br>[The embryo mesosoma has a hollow dorsal stalk and globular body that apparently transfers nutrients from the maternal ovariuterine wall directly to the digestive tract of the embryo]<br>(Farley, 2011: p. 8)	Mathew (1960, 1962); Farley (2011)
	Katoikogenous, examples:	Viviparity: in diverticulae of ovariuterine tubules.	Wall of diverticula during the early developmental stages, and tubular appendix during later stages.<br>[At the tip of each diverticulum, a tubular appendix has morphology that suggests it absorbs and channels maternal nutrients to the mouth of the embryo]	[Trophamnion derived from [polar-body cells]; [when the mechanism for oral feeding is developed, the necessity for absorption is eliminated and the trophamnion desintegrates]<br>(Franke, 1982: p. 33);<br>[it appears that the	Franke (1982)<br>Polis & Sissom (1990);<br>Farley (1996, 2001, 2011)

			(Farley, 1996: p. 197); [the appendix is entirely maternal in origin, developing from large follicular cells] (Farley, 2011: p. 8)	trophamnion aids the embryo in absorbing maternal nutrients] from diverticulum (Polis & Sissom, 1990: p. 181);	
**Family Scorpionidae**	*Urodacus manicatus* [embryo with metasoma and sting rudiment 11.0 mm]	Viviparity: in diverticulae of ovariuterine tubules.		Absorption in early embryos, oral feeding in later embryos.	Mathew (1968)
**Order Pseudoscorpiones**	All 3385 species described (25 families)	Brooding: in brood sac.	Ovary in secretory phase.	Consuming nutritive fluid by pumping organ to embryonic gut; [osmotic uptake of part of the nutritive fluid] (Weygoldt, 1969: p. 68); additional matrophagy in *Paratemnoides nidificator*.	Weygoldt (1969); Tizo-Pedroso & Del-Claro (2005)
**Class Acari Family Spinturnicidae**	*Spinturnix vespertiliones* [ovarian oocytes 15.0 µm, early steroblastula 50.0 µm, full-grown embryo 670.0×500.0 µm] (Akimov & Yastrebtsov,	Viviparity: in ovary and haemocoel.	[Exotrophic nutrition at the expense of the female] (Akimov & Yastrebtsov, 1990: p. 9)		Akimov & Yastrebtsov (1990); Yastrebtsov (1992)

	1990)				
**Family Epidermoptidae**	*Knemidocoptes mutans*	Viviparity: in oviduct.	[Nutrition of embryo].	Consumption of [Exsudat (Mukoproteid?)] *via* body wall.	Langenscheidt (1958)
**Subphylum Crustacea**<br>**Class Branchiopoda**<br>**Order Anomopoda**<br>**Family Moinidae**	All 19 species described (2 genera)	Brooding: in carapace brood chamber.	[Nutrient-secreting Nährboden] (or 'placenta')] (Fryer, 1996: p. 1703); [placenta or "Nährboden" or] that supplies nourish-ment to the developing embryo] (Goulden, 1968: p. 9)		Goulden (1968);<br>Fryer (1996);<br>Egloff *et al*. (1997)
**Order Ctenopoda**<br>**Family Sididae**	*Penilia avirostris*	Brooding: in carapace brood chamber.			Egloff *et al*. (1997);<br>Negrea *et al*. (1999)
**Order Gymnomera**<br><br><br><br>**Family Polyphemidae**	All 37 species described (3 families); examples:<br><br>*Polyphemus pediculus* [five- to six-fold increase in size in… embryos] (Patt, 1947: p. 350)	Brooding: in carapace brood chamber.<br><br><br><br>Brooding: in carapace brood chamber.	[Nährboden].<br><br><br><br>[Nährboden… secrete(s) nutrient materials into the brood chamber for use by the developing embryos] (Patt, 1947: p. 344)		Weismann (1877);<br>Patt (1947[1]);<br>Fryer (1996);<br>Egloff *et al*. (1997)<br><br>Patt (1947[1])
**Family Cercopagididae**	*Bythotrephes longimanus*	Brooding: in carapace brood chamber.	[Nährboden=nurturing tissue]		Rossi (1980);<br>Alwes & Scholtz (2014)

	[with the development of embryos the brood chamber grows greatly, visibly bulging on the animal's back]<br>(Rossi, 1980: p. 30)		(Alwes & Scholtz, 2014: p. 3)		
**Family Podonidae**	*Podon schmackeri*<br>[embryo length…varied with the developmental stages of the embryos, ranged from 0.03–0.38 mm]<br>(Kim & Onbé, 1989: p. 56)	Brooding: in carapace brood chamber.			Kim & Onbé (1989)
	Evadne nordmanni<br>[egg 70.0 µm] (Platt & Yamamura, 1986);<br>[embryos ~80.0–680.0 µm] (Bainbridge, 1958)	Brooding: in carapace brood chamber.	[In *Podon* and *Evadne* spp., the eggs are nourished by the mother through the fluid bathing the embryos in the closed brood pouch via glandular cells in its wall] (Gieskes, 1970 in Platt & Yamamura, 1986: p. 138);<br>[nutrients of aborted embryos could be utilized by the remaining embryos]<br>(Platt & Yamamura, 1986: p. 138)		Bainbridge (1958);<br>Gieskes (1970) in Platt & Yamamura (1986);<br>Platt & Yamamura (1986)
**Class Malacostraca**<br>**Order Isopoda**<br>**Family Armadillidiidae**	*Armadillidium vulgare*	Brooding: in [marsupium].	Cotyledons;		Lawlor (1976[2]);

	[approximately twofold increase in dry weight of the young while in the marsupium] (Lawlor, 1976: p. 777)		[maternal providence] (Hoese & Janssen, 1989: p. 170) .		Hoese & Janssen (1989[1]); Helden & Hassall (1998); Surbida & Wright (2001); Hornung (2011)
	Schizidium tiberianum	Brooding: in sacs inside marsupium.	Epithelium of sac [secreting nutrients to the developing embryos] (Warburg & Rosenberg, 1996: p. 213)	Embryophagy= [intramarsupial cannibalism] (Warburg, 1994: p. 566)	Warburg (1994); Warburg & Rosenberg (1996[1])
**Family Armadilliidae**	*Armadillo officinalis*	Brooding: in sacs inside marsupium.	Epithelium of sac [secreting nourishment to the developing embryos] (Warburg & Rosenberg, 1996: p. 221)	As above.	Warburg (1994); Warburg & Rosenberg (1996[1])
**Family Porcellionidae**	*Porcellio olivieri*	Brooding: in marsupium.	Cotyledons.		Warburg & Rosenberg (1996[1])
	Porcellio scaber [young larvae length 0.5–0.6 mm, juveniles length 1.8 mm, width 0.6 mm] (Verhoeff, 1917)	Brooding: in marsupium.	Cotyledons [afford the storing place for mucous mass by which the embryos are bred during the course of development] (Akahira, 1956: p. 497); [brood is actively nourished by the maternal individual] (Hoese & Janssen, 1989: p. 171)	Histotrophy in early developmental stages, histophagy (proved in experiments with ink) in later stages.	Verhoeff (1917, 1920): embryonic growth by water uptake; Akahira (1956); Hoese & Janssen (1989[1])
	Porcellio ficulneus	Brooding: in marsupium.	Cotyledons.	Embryophagy= [intramarsupial cannibalism]; [apparently, the	Warburg (1994)

				cotyledonal fat is the source of nutrition for the marsupial brood] (Warburg, 1994: p. 564)	
	Porcellio dilatatis	Brooding: in marsupium.	[Amino acids or their derivatives have been transferred from the mother to the embryos through the maternal ventral integument] (Hoese & Janssen, 1989: p. 170)		Unpublished data of Fogarty[2] and Souty & Gohar[2] cited in Hoese & Janssen (1989)
**Family Ligiidae**	*Ligia oceanica* [36% difference in the dry weight of ova and young] (Saudray, 1954: p. 815); [mean dry weight per egg 301 (301±34.4) mg, per juvenile 310 (310±13.4) mg] (Pandian, 1972: p. 435)	Brooding: in marsupium.	[Embryos are nourished by maternal secretions] (Saudray, 1954: p. 815)		Saudray (1954[2]); Green (1965); Pandian (1972[2])
**Family Oniscidae**	*Oniscus asellus*	Brooding: in marsupium.	Cotyledons; [nutrition of incubated brood].	Histotrophy during early developmental stages, histophagy during the later stages.	Hoese & Janssen (1989[1])
**Family Hemioniscidae**	*Hemioniscus balani* [egg 65.0×50.0 µm] {embryo with leg rudiments 375.0×125.0 µm}	Brooding: [in internal incubating pocket].	[Embryonic nutrition].	[Extra-embryonic epithelial layer… plays the foremost role in… exogenous nutriment absorption] (Goudeau, 1977: p. 238)	Goudeau (1977[1])
**Family Chaetiliidae**	*Glyptonotus antarcticus*			Embryophagy and	Janssen & Hoese (1993[1])

				consumption of maternal secretions.	
**Family Gnathiidae**	*Paragnathia formica* [egg 200.0–300.0 µm in ø, stage 3 embryos ~1.0 mm long], {stage 3 embryos 1.5×0.155–0.41 mm} (Manship *et al*., 2011)	Viviparity: in paired uterine sacs.	[Monod (1926) suggested that the intimate contact between female and brood in *Paragnathia* facilitated the exchange of nutritive material] (Klapow, 1970: p. 367)		Monod (1926); Klapow (1970); Manship *et al*. (2011)
**Family Cyrolanidae**	*Excirolana chiltoni* [egg 0.94×0.60 mm, embryos increase threefold in volume] (Klapow, 1970: p. 363)	Viviparity: in paired uterine sacs.	[Intimate contact between female and brood might suggest that the embryos receive nutritive substances during gestation in addition to the initial yolk contained in the egg] (Klapow, 1970: p. 364)		Klapow (1970)
**Order Decapoda Family Hippolytidae**	*Chorismus antarcticus* [mean egg lipid content at spawning is 125.0 µg; mean embryo lipid content after 4 month of incubation is 200.0 mg; embryo mean dry weight by about 50.0 mg during period of lipid accumulation] (Clarke, 1985: p. 241)	Brooding: in marsupium.	[Female shrimp may be able to supply the developing eggs (i.e. embryos) with nutrient] (Clarke, 1985: pp. 241-242)		Clarke (1985)

**Subphylum Hexapoda**<br>**Class Insecta**<br>**Order Diptera**<br>**Family Glossinidae**	All 31 species described including:				Hardenberg (1929);<br>Hagan (1951);<br>Tobe & Langley (1978)<br>Meier *et al*. (1999);<br>Pellegrini *et al*. (2011)
	Glossina morsitans [egg 1.57±0.01 mm length, dry weight 0.086±0.001 mg; second instar 4.5 mm, dry weight 5.0 mg, third instar at parturition 6.0 mm, dry weight 10.0 mg] (S.E.M.)	Viviparity: in uterus.	Uterine accessory ‘milk’ glands;<br>[adenotrophic viviparity].	Ingestion of uterine secretions.	Denlinger & Ma (1974[2])
**Family Hippoboscidae**	All species (213 spp.), including:<br>*Melophagus ovinus* {egg 0.68×0.26 mm} (Saunders, 1964);<br>[fully grown larvae 3.5-3.7×1.9×1.6 mm] (Pratt, 1893)	Viviparity: in uterus.	As above.	As above.	Pratt (1893);<br>Hardenberg (1929);<br>Hagan (1951);<br>Saunders (1964);<br>Meier *et al*. (1999)
**Family Nycteribiidae**	All 274 species known.	Viviparity: in uterus.	As above.	As above.	Hardenberg (1929);<br>Hagan (1951);<br>Meier *et al*. (1999);
**Family Streblidae**	All 237 species known.	Viviparity: in uterus.	Accessory glands of the	As above.	Hardenberg (1929);

			uterus.		Hagan (1951);<br>Meier *et al*. (1999)
**Family Sarcophagidae**	*Sarcophaga nigriventris*	Viviparity: in uterus.	[Pseudo-placental oligolarviparity].		Meier *et al*. (1999)
**Family Calliphoridae Subfamily Mesembrinellinae**	Apparently all 30 species described.	Viviparity: in uterus.	['Milk' is produced by the spermathecae] (Meier *et al*., 1999: p. 205); [adenotrophic viviparity] (Guimarães, 1977); [pseudo-placental viviparity] (Meier *et al*., 1999)		Guimarães (1977);<br>Meier *et al*. (1999)
**Family Calliphoridae Subfamily Ameniinae**	*Amenia leonina albomaculata* [egg 1.9–2.4×0.5–0.6 mm, 1st instar larvae 3.1–7.0×0.6–1.3 mm, 2nd instar larvae 7.4×1.2 mm at larvaposition] (Ferrar, 1976)	Viviparity: in uterus.			Ferrar (1976);<br>Meier *et al*. (1999)
	Amenia chrysame [egg 1.7–1.9×0.5 mm, 1st instar larvae 1.9–4.0×0.5–0.7 mm, 2nd instar larvae 5.5×1.0 mm at larvaposition] (Ferrar, 1976)	Viviparity: in uterus.			Ferrar (1976);<br>Meier *et al*. (1999)

	Amenia imperialis dubitalis [egg 2.0–2.3×0.6 mm, 1st instar larvae 4.8–6.2×0.7–1.0 mm, 2nd instar larvae 6.7–7.4×1.0–1.1 mm at larvaposition] (Ferrar, 1976)	Viviparity: in uterus.			Ferrar (1976); Meier *et al*. (1999)
**Family Calliphoridae Subfamily Phumosiinae**	*Euphumosia papua* [egg 1.6–1.7×0.45–0.5 mm, large uterine larva 8.5×3.1 mm after fixation] (Ferrar, 1978)	Viviparity: in uterus.			Ferrar (1978); Meier *et al*. (1999)
**Family Calliphoridae Subfamily Luciliinae**	*Dyscritomyia fasciata* [egg 2.4×1.4×1.2–2.0 mm, living expelled larva 4.0×1.5 mm] (Pollock, 1974)	Viviparity: in uterus.			Pollock (1974); Meier *et al*. (1999)
**Family Muscidae**	*Dasyphora pratorum* [egg 2.5 mm, larva 6.0 mm]	Viviparity: in uterus.			Meier *et al*. (1999)
**Family Cecidomyiidae**	Apparently all 6 known species of the genus *Miastor*, examples:				

	Miastor metroloas<br>[1st instar larva at birth 1.2×0.2 mm]<br>(Wyatt, 1967)	Viviparity:_in haemocoel= [haemocoelous viviparity]<br>(Hagan, 1951)		[Embryos…develop at the expense of the fatbodies, muscles and surrounding tissues of the mother larva]<br>(Harris, 1923: p. 96); [thickened serosa absorbs nutriment from mother and makes it accessible to embryo] (Kahle, 1908: p. 51 in Hagan, 1951); larvae feed on maternal tissues and younger siblings (Hagan, 1951)	Kahle (1908) in Hagan (1951);<br>Harris (1923);<br>Nikolei (1961);<br>Wyatt (1967)
	Miastor castaneae<br>[egg 0.53×0.1 mm,<br>1st instar larva at birth 1.3×0.2 mm]<br>(Wyatt, 1967)	Viviparity: in haemocoel.			Wyatt (1967)
	Meinertomyia fasciata	Viviparity: in haemocoel.			Hagan (1951)
	Henria psalliotae<br>[within the hemipupa the embryos grow very rapidly]<br>(Wyatt, 1961: p. 135)	Viviparity: in haemocoel.		[The tissues of hemipupa are absorbed] (Wyatt, 1961: pp. 135-136)	Wyatt (1961)
	Heteropeza pygmaea<br>[in fully grown maternal larva the embryos are about 0.8 mm long]<br>(Wyatt, 1963: p. 137);<br>[the total volume increase of (the) egg during cleavage amounts	Viviparity: [floating in the haemocoel of the mother larva. …Embryos remain enveloped by the follicular epithelium during the whole of embryonic development]<br>(Junquera, 1984: p. 197)	[Degradation of the nurse chamber (nurse cells) contents into molecular components, which in turn might serve as a source of nutrients for the growing egg. …This would be additional to the	[As the embryos develop, the fat body and other organs are bsorbed until only the cuticle and tracheal system remain by the time the young escape]<br>(Wyatt, 1963: p. 137)	Nikolei (1961);<br>Wyatt (1963);<br>Junquera (1983, 1984);<br>Hodin & Riddiford (2000)

	to 200%] (Junquera, 1983: p. 306)		absorption of nutrients from the haemolymph (by follicular epithelium) (Junquera, 1984: p. 203); [since tight junctions (between follicle cells) were not found, these observations suggest that all the nutrients required for egg (=embryo) growth should be able to pass between the follicle cells, and thus be directly incorporated into the egg plasmodium] (Junquera, 1983: pp. 308-309)		
	Brittenia fraxinicola [1st instar larva at birth 1.4×0.15 mm] (Wyatt, 1967)	Viviparity: in haemocoel.			Wyatt (1967)
	Leptosyna nervosa [1st instar larva at birth 1.2×0.086 mm] (Wyatt, 1967)	Viviparity: in haemocoel.		[Embryos…develop within the (mother) haemocoele, gradually absorbing all organs except the well-developed tracheal system] (Wyatt, 1967: p. 83)	Wyatt (1967)
	Heteropezula tenuis [1st instar larva at birth 0.9×0.08 mm] (Wyatt, 1967)	Viviparity: in haemocoel.		[Developing embryos absorb the maternal fat body] (Wyatt, 1967: p. 90)	Wyatt (1967)

	Mycophila speyeri [paedogenetic mother larva produces 1-2 large embryos 1.2×0.2 mm, and, at the same time, few small mbryos 0.4 mm; by the fifth day, the embryos are fully grown and occupy most of the (maternal) body cavity] (Wyatt, 1964: p. 20)	Viviparity: in haemocoel.		[Larvae consume the histolyzing tissues of the mother] (Hodin & Riddiford, 2000: p. 359)	Wyatt (1964); Hodin & Riddiford (2000)
	Micophyla barnesi	Viviparity: in haemocoel.			Wyatt (1964)
	Micophyla nikoleii [newborn daughter larva 0.8 mm]	Viviparity: in haemocoel.			Nikolei (1961)
	Tekomyia populi {late embryos ~1.0 mm} (Nikolei, 1961)	Viviparity: in haemocoel.			Nikolei (1961); Wyatt (1961)
**Family Syrphidae**	*Eristalis tenax* {three daugther larvae completely fill the mother larva skin} (Ibrahim & Gad, 1975: fig. 1)	Viviparity.			Ibrahim & Gad (1975)
**Order Strepsiptera**	Apparently all species (~600 species, 8 families); example:	Viviparity: in haemocoel= [haemocoelous viviparity].		Embryonic [trophic membranes… (including trophamnion) serve as nutrient organs for the embryo; …embryo suspended in nutriment which simply passes by diffusion or osmosis	Hagan (1951)

	Elenchus tenuicornis [egg/early embryo 138,910 µm³, fully differentiated L1 larva 650,000 µm³]	[During organogenesis, the embryo remains in the remnants of the still existing follicle cells] (Büning, 1998: p. 7)	[During…developmental period, one can assume that nourishment of the embryos is *via* the hemolymph] (Büning, 1998: p. 7)	through trophic membrane to the egg (embryo)] from haemocoel (Hagan, 1951: pp. 231, 261)<br><br>[Fat body of the mother decreases] (Büning, 1998: p. 7)	Büning (1998)
**Order Dermaptera Family Hemimeridae**	Apparently all 10 known species of the genus *Hemimerus,* including *Hemimerus talpoides*	Viviparity: in ovary.	[Anterior and posterior maternal pseudoplacentae] formed by the follicular epithelium; pseudoplacental cavity seems to be filled with nutriment in its liquid form] (Hagan, 1951: p. 267); [pseudoplacental viviparity].	In early embryo [amnion actively engaged in the absorption of nutriments from the pseudoplacental cavity; trophocytes probably (have) come in contact with the anterior maternal pseudoplacenta, and its nutriment is obtained from this source] (Hagan, 1951: p. 269); in mid-stage embryo – [tropamnion and trophoserosa; in later embryos [amniotic and serosal fetal pseudo-placentae]	Heymons (1912); Hagan (1951)

				(Hagan, 1951: pp. 271, 273)	
**Family Arixeniidae**	*Arixenia esau*	Viviparity: in a follicle and lateral oviducts=uteri.	[Follicular cells] during the early stages of the development; [epithelial cells of the uterus] during the later stages of the development; [embryos… are sequentially nourished …by two separate populations of epithelial cells] (Tworzydlo *et al*., 2013: p. 5); [pseudoplacento-uterotrophic viviparity]		Tworzydlo *et al*. (2013)
	Xeniaria jacobsoni	Viviparity: in ovary.	Follicular epithelium; [pseudoplacental viviparity].	[Serosa becomes a thickened trophic organ] underlying follicular epithelium (Hagan, 1951: p. 290)	Hagan (1951)
**Family Labiidae**	*Chaetospania borneensis*	Viviparity: in uterus.			Kočarek (2009)
**Order Blattoidea Family Blaberidae**	*Diploptera punctata* [oocyte length 1.5 mm, embryo length at parturition 6.0 mm, both wet and dry weight increase fifty-fold from oviposition to parturition] (Stay & Coop, 1973: p. 160); [at birth the first instar	Viviparity: in uterus (=brood sac).	[Brood sac…is a typical insect integumentary gland which secretes a 'milk'-containing protein and carbohydrate to nourish the developing embryos; secretory cells of the brood sac wall produce a nutrient fluid for the developing embryos] (Stay & Coop, 1974: pp.	[Pleuropodial function is thought to be one of nutrition… (*via*) diffusion, or transfer of …both liquids and gases] (Hagan, 1951: p. 345); [embryos drink protein-rich milk] (Youngsteadt *et al*., 2005: p. 804)	Hagan (1951); Roth & Willis (1955, 1957); Roth & Hahn (1964); Stay & Coop (1973, 1974²); Roth (1970, 1989); Williford *et al*. (2004); Youngsteadt *et al*. (2005)

	larvae are about 50 times heavier than the eggs at oviposition; increase in embryo weight is paralleled by increases in protein, carbohydrate, lipid and uric acid and accompanied by a 60-fold increase in embryo volume] (Stay & Coop, 1974: p. 669)		669, 688); [embryo in the maternal genital tract obtains at least part of its nutriment by means of a pseudoplacenta which, in the free-embryo type, is wholly of embryonic origin. Contact of egg, pseudoplacenta, or embryo with the maternal genital tract is maintained only by pressure or proximity] (modified from Hagan, 1951 by Roth & Willis, 1957: p. 227); [pseudoplacental viviparity]; if placentotrophy occurs, it can only exist at early developmental stages.		
**Order Psocoptera Family Archipsocidae**	Apparently all 4 species in the genus *Archipsocopsis,* including *Archipsocopsis fernandi* [egg 57.6×37.2 μm] (Fernando, 1934)	Viviparity: in ovarian tube.	Wall of ovarian tube; [embryonic nutrition; pseudoplacental viviparity].	[Food material is obtained through the serosa; Fine cytoplasmic processes pass from the cells of the serosa into the wall of the ovarian tubule wherever the embryo comes in contact with it, and this is undoubtedly a simple means of obtaining nourishment; serosa acts	Fernando (1934); Hagan (1951)

				as a temporary organ for obtaining nourishment for the embryo from the wall of the ovarian tubule] (Fernando, 1934: pp. 111, 119);<br>[fusion of serosa with ovarian tubule; serosa be-comes distinctly trophic] (Hagan, 1951: p. 353)	
**Order Hemiptera**<br>**Family Aphididae**	Parthenogenetic females in apparently all 5001 described species, examples:<br>*Byrsocrypta gallarum*	Viviparity: in ovariole.	Follicular epithelium; [pseudoplacental viviparity].	[Probable trophic function of the serosa] (Hagan, 1951: p. 356)	Hagan (1951); Bermingham & Wilkinson (2009)
	Aphis sambuci	Viviparity: in ovariole.	[Pseudoplacental viviparity].		Hagan (1951)
	Uroleucon tanaceti [egg 30.0×24.0 µm, after first cleavage 43.0×23.0 µm, embryo ready for birth 840.0×264.0×240.0 µm]	Viviparity: in ovariole.	[Pseudoplacental viviparity].		Hagan (1951)
	Drepanosiphum platanoides [parthenogenetic oocyte 30.0 µm], {non-mature embryo 250.0×110.0 µm}	Viviparity: in ovariole.	[Pseudoplacental viviparity].		Büning (1985)
	Brevicoryne brassicae	Viviparity: in ovariole.	Thickened ovariole wall; [pseudoplacental viviparity].	Serosa.	Couchman & King (1980[1])

	Acyrthosiphon pisum [early embryo length 400.0 μm, mature embryo prior larviposition 1 mm long] (Miura *et al*., 2003: p. 76)	Viviparity: in ovariole.	[Pseudoplacental viviparity].		Miura *et al*. (2003)
	Myzus pescicae	Viviparity: [in maternal haemocoel].	[Thymidine can pass into the embryos so quickly… (that) it seems likely that active uptake of amino-acids and other nutrients takes place from the maternal haemocoel] (Blackman, 1974: p. 1137)		Blackman (1974²)
**Family Polyctenidae**	Apparently all 32 species described;<br>example:	Viviparity: in oviducts.			Hagan (1931, 1951)
	Hesperoctenes fumarius [fully mature egg 125.0×48.0 μm, length of the oldest embryo ready for birth 1.3 mm] (Hagan, 1931)	Viviparity: in oviducts.	Wall of oviduct; [pseudoplacental viviparity].	In early embryo [thickened serosa]; in later embryo [nutritive function has been ascribed to the pleuropodial sheath] (Hagan, 1951: p. 410)	
**Order Coleoptera**<br>**Family Chrysomelidae**	*Oreina gloriosa* {egg 0.6×0.21 mm, larva 2.17×0.91 mm} (Bontems, 1984); [l'enorme accroissement de taille des larves] (Bontems, 1984: p. 978)	Viviparity: in oviducts.	[Retention of eggs in oviducts combined with matrotrophy by diffusion of nutrients through a reduced chorion] (Dobler *et al*., 1996: p. 2383)	[A la diffusion probable de substances nutrives] *via* chorion (Bontems, 1984: p. 978)	Bontems (1984); Dobler *et al*. (1996)

	Oreina ganglbaueri {egg 0.56×0.26 mm, larva 3.73×1.13 mm}	Viviparity: in ovarioles.		As above.	Bontems (1984)
	Oreina alpestris {egg 0.56×0.21 mm, larva 2.56×0.86 mm}	Viviparity: in ovarioles.		As above.	Bontems (1984)
	Oreina cacaliae {egg 1.04×0.39 mm, larva 1.47×0.60 mm}	Viviparity: in ovarioles.		As above.	Bontems (1984)
	Oreina bifrons	Viviparity: in oviducts.	[Retention of eggs in oviducts combined with matrotrophy by diffusion of nutrients through a reduced chorion] (Dobler *et al*., 1996: p. 2383)		Dobler *et al*. (1996)
	Oreina variabilis	Viviparity: in oviducts.	As above.		Dobler *et al*. (1996)
	Oreina speciosa {egg 0.43×0.17 mm, larva 2.26×0.78 mm} (Bontens, 1984)	Viviparity: in oviducts.	As above.		Bontens (1984); Dobler *et al*. (1996)
	Oreina virgulata	Viviparity: in oviducts.	As above.		Dobler *et al*. (1996)
	Oreina melanocephala	Viviparity: in oviducts.	As above.		Dobler *et al*. (1996)
	Oreina intricata	Viviparity: in oviducts.	As above.		Dobler *et al*. (1996)
	Agrosteomella chinensis	Viviparity: in ovarioles.			Bontems & Lee (2008)
	Gonioctena pallida	Viviparity: in uterus.	[Eggs in a uterus…are nourished by placenta-like structures] (Selman, 1994: p. 69); [pseudoplacental viviparity].		Selman (1994)
	'*Platyphora quadrisignata*'	Viviparity: in uterus.			Schroder *et al*. (1994)
**Family Tenebrionidae**	*Alegoria castelnaui*	Viviparity: in vagina.			Dutrillaux *et al*. (2010)

	[egg 4.0×1.0 mm], {larva 5.75×1.04 mm}				
**Family Micromalthidae**	*Micromalthus debilis* {ovarian parthenogenetic eggs 0.6×0.09 mm, embryos 0.89×0.17 mm in thelytocous female larva}	Viviparity: in haemocoel.			Scott (1938)
**<u>Phylum Onychophora</u>**<br>**Family Peripatidae**	Entire family (70 species), except lecithotrophic viviparous genera *Eoperipatus* and *Typhloperipatus*; examples:	Viviparity: in uterus.	Specialized areas of the uterine wall (placentas); [placental/placentotrophic viviparity].	Embryos with stalk with one exception.	Anderson (1973); Mayer *et al*. (in press)
	Peripatus acacioi {blastula 230.0 µm}, [round embryo cavities with early embryos up to coiled stages 2.0–4.0 mm, elongated embryo cavities with flexed embryos 4.0-7.0 mm in length, fetuses 8.0–15.00 mm] (Campiglia & Walker, 1995)	Viviparity: in uterus.	Epithelial sac and its syncytial area [placenta] formed by maternal uterine epithelium; [while the embryo is retained within the embryo sac, nutrients are supplied by the placenta. The presence of microvilli on both sides of the placenta/epithelial sac, indicates a probable active mechanism for uptake of material from the maternal haemocoel for utilization by the developing embryo.	Embryo sac of embryonic origin; [during the early stages of segment formation the embryo presumably absorbs material *via* its body surface. The barbed projections at the ends of the walking legs are assumed to increase the surface area for nutrient uptake by the embryo at this stage]; during later developmental stages [in the straightened fetus nutrients are taken in	Walker & Campiglia (1988[1], 1990[1]); Campiglia & Walker (1995[1])

			Material may be stored in the placenta and then released into the fluid-filled cavity surrounding the developing embryo, where it is retained within the embryo sac] (Campiglia & Walker, 1995: p. 196)	through the open mouth and are absorbed by the gut] (Campiglia & Walker, 1995: p. 196)	
	Plicatoperipatus jamaicensis [embryos … showed more than a twofold difference in weight] (Havel *et al*., 1989: p. 229)	Viviparity: in uterus.			Havel *et al*. (1989)
	Epiperipatus imthurni [youngest embryo 0.40 mm in ø, fully formed embryo, …is often an inch long] (Sclater, 1888: p. 346)	Viviparity: in uterus.			Sclater (1888); Anderson (1973)
	Epiperipatus biolleyi	Viviparity: in uterus.	[The lumen of the uterus is densely lined with microvilli. An increasing vacuolation causes an enlargement of the (uterine) prismatic epithelium, and reduction of the lumen. …This epithelial enlargement is produced by an enormous expansion of the vacuoles, which displaces the	[The embryos bear a microvilli border and are surrounded by a conspicuous noncellular coat of 0.8 mm hickness, which is interspersed by numerous vesicles of different size. … The dense microvillous lining of the secretory cells and embryonic cells	Brockmann *et al.* (1999[1])

			cytoplasm to narrow margins… The second secretory cell layer… overlies the uterine epithelium. ...Apically (its cells) bear numerous microvilli. These cells develop a secretory product which is located apically in the cells as prominent ` mucus buds'. …Its location surrounding the cleavage embryo and its secretory function suggests a role in providing nutrients for the early embryonic development. …The vacuolated uterine epithelium adjacent to each embryo may function as transport epithelium, as suggested by the presence of infolded basal laminae, abundant mitochondria, and apical microvilli] (Brockmann, *et al.* 1999: pp. 343, 348)	presumably have secretory and absorbtive functions, respectively.] (Brockmann, *et al.* 1999: pp. 345, 348)	
	Epiperipatus isthmicola	Viviparity: in uterus.			Mayer & Whitington (2009)
	Epiperipatus trinidadensis [eggs 0.04 mm in ø, embryo 22.0 mm long]	Viviparity: in uterus.	[Outer and inner placental rings] formed by the external and internal epithelial layers of the	Embryo sac of embryonic origin.	Anderson *et al*. (1972); Anderson (1973); Kaestner (1980) in Havel *et al*. (1989);

	(Kaestner, 1980 in Havel *et al*., 1989); [largest embryos found 15.0–40.0 mm] (Anderson *et al*., 1972)		oviduct.		Ruhberg (1990); Campiglia & Walker (1995[1])
	Macroperipatus torquatus	Viviparity: in uterus.			Anderson *et al*. (1972); Campiglia & Walker (1995[1])
**Family Peripatopsidae**	Preliminarily 16 species, but more species with matrotrophy is expected from the genera *Peripatopsis*, *Opisthopatus* and *Euperipatoides*	Viviparity: in uterus.	Epithelium of oviduct; [nourishment is derived from the oviduct] (Manton, 1949: p. 498); [matrotrophic viviparity], [non-placental matrotrophy].	In some species: [yolk sac=trophic vesicle …serves as absorptive organ] (criticized by Manton, 1949: p. 538)	Manton (1949); Anderson *et al*. (1972); Anderson (1973); Ruhberg (1990); Mayer *et al*. (*in press*)
	Euperipatoides rowelli [dry weights of ‘developed’embryos were 10% higher than those of ‘undeveloped’ ones] (Sunnucks *et al*., 2000: p. 459)	Viviparity: in uterus.	[Combined lecithotrophic/ matrotrophic] viviparity (Mayer *et al*., *in press*)		Sunnucks *et al*. (2000); Mayer *et al*. (*in press*)
	Peripatoides novaezealandiae [yolky egg 1.5 mm in length] (Anderson, 1973); [early embryo with yolk sac: mean length 1.79 (±0.02) mm, mean	Viviparity: in uterus.	Presumably [combined lecithotrophic/matrotrophic] viviparity.		Anderson (1973); Tutt *et al*. (2002)

	weight 0.99 (±0.03) mg, late embryo similar in appearance to free-living individual: mean length 10.20 (±0.15) mm, mean weight 10.63 (±0.47) mg] (Tutt *et al*., 2002)				
	Peripatopsis sedgwicki [egg from the oviduct 260.0×80.0 µm; in late embryonic stages …the outer membrane fits tightly round the everenlarging young reaching sizes of] about 5.0 mm, intermediate between 6.0–7.0 and 3.0–4.0 mm (Manton, 1949: pp. 489, 493)	Viviparity: in uterus.		Trophic vesicle [swollen sac of dorsal extra-embryonic ectoderm] (Anderson, 1973: p. 4-16)	Manton (1949); Anderson (1973)
	Peripatopsis balfouri [egg from the oviduct 480.0×220.0 µm; In late embryonic stages …the outer membrane fits tightly round the everenlarging young, reaching sizes of appro-ximately 3.0 to 4.0 mm in the longer ø] (Manton, 1949: pp. 489, 493)	Viviparity: in uterus.			Manton (1949)
	Peripatopsis moseleyi	Viviparity: in uterus.		Trophic vesicle [swollen	Manton (1949);

	[egg from the oviduct 520.0×160.0 µm; in late embryonic stages …the outer membrane fits tightly round the everenlarging young, reaching sizes of approximately 6.0 to 7.0 mm in the longer ø] (Manton, 1949: pp. 489, 493)			sac of dorsal extra-embryonic ectoderm] (Anderson, 1973: p. 4-16)	Anderson (1973)
	Peripatopsis capensis [the embryo now grows considerably in length] (Sedgwick, 1885: p. 356); [egg from the oviduct 600.0×145.0 µm; in late embryonic stages …the outer membrane fits tightly round the everenlarging young reaching sizes of…abo-ut 5.0 mm, intermediate between 6.0-7.0 and 3.0-4.0 mm] (Manton, 1949: pp. 489, 493)	Viviparity: in uterus.			Sedgwick (1885); Manton (1949); Walker (1995)
	Peripatopsis leonina	Viviparity: in uterus.			Reid (1996)
	Peripatopsis clavigera [eggs ready for fertilization 110.0-150.0 µm], {early segmented	Viviparity: in uterus.		Trophic vesicle [of dorsal extra-embryonic ectoderm] (Hofmann, 1988: p. 256)	Reid (1996); Hofmann (1988)

	embryo 1.28 mm} (Hofmann, 1988)				
	Opisthopatus cinctipes [early cylindrical embryo 200.0–250.0 µm, embryo length at birth 3.0–4.0 mm] (Walker, 1992); {early elongating embryo 1.15 mm, embryo with all segmental appendages completed 3.75 mm} (Walker, 1995)	Viviparity: in uterus.			Reid (1996); Walker (1992, 1995)
	Opisthopatus roseus	Viviparity: in uterus.			Mayer *et al*. (*in press*)
	Metaperipatus inae [mature oocytes 50.0–100.0 µm], {embryo with trophic vesicle 1.9 mm} (Mayer, 2007)	Viviparity: in uterus.		Trophic vesicle.	Mayer (2007); Mayer *et al*. (*in press*)
	Metaperipatus blainvillei	Viviparity: in uterus.			Reid (1996)
	Paraperipatus amboinensis	Viviparity: in uterus.		Trophic vesicle.	Pflugfelder (1948); Mayer *et al*. (*in press*)
	Paraperipatus novaebritanniae	Viviparity: in uterus.			Reid (1996)
	Paraperipatus papuensis	Viviparity: in uterus.		Trophic vesicle.	Reid (1996); Mayer *et al*. (*in press*)
	Paraperipatus keiensis	Viviparity: in uterus.			Reid (1996)

**<u>Phylum Nematoda</u>**<br>**Class Enoplea**<br>**Order Muspiceida**<br>**Family Robertdollfusidae**	*Robertdollfusa paradoxa*<br>[egg 0.025 mm, variation of intrauterine larvae in size 0.890–0.915×0.0073 mm]	Viviparity: in uterus.			Chabaud & Campana (1950)
**Order Mermethida**<br>**Family Tetradonematidae**	*Heterogonema ovomasculus*,<br>eggs and intrauterine larvae show big difference in size.	Viviparity: in uterus.			Van Waerebeke & Remillet (1973)
**Family Mermethidae**	*Heleidomermis vivipara*<br>[eggs 0.030 mm, intrauterine larvae 0.240–0.250× 0.009–0.010 mm]	Viviparity: in uterus.			Rubzov (1972)
**Class Chromadorea**<br>**Order Rhabditida**<br>**Family Cosmocercidae**	*Cosmocercoides dukae*<br>[eggs 0.057×0.038 mm, larvae after hatching nearly double in length to the time of the 1st moult *in utero*, after	Viviparity: in uterus and pseudocoel.		Matrophagy.	Ogren (1953); Anderson (1960); Skrjabin *et al*. (1961)

	1st moult the 2nd stage rhabditiform larvae has length 0.697–0.714× 0.234–0.273 mm; some larvae transform to filariform stage and moult second time, reaching 0.748×0.03 mm when all larvae had matured a female was motionless and nearly devoid of internal structure except the oesophagus] (Ogren, 1953: p. 90); [eggs 0.08×0.05 mm, 1st-stage newly hatched larva 0.270×0.022 mm, moulting 1st-stage larva 0.400×0.022 mm, early 2nd-stage larva 0.480×0.029 mm, moulting 2nd-stage larva 0.614×0.028 mm, newborn 3rd-stage larvae 0.752–0.810× 0.026–0.028 mm] (Anderson, 1960; Skrjabin *et al*., 1961]				
	Probstmayria vivipara [larvae hatch and grow *in utero* to the 3rd stage reaching a half or more	Viviparity: in uterus.			Probstmayr (1865); Ransom (1907); Anderson (2000); Skrjabin *et al*. (1961)

	of the length of female, 1.48–1.59×0.04–0.10 mm] (Probstmayr, 1865); [eggs 0.058×0.040 to 0.100×0.075 mm according to the stage of embryonic development, larvae 1.8 mm or more] (Ransom, 1907; Anderson, 2000; Skrjabin *et al*., 1961)				
**Family Pharyngodonidae**	*Gyrinicola japonica* [eggs 87.0–96.0×48.0–54.0 µm, subadult males and embryos present *in utero*] (Planade *et al*., 2008)	Viviparity: in uterus.			Yamaguti (1938); Skrjabin *et al*. (1961); Planade *et al*. (2008)
	Gyrinicola chabadamsoni [eggs 80.0–100.0×30.0–60.0 µm, subadult males and embryos present *in utero*]	Viviparity: in uterus.			Planade *et al*. (2008)
**Family Atractidae**	*Atractis cruciata* [eggs 0.18–0.19×0.062–0.079 mm, intrauterine larvae in female 2.15–2.37×0.12 mm,	Viviparity: in uterus.			Skrjabin *et al*. (1964)

	reaching one third of female length]				
	Atractis emilii [eggs 0.070–0.077× 0.045–0.046 mm, intrauterine larvae in female 0.35–0.37×0.022–0.026 mm]	Viviparity: in uterus.			Skrjabin *et al*. (1964)
	Nouvelnema cyclophoron [very large intrauterine larvae, 1 mm, reaching two-thirds of the female length]	Viviparity: in uterus.			Skrjabin *et al*. (1964)
	Crossocephalus viviparous [initial egg size 0.20×0.11–0.13 mm, increasing with a morula inside to 0.32×0.21 mm and 0.40×0.22 mm, embryo reaches about half of the female length: 3.16×0.20 mm, 4.0×0.175 mm]	Viviparity: in uterus.			Skrjabin *et al*. (1964)
**Family Molineidae**	*Ollulanus tricuspis* [larval size after first moult 0.35×0.022 mm, after second moult 0.34 mm] (Cameron, 1927); [the entire larval	Viviparity: in uterus.			Cameron (1927); Chitwood & Christie (1940); Skrjabin *et al*. (1954); Anderson (2000)

	enlargement: 1st-stage larva 0.35×0.022 mm, 3rd-stage larva 0.4 mm that is almost a half length of female] (Anderson, 2000; Skrjabin *et al.*, 1954; Chitwood & Christie, 1940)				
**Family Camallanidae**	*Camallanus cotti* [eggs 0.03 mm, larvae 0.45–0.48×0.021–0.025 mm] (Ivashkin *et al.*, 1977); [larvae 0.390–0.450× 0.015–0.018 mm] (Moravec *et al.*, 2003); [eggs 0.033–0.048× 0.029–0.041 mm, larvae 0.240–0.336 mm] (Menezes *et al.*, 2006)	Viviparity: in uterus.			Ivashkin *et al*. (1977); Moravec *et al*. (2003); Menezes *et al*. (2006)
	Camallanus oxycephalus [length of intrauterine larvae 0.10–0.12 mm] (Ivashkin *et al.*, 1977); {egg ø 0.037 mm}, [newborn larvae from the same female 0.629–0.645 mm] (Stromberg & Crites, 1974)	Viviparity: in uterus.			Stromberg & Crites (1974); Ivashkin *et al*. (1977) ; Anderson (2000)
**Family**					

**Dracunculidae**	*Dracunculus medinensis* [eggs 0.035×0.025 mm] (Muller, 1970); [1st-stage released larvae 0.581–0.635× 0.010–0.024 mm] (Moorthy, 1938); [1st-stage intrauterine larvae 0.300–0.429×0.06 mm] (Ivashkin *et al*., 1977)	Viviparity: in uterus.			Moorthy (1938); Muller (1970); Pardanani & Kothari (1971); Ivashkin *et al*. (1977)
**Family Micropleuridae**	*Micropleura indica* [eggs 0.05–0.08×0.04 mm, larvae 0.72–0.87×0.020 mm] (Ivashkin *et al*., 1977)	Viviparity: in uterus.			Ivashkin *et al*. (1977)
**Family Philometridae**	*Philometra ovata* [thin-walled eggs: rounded non-segmented 0.030–0.035 mm, with 8-32 blastomeres 0.035–0.040 mm, oblong, with sickle-shaped embryos 0.040–0.052 mm, intrauterine larvae 0.4–0.5 mm] (Ivashkin *et al*., 1977); [intrauterine larvae 0.495–0.540×	Viviparity: in uterus.			Ivashkin *et al*. (1977); Moravec (1986, 1994)

	0.018–0.021 mm; 0.350–0.540 mm] (Moravec, 1986; Moravec, 1994)				
	Philometroides masu [eggs 0.040 mm, intrauterine larvae 0.750×0.03 mm]	Viviparity: in uterus.			Ivashkin *et al*. (1977)
	Philometroides nodulosa [eggs 0.012×0.014 mm, intrauterine larvae 0.316×0.035 mm] (Thomas, 1929; Ivashkin *et al*., 1977)	Viviparity: in uterus.			Thomas (1929); Ivashkin *et al*. (1977)
**Family Onchocercidae**	*Brugia pahangi* [the living embryos became further elongated within their large egg shell] (Rogers *et al*., 1976: p. 254)	Viviparity: in uterus.	[Uterine wall… contains large, actively-sectering apocrine cells…it is greatly thickened and the embryos adjacent to the wall have either burrowed into it, or the cells of the uterine wall have extended along the channels between the shells] (Rogers *et al*., 1976: p. 254)	[Egg shell acts as a placenta for passage of nutrients from uterine wall; …'nutrient channels' between adjacent egg shells acting as umbilical cords] (Rogers *et al*., 1976: p. 255)	Rogers *et al*. (1976[1])
	Dipetalonema viteae	Viviparity: in uterus.	[Apocrine secretion from the uterine wall; …uterine wall developed extensive system of microvilli presumably to aid nutrition of embryos] (Ellis *et al*., 1978: pp. 8-9)	['Nutrient channels' containing the uterine secretions] (Ellis *et al*., 1978: p. 7)	Ellis *et al*. (1978[1])

	Dirofilaria immitis	Viviparity: in uterus.	[Microvilli of uterine wall, amorphous substance between cleaving ova and uterine wall suggested a nutritional role] (Harada *et al*., 1970 in Ellis *et al*., 1978: p. 9);<br>[placental form of nutrition] (Harada *et al*., 1970 in Rogers *et al*., 1976 : p. 255)	[Papillary projections of fertilization membrane around ovum] (Harada *et al*., 1970 in Ellis *et al*., 1978: p. 9)	Harada *et al*. (1970[1])<br>Rogers *et al*. (1976[1]);<br>Ellis *et al*. (1978[1])
	Setaria cervi	Viviparity: in uterus.	[Microvilli-like structures on the uterine wall; amorphous substance] between embryos and uterine wall (Kagei, 1960 in Ellis *et al*., 1978: p. 9)	As above.	Kagei (1960[1]);<br>Ellis *et al*. (1978[1])
	Setaria digitata	Viviparity: in uterus.		[Interconnections of developing embryos and their connection with uterine wall, …are likely to function as 'nutritive channels'] (Decruse & Raj, 1990: pp. 103, 111)	Decruse & Raj (1990)
	Onchocerca volvulus	Viviparity: in uterus.		[Nutrient channels] (Prüsse *et al*., 1985: p. 216)	Prüsse *et al*. (1985);<br>Schulz-Key (1988);<br>Decruse & Raj (1990)
**Family Entaphelenchidae**	*Praecocilenchus rhaphidophorus* [juveniles reach… sexual maturity within the uterus of the living mother worm; in older	Viviparity: in uterus.			Poinar (1969);<br>Hunt (1993);<br>Ryss (2007)

	females newly formed adults were active and appeared to be mating within the uterus] (Poinar, 1969: pp. 227, 231)				
**Family Sphaerulariidae**	*Scatonema wülkeri* [intrauterine males grow from 0.127 to 0.350×0.018 mm, females grow from about the same size as males to 0.320×0.017 mm; progeny can reach full maturity and even copulate (and produce fully developed larvae) inside the maternal uterus] (Bovien, 1932: p. 28, 30)	Viviparity: in uterus.			Bovien (1932);<br>Poinar (1969)
**Family Iotonchiidae**	*Skarbilovinema lyoni* [eggs 40.0–65.0×30.0–50.0 µm, 4th stage female juvenile length 1150.0–1710.0 mm; anterior part of body of adult females contains eggs and juveniles of first- and second-stages, and posterior part contains third- and	Viviparity: in pseudocoel.			Zakharenkova & Chizhov (1991);<br>Chizhov *et al*. (2012)

	fourth-stage juveniles, males and, occasionally, copulating individuals] (Chizhov *et al*., 2012: p.				
	Skarbilovinema laumondi [eggs 40.0–68.0×30.0–52.0 µm, 4th stage female juvenile length 1770.0–2510.0 mm; posterior part of [female's] body and uterus area contain juveniles of third and fourth stages, males, infective females and occasionally copulating individuals] (Chizhov *et al*., 2012: p. 146)	Viviparity: in pseudocoel.			Chizhov *et al*. (2012)
**Family Mesidionematidae**	*Mesidionema praecomasculatis* [thick-walled (early) eggs 55.0–65.0×85.0–97.0 mm; thin-walled (embryonated) eggs 0.108×0.220 mm]	Viviparity: in uterus.			Poinar (1978)
**Family Anguinidae**	*Anguina tritici* [larvae grow *in utero* to the 2nd stage]	Viviparity: in uterus.			Gupta & Swarup (1968)
**Family**					

**Monhysteridae**	*Geomonhystera cf. disjuncta* large variation of intrauterine larvae in size in the same female	Viviparity: in uterus.			Tchesunov (2006)
**Family Rhabditidae**	*Caenorhabditis elegans*	Facultative viviparity: in uterus and body cavity.		Oophagy and matrophagy: [juveniles at hatching feed on (unfertilized) eggs and grow in the uterus; (further they) tear apart the walls of this organ and spread into the general body cavity. They then disrupt and devour the viscera of their mother] (Maupas, 1900: p. 22)	Maupas (1900); Chen & Caswell-Chen (2004)
	Phasmarhabditis hermaphrodita	Viviparity: in uterus and body cavity.		Oophagy and matrophagy: [The juveniles then find their food in the uterus at the expense of the unfertilized shell-less eggs which start to arrive. They grow in this enclosed environment and finally tear apart the walls by wriggling about. They then spread into the general body cavity and attack the viscera of their mother. The latter perishes being devoured from the interior by its children. Only the cuticle remains, forming a long transparent bag, in	Maupas (1900)

				which the young can be seen to move about. At least half of the females end thus] (Maupas, 1900: p. 39)	
**Family Diplogasteridae**	*Pristionchus robustus*	Viviparity: in uterus and body cavity.		Matrophagy: [towards the end of the fertile period, intra-uterine hatchings become frequent, and many mothers die being devoured by their progeny] (Maupas, 1900: p. 92)	Maupas (1900)
**Family Panagrolaimidae**	*Panagrellus redivivus* {non-embryonated thin-shelled eggs 0.042–0.048× 0.028–0.031 mm, egg with fully formed larva 0.061×0.044 mm, larvae 0.285–0.408× 0.015–0.019 mm}	Viviparity: in uterus.			Hechler (1970)
<u>**Phylum Loricifera**</u> **Family Urnaloricidae**	*Urnaloricus gadi* {early embryo 75.0×65.3 µm}, [Higgins larva 145.0×33.0 µm]	Viviparity: in pseudocoel; [viviparous pedogenesis]: Higgins larvae develop from eggs inside the ghost-larva.		[Embryos (develop) into Higgins-larvae while reabsorbing all the tissue of their maternal stage, the ghost-larva] (Heiner & Kristinsen, 2009: p. 129)	Heiner & Kristinsen (2009)

**Phylum Echinodermata**<br>**Class Ophiuroidea**<br>**Family Ophiuridae**	*Ophionotus hexactis*<br>[egg 0.2 mm juvenile disk 8.0 mm, arm length 20.0 mm] (Mortensen, 1921);<br>[egg 0.2 mm, pre-metamorphosed embryo 0.48 mm] (Hendler, 1975);<br>[egg 200.0 µm, juvenile disc ø 0.25–6.00 mm, total dry weight increase up to 2200-fold] (Turner & Dearborn, 1979)	Viviparity: in ovary =[ovarian sac].	[Nutrient transfer, supplemental nutrition]	Resorbing eggs [serve as nourishment for the young] (Mortensen 1921: p. 179);<br>[ingestion of parental body fluids for supplemental nutrition] (Turner & Dearborn, 1979: p. 49)	Mortensen (1921);<br>Hendler (1975);<br>Turner & Dearborn (1979[2]);<br>Khanna & Yadav (2005)
	Stegophiura sculpta<br>[egg 0.14 mm, pre-metamorphosed embryo 0.36 mm] (Hendler, 1975);<br>[disc ø in largest brooded young 1.1. mm] (Murakami, 1941)	Brooding: in bursae.		Embryophagy: [young larva (was) swallowed by an older young]<br>(Murakami, 1941: p. 68)	Murakami (1941);<br>Hendler (1975)
**Family Ophiacanthidae**	*Ophiomitrella conferta*	Brooding: in bursae.		Embryophagy [cannibalism].	Murakami (1941);<br>Hendler (1975)
	Ophiacantha vivipara<br>[smallest intrabursal juveniles: disk ø 0.67 mm, arm length 0.49 mm; largest	Brooding: in bursae.			Ludwig (1899);<br>Hendler & Tran (2001)

	intrabursal juvenile: disk ø 2.23 mm, arm length 2.86 mm] (Ludwig, 1899); [egg 0.6 mm, free juveniles on a parent 1.5 mm, and some brooded embryos up to 2.5 mm in disk ø] (Hendler & Tran, 2001)				
	Ophiochondrus stelliger	Brooding: in bursae.			Mortensen (1936)
**Family Ophionereididae**	*Ophionereis olivacea* [egg 400.0 µm, juvenile disc ø at birth 480.0 µm]	Brooding: in bursae.	[Ovoviviparity] =lecithotrophic viviparity.	[Metamorphosed embryos orient with their mouths against the bursal wall] (Byrne, 1991: p. 393)	Byrne (1991)
**Family Amphiuridae**	*Amphiura carchara* [oocyte mean maximum ø 0.45 mm (up to 1.28 mm), earliest embryos length 0.3 mm, late embryo disk ø 1.3 mm]	Brooding: in bursae.	Development [may be matrotrophic] (Hendler & Tran, 2001: p. 113)	[Late-stage embryos are positioned with their mouth and arms pressed against the wall of the bursa; (they) could take up nutrients secreted by the bursal wall through their mouth and tube feet] (Hendler & Tran, 2001: p. 121)	Hendler & Tran (2001)
	Amphipholis squamata [egg 100.0 µm] (Fell, 1946); [egg 0.1–0.15 mm, premetamorphosed embryo length 0.24 mm] (Hendler, 1975);	Brooding: in bursae.	[Wall of the bursa secretes nutritive substances] (Fell, 1946: p. 462)	[A nutritive substance… is directly absorbed by the embryo] (Fell, 1940: p. 173); [secretion…is absorbed directly by tissues of developing embryo] (Fell,	Fell (1940, 1946); Hendler (1975); Walker & Lesser (1989[1,2]); direct transport of nutrients was not shown; Byrne (1994); Khanna & Yadav (2005)

	[near-term juvenile disc size 800.0 µm] (Walker & Lesser, 1989);<br>[egg 100.0 µm, maximum disc ø 880.0 µm] (Byrne, 1994)			1946: p. 450);<br>[external epithelium of the embryo…can incorporate …aminoacids] (Walker & Lesser, 1989: p. 528);<br>[epithelium of the intestinal portion of the gut of near-term juveniles is slightly everted through the mouth and its elongated microvillae are pressed against the bursal cuticle] (Walker & Lesser, 1989: p. 522);<br>[PAS-positive material stored in vacuoles in the expanded bursal wall …next to the embryo…; potentially… nutrients (are) obtained from the symbiotic bacteria] (Byrne, 1994: p. 333)	
	Amphioplis japonicus [egg 0.1–0.15 mm, pre-metamorphosed embryo 0.24 mm] (Hendler, 1975);<br>[increase in disk ø in young from 0.72 mm to 1.4 mm] (Murakami, 1940)	Brooding: in bursae.			Murakami (1940); Hendler (1975)
	Amphipholis sp. [increase in disk ø in	Brooding: in bursae.			Murakami (1940); Hendler (1975);

	young from 0.72 mm to 1.4 mm] (Murakami, 1940);<br>[egg 0.1–0.15 mm, premetamorphosed embryo length 0.24 mm] (Hendler, 1975);<br>[ovarian ova near maturation 100.0 µm, juveniles (disk?) 500 µm in ø] (Oguro *et al*., 1982)				Oguro *et al*. (1982)
	Amphistigma minuta [one specimen…has a large brood juvenile occupying half the disc, and one small embryo] (O'Loughlin, 1991: p. 227)	Brooding: in bursae.			O'Loughlin (1991)
**Family Ophiodermatidae**	*Ophiurochaeta* sp. A [egg 680.0 µm, maximum ø of flattened embryonic disc 1000.0 µm]	Brooding.			Byrne (1991)
**Class Asteroidea**<br>**Family Asterinidae**	*Parvulastra vivipara* [egg 150.0 µm, larvae about 270.0 µm in length newly metamorphosed juveniles 314.0 µm in ø] (Byrne & Cerra, 1996);<br>[largest juveniles	Viviparity: in ovotestes.	[Extraembryonic nutrition].	Oophagy, embryophagy [intragonadal cannibalism], [pre-metamorphic develop-ment…may be augmented by nutrients present in the gonadal fluid] (Byrne & Cerra, 1996: p. 29)	Chia (1976);<br>Byrne (1996, 2006);<br>Byrne & Cerra (1996)

	emerging from the parent 1.8–5.0 mm in ø] (Byrne, 1996, 2006)				
	Parvulastra parvivivipara [larvae about 210.0 µm In length, newly metamorphosed juveniles 244.0 µm in ø] (Byrne & Cerra, 1996); [largest juveniles emerging from the parent up to 2.5 mm in ø] (Byrne, 1996); [egg 235.0 µm] (Byrne, 2006)	Viviparity: in ovotestes.	[Extraembryonic nutrition].	As above.	Byrne (1996, 2006); Byrne & Cerra (1996)
	Cryptasterina pacifica [egg 450.0 µm, juveniles at birth 900.0 µm] (Komatsu *et al*., 1990)	Viviparity: in ovotestes.			Komatsu *et al*. (1990); Byrne *et al*. (2003)
	Cryptasterina hystera [egg 440.0 µm, juveniles at birth 800.0 µm, intragonadal juveniles 1.0–4.0 mm] (Byrne *et al*., 2003; Byrne, 2005)	Viviparity: in ovotestes.		Embryophagy: [brood cannibalism occasionally occurs] (Byrne *et al*., 2003: p. 292); [sibling cannibalism..., a form of matrotrophy] (Byrne, 2005: p. 88)	Byrne *et al*. (2003); Byrne (2005)
**Family Asteriidae**	*Anasterias minuta* [eggs and early embryos mean ø 1.81±0.27 mm, juveniles at release, radius 1.39±0.15 mm] (S.D.)	Brooding in [external oral brood chamber].		[Connection cord is developed from a small preoral lobe at early stages of development; presence of yolk platelets and lipid inclusions in the connection	Gil *et al*. (2011)

				cord may indicate translocation of nutritive reserves from aborted eggs and abnormal embryos… of the brood mass] (Gil *et al*., 2011: pp. 2589, 2597)	
	Neosmilaster georgianus [egg 2.17±0.10 mm, advanced juvenile arm length is of at least 2.5 mm; between the earliest and most advanced stages of development there was a significant increase of 66% in the average individual dry weight] (Bosch & Slattery, 1999: p. 452)	Brooding: [in sub-oral brood chamber].		[Short cylindrical cord continuous with the presumptive oral region of the embryo; cannibalism] *via* cord is suggested (Bosch & Slattery, 1999: p. 452)	Bosch & Slattery (1999[2])
**Family Pterasteridae**	*Pteraster militaris* [egg ~1.1 mm, juveniles 2.1–4.5 mm; the mean energetic content of freshly spawned eggs was 5 times less than that of brooded juveniles] (McClary & Mladenov, 1990: p. 183) [vitellogenic oocytes 700.0–950.0 µm] (McClary & Mladenov,	Brooding: in aboral brood chamber.		Ingestion of maternal tissues, faeces, mucus and abortive young; [brooded juveniles are cannibalistic ectoparasytes] (McClary & Mladenov, 1990: p. 189)	McClary & Mladenov (1989, 1990[2])

	1989)				
**Family Stichasteridae**	*Smilasterias multipara* [egg ~1.0 mm, juveniles 3.0 mm]	Brooding: in stomach.			Komatsu *et al*. (2006)
**Family Xyloplacidae**	*Xyloplax medusiformis* [oocyte 150.0 µm, prenatal juveniles 560.0 µm]	Viviparity: in coelom.		[Development relies substantially on availability of dissolved nutrients from the coelom and possibly from the resorption of the tissues of the vestigial gut] (Rowe *et al*., 1988: p. 450)	Rowe *et al*. (1988)
	Xyloplax janetae	Viviparity: in coelom.			Mah (2006)
**Class Holothuroidea Family Synaptyidae**	*Leptosynapta clarki* [oocytes 200.0 to 240.0 µm develop to eight-tentacled pentacula stage] (Sewell & Chia, 1994); [juveniles after release 1.0–2.0 mm] (Sewell, 1994); [egg 299.3 µm, pentacula size at release 1.0–2.0 mm, increase in energetic content… is greater than 11×] (Sewell *et al*., 2006: p. 227)	Viviparity: in ovary =[in ovarian tubule].	Thickened ovarian wall [has the potential to be used as a route for the transfer of nutritive substances] (Sewell *et al*., 2006: p. 233); [matrotrophy].	[Nutrition for pentactulae …is provided…from the resorption of unfertilized eggs…, dead pentaculae…, (and gonadal) fluid in the area of (incubation)] (Sewell & Chia, 1994: p. 299)	Sewell (1994); Sewell & Chia (1994); Sewell (1996); Sewell *et al*. (2006[1,2])
	Synaptula hydriformis [mature egg 200.0 µm,	Viviparity: in perivisceral Coelom.		Ingestion of coelomocytes and molecules from	Clark (1898); Vaney (1925);

	often birth of young of 5.0 mm long, but coelomic juveniles can be much larger] (Clark, 1898);<br>[ovulated egg ~250.0 µm, juveniles 0.5–10.0 mm, increase in dry and organic weight of over 100-fold from the eggs to juveniles] (Frick, 1998)			coelomic fluid of the adult; [transepidermal…absorption of molecules across the body wall of the young] has been suggested (Frick, 1998: p. 175)	Sewell & Chia (1994); Frick (1998[2])
**Family Cucumariidae**	*Staurothyone inconspicua*<br>[free juveniles in a coelom from 1.0×1.0 mm to 6.0×4.0 mm and up to 7.0 mm, juveniles attached to a gonad 0.5×0.5 mm to 6.0×3.0 mm] (Materia *et al*., 1991)	Viviparity: in perivisceral coelom.		[Attachment of…juveniles by the oral end, and the accompanied shrinking of the gonad tubules suggest that the attached juveniles derive nutrients directly from the gonad] (Materia *et al*., 1991: p. 304);<br>[presumably embryos free in the coelom feed on coelomic fluid] (Sewell & Chia, 1994: p. 299)	Materia *et al*. (1991); Sewell & Chia (1994)
	Cucumaria georgiana<br>[eggs in ovary 1.0 mm, juveniles 3.5–3.7 mm long]	Brooding: in marsupia.			O'Loughlin (2001)
	Cucumaria sp.<br>[eggs (or embryos) in ovary 1.5 mm, well-differentiated juveniles 2.5 mm]	Brooding: in marsupia.			O'Loughlin (1994)

	‘*Cucumaria georgiana* group’ sp. [undifferentiated eggs or embryos 1.3 mm, differentiated embryos 3.0–4.0 mm]	Brooding: in marsupia.			O’Loughlin *et al*. (2009)
	‘*Cucumaria georgiana* group’ sp. [eggs in gonad tubules 1.5 mm, marsupial juveniles 2.0–3.0 mm]	Brooding: in marsupia.			O’Loughlin *et al*. (2009)
	Cucumaria ijimai [egg 0.5–0.55 mm, juveniles in length×width 2.0–7.0×1.0–4.0 mm]	Brooding: [in incubation pouches].			Ohshima (1915)
	Ocnus glacialis [egg 1.0 mm, juveniles 3.0–5⅓ mm] (Mortensen, 1894)	Brooding: [in incubation pouches].			Mortensen (1894); Vaney (1925)
	Microchoerus splendidus [eggs in gonad tubules 1.5 mm, marsupial juveniles 4.0 mm long]	Brooding: in marsupia.			O’Loughlin (2001)
	Pseudocnus laevigatus [eggs in gonad tubules 1.5 mm, undifferentiated eggs or embryos in marsupia 1.5 mm, differentiated juveniles 3.0 mm]	Brooding: in marsupia.			O’Loughlin (2001)
	Pseudocnus curatus [total dry weight	Brooding: in invaginations of a sole.			Turner & Rutherford (1976)

	increases from 169.0 to 190.0 µg/egg during embryogenesis]				
	Pseudocnus lamperti [egg 0.8 mm, length of the young 1.8–3.2 mm]	Brooding: in a pair of brood pouches on the ventral side.			Ohshima (1915)
	Stereoderma imbricata [egg 1.2 mm, juveniles 2.0–3.0 mm or more in length]	Brooding: in a pair of brood pouches on the ventral side.			Ohshima (1915)
	Pentocnus bursatus [ovarian eggs (or embryos) 0.3–0.8 mm, brood embryo 0.8 mm, juveniles 1.0–2.3 mm]	Brooding: [in intracoelomic membranous sacs].			O'Loughlin (1991); O'Loughlin & O'Hara (1992)
	Neocnus bimarsupiis [ovarian eggs (or embryos) 0.6–1.2 mm, brood embryo 0.7 mm, juveniles up to 1.5 mm]	Brooding: [in dorsal brood pouches].			O'Loughlin (1991); O'Loughlin & O'Hara (1992)
	Neocnus incubans [eggs 200.0–500.0 µm] (Cherbonnier, 1972); [juveniles 0.7–1.1 mm] (Alvà & Jangoux, 1992)	Brooding: in dorsal two-chambered [marsupium].			Cherbonnier (1972); Alvà & Jangoux (1992)
	Cladodactyla crocea [eggs 0.7 mm, juveniles 2.5–3.0 mm]	Brooding: [in incubation pouches].			Vaney (1925)
	Trachythyone parva [eggs 0.2 mm, juveniles 0.5–0.6 mm]	Brooding: in sac-like invaginations.			Vaney (1925)
	Psolidiella mollis	Brooding: in marsupial.			O'Loughlin *et al.* (2009)

	[undifferentiated eggs or (early) embryos 1.3 mm, differentiated embryos 3.0–4.0 mm]				
	Psolidiella adhaerens [eggs 0.5 mm, some embryos up to 0.8 mm long]	Brooding: in [pockets around oral cone].			O'Loughlin (1994)
	Neoamphicyclus lividus [egg 370.0 µm, incubated young from 3.5×1.8 mm to to 7.0×2.1 mm and up to 28.0×5.0 mm] (Hickman, 1978)	Viviparity: in perivisceral coelom.		[Embryos ingest coelomic fluid] (Sewell & Chia 1994: p. 299);<br>in a young [gut…was filled with coagulated body fluids] (Hickman, 1978: p. 31)	Hickman (1978);<br>Sewell & Chia (1994)
	Neoamphicyclus materiae [largest egg in gonad 0.5 mm, coelomic embryos from 0.5 mm to 9.0×3.0 mm] (Materia *et al*., 1991);<br>{strong difference in embryo size, no measurements or scale bar given} (O'Loughlin *et al*., 2009: Fig. 2d)	Viviparity: in perivisceral coelom.		Embryophagy: [intracoelomic brood auto-ingestion] (O'Loughlin *et al*., 2009: p. 217)	Materia *et al*. (1991);<br>O'Loughlin *et al*. (2009)
	Parathyonidium sp. [coelomic embryos 2.0–3.0 mm]	Viviparity: in perivisceral coelom.			O'Loughlin *et al*. (2009)
	Squamocnus niveus [eggs or embryos 0.7–0.8 mm long in sacs;	Brooding(?): [in intracoelomic sacs].			O'Loughlin & Alcock (2000)

	coelomic embryos uniformly 1.0 mm]				
	Psolidocnus amokurae [most mature juvenile 2.2×1.8 mm, eggs are considerably smaller] (O'Loughlin, 1994)	Brooding(?): [in dorsal coelomic chamber].			O'Loughlin (1994); O'Loughlin & Alcock (2000)
**Family Psolidae**	*Psolus charcoti* [egg 650 µm] (McEuen & Chia, 1991]; [eggs or embryos 1.0–1.8 mm, differentiating juveniles 3.5–4.0 mm, juveniles 6.0–8.0 mm long] (O'Loughlin, 2001; O'Loughlin *et al*., 2009)	Brooding: in marsupia.			McEuen & Chia (1991); O'Loughlin (2001); O'Loughlin et al. (2009)
	Psolus granulosus [eggs 0.5 mm, larvae 2.0 mm]	Brooding: in invaginations of a sole.			Vaney (1925)
	Psolus ephippifer [embryonic growth]	Brooding: in invaginations of a dorsal side.			Vaney (1925)
	Psolus dubious [eggs 0.83–1.38 mm, juveniles 0.80–2.30 mm]	Brooding(?): [pouch-like extensions of the anteriormost part of the intestine]			Gutt (1991)
	Lissothuria antillensis [egg 400 µm, released juveniles 1.0–1.5 mm] (Miller, 1985)	Viviparity: in perivisceral coelom.			Miller (1983, 1985); McEuen & Chia (1991)
**Family Deimaidae**	*Oneirophanta mutabilis affinis* [eggs up to 0.2 mm]	Viviparity: in ovary.	Ovarian wall: [vacuolated tissue] (Hansen, 1975: p. 200)	Oophagy: [in the early stages the young may feed on unfertilized eggs];	Hansen (1968, 1975)

	(Hansen, 1975);<br>[the young increase in size from 7 to 30 mm in ovaries emptied of eggs] (Hansen, 1968: p. 1063)			and histotrophy: nourishment by [nutritive fluid secreted into the ovaries] (Hansen, 1968: p. 1063)	
**Family Chiridotidae**	*Chiridota rotifera* {early embryo ~500 µm} [largest incubated young 3.0 mm]	Viviparity: in perivisceral coelom.			Clark (1910)
	Sigmodota contorta [eggs ~0.33 mm, young 2.27–3.0 mm in length, 0.54–0.70 mm in width] (Ludwig, 1898)	Viviparity: in ovarian tubules.			Ludwig (1898);<br>Vaney (1925);<br>Hansen (1968)
**Family Phyllophoridae**	*Pachythyone rubra* [length of 5-tentacle juveniles 2.0 mm, length of 10-tentacles juvenile 7.0 mm] (Clark, 1901)	Viviparity: in perivisceral coelom.			Clark (1901);<br>Vaney (1925)
**Family Sclerodactylidae**	*Afrocucumis africana* [coelomic young from 1.4×0.6 mm to 10.0×2.5 mm]	Viviparity: in perivisceral coelom.			Ohshima (1916)
**Class Echinoidea**<br>**Family Urechinidae**	*Antrechinus mortenseni* [ovarian egg 1.7 mm, juvenile 4.5 mm]	Brooding: in brood pouches.		[Transepidermal nutrient absorption] suggested (Mooi & David, 1993: p. 75)	David & Mooi (1990);<br>Mooi & David (1993)
	Antrechinus nordenskjoeldi	Brooding: in brood pouches.			David & Mooi (1990);<br>Mooi & David (1993)

	[ovarian egg 1.5 mm, juvenile 4.5 mm]				
**Family Schizasteridae**	*Abatus cordatus*<br>[eggs 1.3–1.95 mm, embryos 2.2–2.7 mm] (Magniez, 1980);<br>[egg 1.34 mm, juveniles up to 2.02 mm, 13-fold increase in ash amount] (Lawrence *et al*., 1984; Schatt, 1985);<br>[dry weight increase from 0.7 mg to 2.5 mg] (Schatt, 1988);<br>[egg 1300.0 µm, juveniles 1.950–2.0 mm] (Schatt & Féral, 1996)	Brooding: in brood pouches.			Magniez (1980);<br>Lawrence *et al*. (1984[2]);<br>Schatt (1985, 1988[2]);<br>Schatt & Féral (1996)
	Abatus ingens<br>[juveniles without spines – test ø 1.0 mm,<br>fully spined juveniles – test ø 2.0–3.0 mm]	Brooding: in brood pouches.			Lockhart *et al*. (1994)
	Abatus shakletoni<br>[mean egg ø 1.28 mm, 8-10-fold increase in ash level] (McClintock & Pearse, 1986);<br>[egg average ø 1.0 µm, stage I juvenile mean length 1.6 mm,<br>stage II juvenile mean length 2.8 mm]	Brooding: in brood pouches.			McClintock & Pearse (1986);<br>Pearse & McClintock (1990);<br>Schinner & McClintock (1993)

	(Schinner & McClintock, 1993)				
	Abatus nimrodi [mean egg ø 1.97 mm, 8-10-fold increase in ash level] (McClintock & Pearse, 1986); [egg average ø 1.4 µm, stage I juvenile mean length 2.0 mm, stage II juvenile mean length 4.3 mm] (Schinner & McClintock, 1993); [spineless juveniles test ø 1.5 mm, emergent-spine juveniles 2.5 mm, spined juveniles 4.0–5.0 mm] (Lockhart *et al*., 1994)	Brooding: in brood pouches.			McClintock & Pearse (1986); Pearse & McClintock (1990); Schinner & McClintock (1993); Lockhart *et al*. (1994)
**Paleopneustina incertae sedis B**	*Amphipneustes lorioli* [egg 1302.0–1971.0 µm, juveniles up to 5.6 mm]	Brooding: in brood pouches.			Galley *et al*. (2005)
**Class Crinoidea Family Comasteridae**	*Comatilia iridometriformis* [egg or embryo(?) ~400.0 µm, larval length 1–1.6 mm]	Viviparity: in ovary.		Oophagy and/or embryophagy	Messing (1984)
**Family Antedonidae**	*Isometra vivipara* [egg ~0.3 mm, larval length 0.5–0.6	Brooding: in marsupia.			Mortensen (1918, 1920)

	mm] (Mortensen, 1920)				
**Family Notocrinidae**	*Notocrinus virilis* [eggs 200.0–300.0 µm, larval length ≤1.8 mm] (Mortensen, 1920)	Brooding: in marsupia.	[Nourishment from mother animal] (Mortensen, 1920: p. 50)	[Some of eggs are destined to serve as nourishment for developing embryos; …absorption (of disintegrated eggs) through the skin] (Mortensen, 1920: pp. 49-50)	Mortensen (1918, 1920)
**<u>Phylum Acoelomorpha</u>** **Order Acoela** **Family Childiidae**	*Childia vivipara* {largest egg 71.0×95.0 µm, largest embryo 207.0×222.0 µm}	Viviparity: in [embryonic vesicle, thick outer membrane] inside parenchyma.			Tekle *et al*. (2006)
**Family Diopisthoporidae**	*Diopisthoporus brachypharyngeus* (as Acoela gen. spec. 2) {mature oocyte 46.0 µm, multicellular embryo 90.0×76.6 µm}	Viviparity: in [embryonic vesicle] inside parenchyma.			Apelt (1969)
**Family Convolutidae**	*Heterochaerus carvalhoi* {largest egg 75.0×112.0 µm, largest embryo 168.0×17.08 µm}	Viviparity: in [embryonic vesicle] inside parenchyma.			Marcus (1952)
**Family Isodiametridae**	*Avagina vivipara* [mature ova 108.0×89.0 µm, advanced embryos	Viviparity: in [cavity, delicate membrane] inside parenchyma;			Hickman (1956)

	111.0×84.0 µm]	(lecithotrophy *versus* incipient matrotrophy?)			
**<u>Phylum Chordata</u>**<br>**Subphylum Urochordata**<br>**Class Ascidiacea**<br>**Family Styelidae**	*Botrylloides simodensis* [oocyte 180.0 µm]	Brooding: in atrial brood pouch.	[Lining epithelium of the (brood) pouch]; [extraembryonic nutrition... seems to be very limited] (Mukai *et al*., 1987: p. 274)	[Epidermis of the embryo].	Mukai *et al*. (1987)
	Botrylloides violaceus [oocyte 60.0 µm] (Mukai *et al*., 1987); [oocyte 80.0 µm] (Zaniolo *et al*., 1998)	Brooding: [in brood pouch in colonial tunic].	[In the lining epithelium of the brood pouch, cells change from cuboidal to rather columnar] (Mukai *et al*., 1987: p. 270); [synthesis, storage and secretion towards the embryo of proteinaceous materials and glycogen] (Zaniolo *et al*., 1998: p. 11); [extraembryonic nutrition].	[Epidermis of the embryo] (Mukai *et al*., 1987: p. 275)	Mukai *et al*. (1987); Zaniolo *et al*. (1998[1]**)**
	Botrylloides lenis [oocyte 90.0 µm]	Brooding: in atrial brood pouch.	[Epithelium of the brood pouch]; [extraembryonic nutrition].	[Epidermis of the embryo].	Mukai *et al*. (1987)
	Botrylloides leachi [oocyte 300.0 µm] (Zaniolo *et al*., 1994)	Brooding: [in brood pouch].	[Placental epithelium] (Berrill, 1947: p. 399); [the columnar lining cells of the brood pouch become vacuolated with irregular apical surfaces suggesting their secretory nature, and	[Glucose consumption] (Zaniolo *et al*., 1994)	Berrill (1947); Mukai *et al*. (1987); Zaniolo *et al*. (1994[1]**)**

			the fluid in the lumen of the pouch contains a coagulable substance] (Mukai *et al*., 1987: p. 275); [placentation, extra-embryonic nutrition] (Berrill, 1947: p. 399); [apocrine secretion] by brood pouch epithelial cells; …embryo-parent nutritional exchange] (Zaniolo *et al*., 1994: p. 216)		
	Botryllus primigenus	Brooding: [in brood pouch].	[Some form of extraembryonic nutrition] (Mukai *et al*., 1987: p. 264)		Mukai *et al*. (1987)
**Family Holozoidae**	*Hypsistozoa fasmeriana* {early gastrula 0.1 mm} [tadpole: head 2.9×1.6 mm, tail 3.9×1.0 mm]	Viviparity: in oviducal brood pouch.	[Cells lining the brood pouch; extra-embryonic nutrition].	At early stages – *via* endodermal tubes, at later stages – absorption by ectotrophe (extra-embryonic membrane).	Brewin (1956)
**Class Thaliacea Order Salpida Family Salpidae**	All species (1 family, ~48 species); examples:	Viviparity at early stages (in a follicle=uterine sac), later brooding (in incubation fold of atrial epithelium).	[The placenta…is thickened uterine wall in contact with the genital blood sinus. This wall becomes the placental roof…(that) turns into a syncytium through which feeding of the embryo proceeds] (Godeaux, 1990: p. 466)		Godeaux (1990); Godeaux *et al*. (1998)

	Salpa fusiformis	Viviparity at early stages, later brooding.	[Placental tissue… derived from the follicle cells] (Bone *et al*., 1985: p. 54)		Sutton (1960);<br>Bone *et al*. (1985)
	Salpa maxima	Viviparity at early stages, later brooding.	[placenta]		Berril (1950)
	Cyclosalpa pinnata	Viviparity at early stages, later brooding.	[Placenta, of follicular origin] (Berril, 1950: p. 566)		Brooks (1893);<br>Brooks & Metcalf (1893);<br>Berril (1950)
	Metcalfina hexagona	Viviparity at early stages, later brooding.	[Placenta].		Brooks (1893);<br>Brooks & Metcalf (1893)
	Pegea confoederata	Viviparity at early stages, later brooding.	[Placenta].		Berril (1950)
	Ihlea punctata	Viviparity at early stages, later brooding.	[Placenta].		Bone *et al*. (1985)
	Thalia democratica [oocyte 100.0 µm, Godeaux 1990]	Viviparity at early stages, later brooding embryo [not completely enclosed] by [incubation fold] (Sutton, 1960: p. 276)	[Placenta].		Huxley (1851);<br>Berril (1950);<br>Bone *et al*. (1985);<br>Godeaux (1990)
